# Expansion of phenotypic spectrum of *MYO15A* pathogenic variants to include postlingual onset of progressive partial deafness

**DOI:** 10.1186/s12881-018-0541-9

**Published:** 2018-02-27

**Authors:** Mun Young Chang, Chung Lee, Jin Hee Han, Min Young Kim, Hye-Rim Park, Nayoung Kim, Woong-Yang Park, Doo Yi Oh, Byung Yoon Choi

**Affiliations:** 1 Department of Otorhinolaryngology-Head and Neck Surgery, Chung-Ang University College of Medicine, 102 Heukseok-ro, Dongjak-gu, 06973 Seoul, Republic of Korea; 2 Samsung Genome Institute, Samsung Medical Center, 81 Irwon-ro, Gangnam-gu, 06351 Seoul, Republic of Korea; 3 Department of Health Sciences and Technology, SAIHST, Sungkyunkwan University, 2066 Seobu-ro, Jangan-gu, 16419 Suwon, Republic of Korea; 4 Department of Otorhinolaryngology, Seoul National University Bundang Hospital, 82 Gumi-ro 173 beon-gil, Bundang-gu, 13620, Seongnam, 463-707 Republic of Korea; 5 Department of Molecular Cell Biology, School of Medicine, Sungkyunkwan University, 2066 Seobu-ro, Jangan-gu, 16419 Suwon, Republic of Korea; 6 Wide River Institute of Immunology, Seoul National University College of Medicine, 101 Dabyeonbatgil, 25159 Hongcheon, Republic of Korea

**Keywords:** *MYO15A*, Phenotype, Deafness, Pathogenic variant

## Abstract

**Background:**

*MYO15A* variants, except those in the N-terminal domain, have been shown to be associated with congenital or pre-lingual severe-to-profound hearing loss (DFNB3), which ultimately requires cochlear implantation in early childhood. Recently, such variants have also been shown to possibly cause moderate-to-severe hearing loss. Herein, we also demonstrate that some *MYO15A* mutant alleles can cause postlingual onset of progressive partial deafness.

**Methods:**

Two multiplex Korean families (SB246 and SB224), manifesting postlingual, progressive, partial deafness in an autosomal recessive fashion, were recruited. Molecular genetics testing was performed in two different pipelines, in a parallel fashion, for the SB246 family: targeted exome sequencing (TES) of 129 known deafness genes from the proband and whole exome sequencing (WES) of all affected subjects. Only the former pipeline was performed for the SB224 family. Rigorous bioinformatics analyses encompassing structural variations were executed to investigate any causative variants.

**Results:**

In the SB246 family, two different molecular diagnostic pipelines provided exactly the same candidate variants: c.5504G > A (p.R1835H) in the motor domain and c.10245_10247delCTC (p.S3417del) in the FERM domain of *MYO15A*. In the SB224 family, c.9790C > T (p.Q3264X) and c.10263C > G (p.I3421M) in the FERM domain were detected as candidate variants.

**Conclusions:**

Some recessive *MYO15A* variants can cause postlingual onset of progressive partial deafness. The phenotypic spectrum of DFNB3 should be extended to include such partial deafness. The mechanism for a milder phenotype could be due to the milder pathogenic potential from hypomorphic alleles of *MYO15A* or the presence of modifier genes. This merits further investigation.

**Electronic supplementary material:**

The online version of this article (10.1186/s12881-018-0541-9) contains supplementary material, which is available to authorized users.

## Background

*MYO15A,* a causative gene of DFNB3 (OMIM 600316) [1], is a frequently detected deafness gene. Friedman et al., in Bali, first reported that the frequency of autosomal recessive hearing loss caused by the *MYO15A* pathogenic variant was about 2% [2]. Thereafter, the frequency of *MYO15A* pathogenic variant was reported in up to 9.9% of deafness cases in Turkey [3]. In our previous study, *MYO15A* pathogenic variant was reported at a frequency of 2.1% in nonsyndromic autosomal recessive deafness, which was the fourth most common deafness gene in Korea, following *SLC26A4, GJB2* and *CDH23* [4]. Sequentially, several studies have been performed to investigate the effects of *MYO15A* pathogenic variants on hearing loss [5–9].

The role of myosin XVA, which is encoded by *MYO15A*, includes the graded elongation and maintenance of stereocilia and actin-organization in the inner ear hair cells. These are both essential for normal auditory function. Therefore, *MYO15A* pathogenic variants were initially thought to induce congenital severe-to-profound hearing loss [1, 10–15]. However, it was later discovered that the phenotypes of *MYO15A* pathogenic variants varied depending on the affected domain. The variation of phenotypes according to the affected domain has been explained by the existence of two isoforms -- a class 1 isoform with the N-terminal domain, which is encoded by exon 2, and a class 2 isoform with no N-terminal domain [16, 17]. The pathogenic variant in N-terminal domain affects only the class 1 isoform without affecting the class 2 isoform [18]. The class 2 isoform is present in the human inner ear [17]. Therefore, pathogenic variants in the N-terminal domain are known to cause minor deficiencies in the inner ear, resulting in amilder auditory phenotype, when compared with other pathogenic variants in *MYO15A* [5–7]. Conversely, *MYO15A* pathogenic variants that reside in the regions shared by both isoforms are known to cause congenital or prelingual severe-to-profound hearing loss [7].

Recently, Naz et al. reported that *MYO15A* pathogenic variants, which had previously been thought to only cause profound hearing loss, may cause moderate-to-severe hearing loss [9]. We also found two families with *MYO15A* pathogenic variants in the motor and FERM domains, which were expected to cause profound hearing loss. They showed postlingual onset of bilateral symmetrical, partial deafness with significant residual hearing at low frequencies. Based on these results, we suggest that *MYO15A* may be a causative gene responsible for the postlingual onset of progressive partial deafness, which in turn requires the expansion of the phenotypic spectrum of *MYO15A* pathogenic variants.

## Methods

### Human subjects

**Fig. 1:**
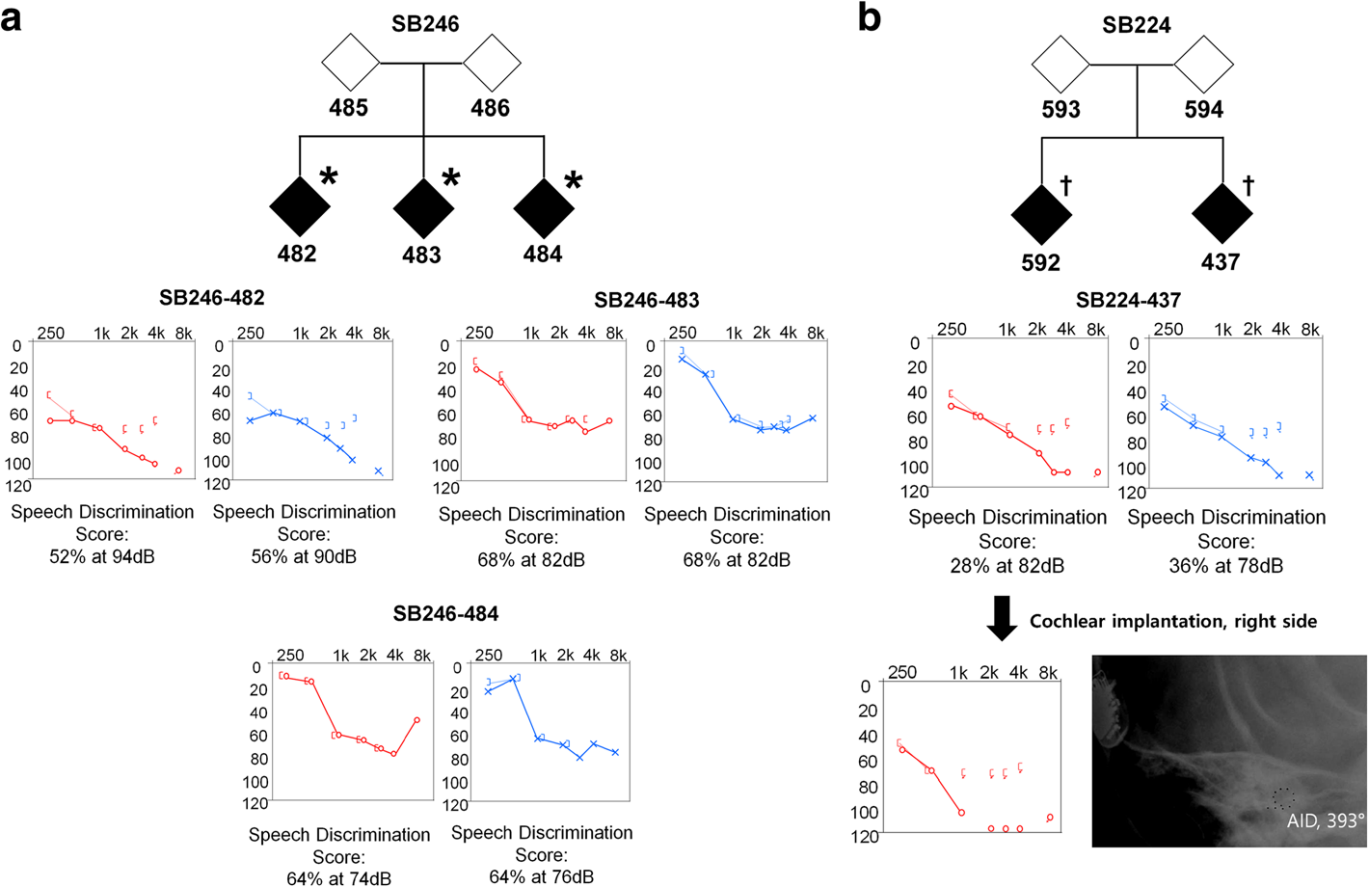
Pedigree and pure-tone audiograms of family SB246 (**a**) and 224 (**b**). SB246–482 and SB224–437 showed bilateral, severe, and symmetrical SNHL. SB246–483 and 484 showed bilateral, moderate, and symmetrical SNHL. In all subjects, hearing at low frequencies was significantly preserved. SB224–437 had cochlear implantation at the age of 20. During the operation, insertion of Med-El device, Flex 24 (Angular insertion depth [28] of 393°) into the cochlea was performed, and residual hearing of low frequencies was substantially preserved. *, subjects who underwent targeted exome sequencing of 129 known deafness genes and whole exome sequencing; †, subjects who underwent targeted exome sequencing of 129 known deafness

All procedures in this study were approved by the Institutional Review Boards of Seoul National University Hospital (IRBY-H-0905-041-281) and Institutional Review Boards of Seoul National University Bundang Hospital (IRB-B-1007-105-402). Written informed consent was obtained from all participants. Two multiplex Korean families (SB246 and SB224), with the segregation of postlingual, bilaterally symmetrical, partial hearing loss in an autosomal recessive fashion, were included in this study. The first family (SB246) was comprised of five individuals, three of whom participated in the study; the second family (SB224) was comprised of four individuals, all of whom participated. Two generations were included in each family (Fig. 1). Phenotypic evaluations included medical and developmental history interviews, physical examinations, and audiometric evaluation.

### Audiometric evaluation

Pure-tone audiometry was performed on SB246–482, 483, and 484 as well as on SB224–437, in accordance with the standard protocols. The air and bone conduction thresholds were obtained at frequencies of 250–8000 Hz. The hearing loss range was divided into three parts: low frequency, 250–500 Hz; mid frequency, 1–2 kHz; and high frequency, 4–8 kHz [19]. The mean levels of hearing loss for all frequencies as well as low, mid, and high frequencies were calculated.

### Molecular genetic test

In the SB246 family, molecular genetic testing was performed in two different pipelines, in a parallel fashion. The first pipeline was targeted exome sequencing (TES) of 129 known deafness genes from the proband, followed by a segregation study using Sanger sequencing; the second pipeline was whole exome sequencing (WES) in the three affected subjects. In the SB224 family, TES of 129 known deafness genes was performed from the proband, followed by a segregation study using Sanger sequencing.

### Targeted exome sequencing

TES and bioinformatics analyses were performed, as previously described [20–25]. The DNA samples from SB246–482 and SB224–437 underwent TES of 129 known deafness genes (TRS-129) by Otogenetics (Norcross, GA, USA) (Additional file 1: Table S1). The acquired reads were mapped onto the UCSC hg19 reference genome assembly, using Lasergene 14 software package (DNASTAR, Madison, WI, USA) (Additional file 2: Table S2). Further bioinformatics analyses were performed to identify all variants. As a basic filtering step, non-synonymous single nucleotide polymorphisms (SNPs) with read depths ≥20 were chosen. The known disease-causing SNPs or SNPs with global minor allele frequency (MAF) ≤ 0.002 were chosen. Global MAF was checked using several databases, including 1000 Genomes, Exome Aggregation Consortium (ExAC), and NHLBI Grand Opportunity Exome Sequencing Project (GO-ESP). These non-synonymous SNPs were compared against the Korean Reference Genome Database (KRGDB), that consists of 622 Korean individuals (1,244alleles) (http://152.99.75.168/KRGDB/menuPages/firstInfo.jsp). SNPs with allele frequency < 0.005 were chosen. Inheritance patterns were checked and SNPs that did not coincide with the autosomal recessive pattern were excluded. To predict the pathogenicity of each variant, SIFT, PolyPhen-2 analyses, and Pathogenic variantTaster were performed. Pathogenic variants are described in the context of the American College of Medical Genetics and Genomics (ACMG) 2015 guidelines [26]. The evolutionary conservation of the amino acid sequence was estimated using the GERP++ score in the UCSC Genome Browser (http://genome.ucsc.edu/). The variants that were predicted as benign by silico prediction were excluded. The remaining SNPs were validated in other family members (SB246–483, 484, 485 and 486 and SB224–592, 593 and 594) by Sanger sequencing.

### Whole exome sequencing

DNA samples from SB246–482, 483, and 484 were subjected to WES by Macrogen (Seoul, South Korea) (Additional file 3: Table S3). First, we filtered out the variants in non-coding regions, as well as synonymous variants in coding regions. Second, the variants with MAF < 1% were selected based on Exome Sequencing Project 6500 (ESP6500), 1000 Genomes Project (1000G), ExAC, and our in-house database containing the exomes of 192 Korean individuals. Third, following the inheritance pattern, homozygous variants and compound heterozygote variants with enough read depths (> 10×) and a genotype quality (> 20) were selected. Finally, to exclude the variants without clinical significance, flagged SNPs based on dbSNP ID (dbSNP 147) were selected. The relation of selected genes with diseases or functions was identified through previous studies. The variants related to hearing loss were selected. Moreover, rigorous bioinformatics analyses encompassing the structural variations were performed to investigate the causative variants. Copy number variations (CNV) were calculated by EXCAVATOR2 [27] with 20 k window size; the pooling mode and in-house CNV tool were used to estimate the log2 normalized depth ratio of each targeted region. Finally, we selected CNVs containing hearing loss gene or locus.

## Results

### Auditory phenotype

Pure-tone audiograms for the affected individuals are presented at Fig. 1. SB246–482 and SB224–437 showed bilateral, severe, and symmetrical sensorineural hearing loss (SNHL). SB224–437 showed a positive stapedial reflex at the time of toddler, suggesting that the subject did not have severe SNHL at that time (data not shown). Due to the progression of SNHL into a severe degree, especially in mid-to-high frequencies, SB224–437 underwent cochlear implantation in his/her twenties. During the operation, an insertion of Med-El device, Flex 24 (Angular insertion depth [28] of 393°) rather than Flex 28 into the cochlea, was performed, aiming to preserve low frequency hearing. Consequently, residual hearing of low frequencies was preserved substantially (Fig. 1b). Pure-tone audiometry was not performed for SB224–592 at our hospital. However, the pure-tone audiogram from another hospital showed that SB224–592’s hearing thresholds were 75 dB HL on both sides, and SB224–592 used bilateral hearing aids.

Interestingly, SB246–483 and 484 showed bilateral partial deafness, characterized by normal thresholds in low frequencies, but with severe SNHL in high frequencies. In SB246–482 and SB224–437, the mean threshold level for high frequencies was the highest, followed by mid and low frequencies. In all subjects, hearing at low frequencies was significantly preserved.

### Targeted exome sequencing

**Fig. 2:**
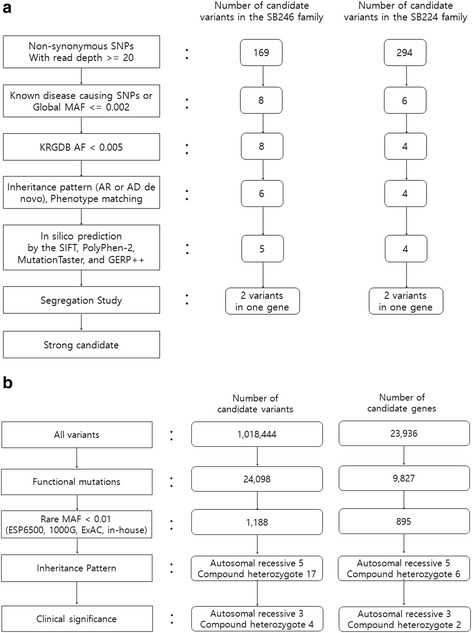
Schematic flowchart of filtering of causative variants in this study. **a** Targeted exome sequencing. **b** Whole exome sequencing. AR, autosomal recessive; AD, autosomal dominant

TES was performed in SB246–482 and SB224–437. The reads were aligned to the human reference genome (GRCh37/hg19), using SeqMan NGen, and the targeted variants were analyzed using ArrayStar software (version 14.1.0; DNASTAR, WI, USA). Bioinformatics analyses were performed (Fig. 2a). After the basic filtering step, eight and six variants were selected as candidate pathogenic variants for SB246–482 and SB224–437, respectively.

**Fig. 3:**
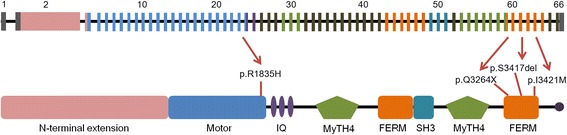
The locations of *MYO15A* variants

**Fig. 4:**
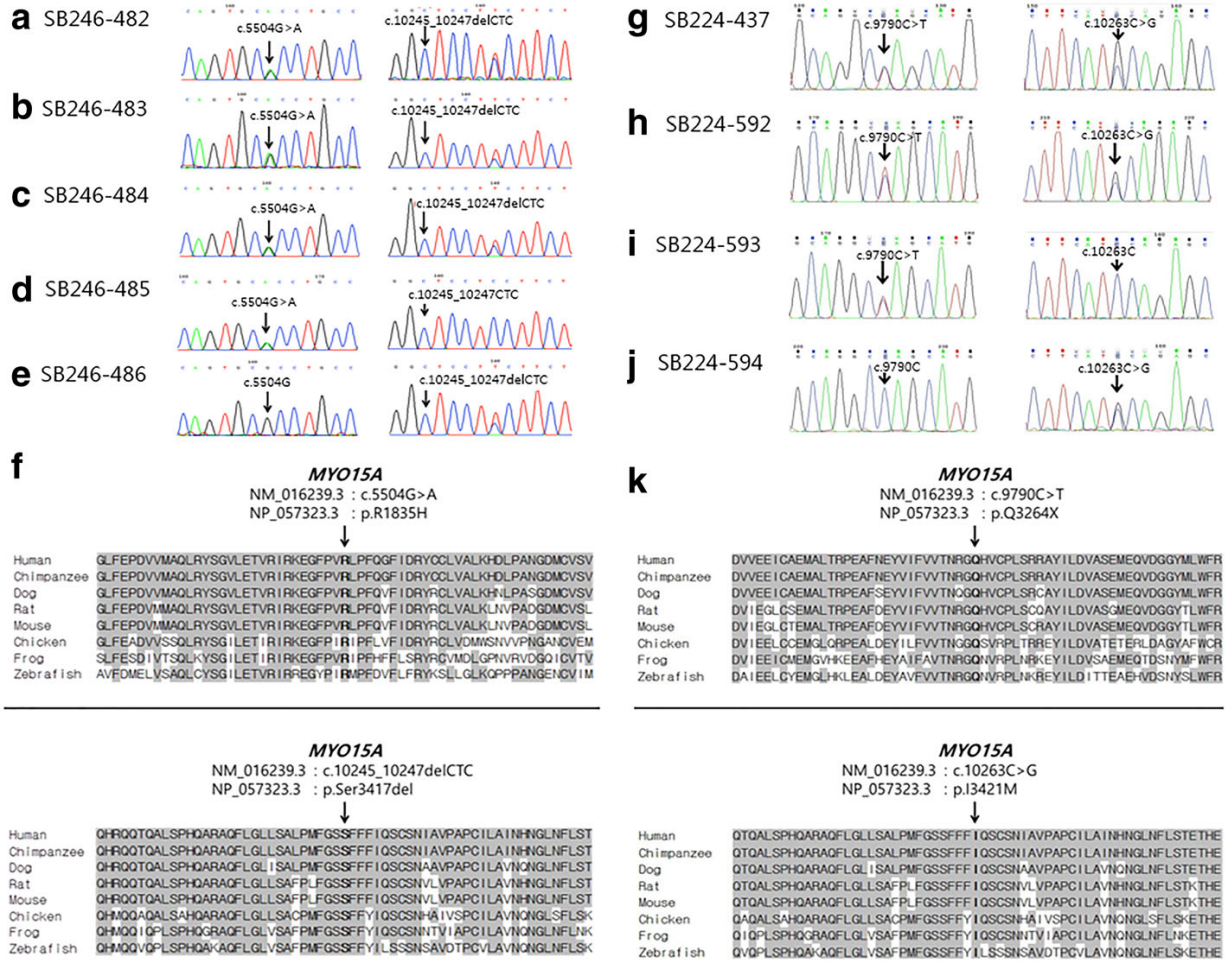
Segregation of *MYO15A* variants in two families, SB246 and 224. **a**-**c** Sanger sequencing traces for c.5504G > A (p.R1835H) + c.10245_10247delCTC (p.S3417del) compound heterozygote (SB246–482, 483 and 484). **d** Sanger sequencing traces for c.5504G > A carrier (SB246–485). **e** Sanger sequencing traces for c.10245_10247delCTC carrier (SB246–486). **f** Conservation of mutant residues among the orthologs from several species; p.R1835 and p.S3417 are conserved among all species, ranging from humans to zebrafish. **g** and **h** Sanger sequencing traces for c.9790C > T (p.Q3264X) + c.10263C > G (p.I3421M) compound heterozygote (SB224–437 and 592). **i** Sanger sequencing traces for c.9790C > T carrier (SB224–593). **j** Sanger sequencing traces for c.10263C > G carrier (SB224–594). **k** Conservation of mutant residues among the orthologs from several species; p.Q3264 and p.I3421 are conserved among all species, ranging from humans to zebrafish

As the inheritance pattern was autosomal recessive, variants that did not follow an autosomal recessive inheritance pattern were excluded. Through in silico prediction, a benign variant was excluded. Lastly, candidate variants were validated using Sanger sequencing in parents (SB246–485 and 486) and siblings (SB246–483 and 484) of SB246–482, as well as in parents (SB224–593 and 594) and sibling (SB224–592) of SB224–437. Only two candidate variants from a single gene, *MYO15A,* survived the filtering steps in each family (Fig. 2a). In the SB246 family, c.5504G > A (p.R1835H) and a single aminoacid deletion, c.10245_10247delCTC (p.S3417del), remained after the final filtering step (Table 1 and Fig. 3). The parents (SB246–485 and 486) of SB246–482 were heterozygous for c.5504G > A (p.R1835H) and c.10245_10247delCTC (p.S3417del), respectively (Fig. 4a). Moreover, p.R1835 and p.S3417 were well-conserved in several species, and in silico prediction supported the pathogenicity of these variants. In the SB224 family, a nonsense variant, c.9790C > T (p.Q3264X), and a missense variant, c.10263C > G (p.I3421M), remained (Table 1). The parents (SB224–593 and 594) of SB224–437 were heterozygous for c.9790C > T (p.Q3264X) and c.10263C > G (p.I3421M), respectively (Fig. 4b). Furthermore, p.Q3264 and p.I3421 were well-conserved in several species, and in silico prediction supported the pathogenicity of these variants.

**Table 1:** *MYO15A* variants identified in this study

Family	Nucleotide change	Amino acid change	Domain	GERP++	PhyloP	In silico prediction			MAF in ExAC	MAF in KRGDB	Classification of pathogenic variants	Published reference (PMID)
						PP2	S	MT				
SB246	**c.5504G > A**	**p.R1835H**	myosin motor	5.78	6.226	P	D	D	0.00002	ND	PP1	This study
	c.10245_10247delCTC	p.S3417del	FERM	1st:2.43, 2nd:5.81, 3rd:5.81	1st:0.852, 2nd:5.08, 3rd:6.153	NA	D	D	0.00003	ND	PVS1	25,792,667,27,375,115, This study
SB224	**c.9790C > T**	**p.Q3264X**	FERM	5.61	4.314	NA	NA	D	ND	ND	PVS1	This study
	c.10263C > G	p.I3421M	FERM	2.74	1.404	P	D	D	0.00003	0.000804	PP1	23,967,202, This study

Nomenclature is based on NCBI accession number NM_016239.3. Pathogenic variants are described in the context of the American College of Medical Genetics and Genomics (ACMG) 2015 guidelines [26]

Bold font: novel pathogenic variant

Conservation tools: GERP++ score in the UCSC Genome Browser (http://genome-asia.ucsc.edu/);PhyloP score from the Mutation Taster (http://www.mutationtaster.org/), in silico prediction tools: *PP2* Polyphen-2 (http://genetics.bwh.harvard.edu/pph2/index.shtml), *S* SIFT (http://sift.jcvi.org/www/SIFT_chr_coords_submit.html) or SIFT-indels2 (http://sift.bii.a-star.edu.sg/www/SIFT_indels2.html); *MT* Mutation Taster, *ExAC* Exome Aggregation Consortium (http://exac.broadinstitute.org/), *KRGDB* Korean Reference Genome DB (http://152.99.75.168/KRGDB/menuPages/firstInfo.jsp), *P* predicted probably damaging;D, either disease causing or damaging, *NA* an abbreviation for not applicable, *ND* not detected

### Whole exome sequencing

**Fig. 5:**
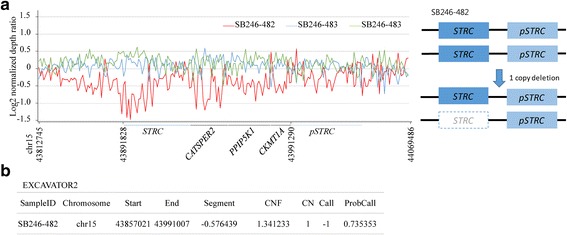
Copy number variant identification by in-house CNV tool and EXCAVATOR2 with WES. **a** The horizontal axis indicates chromosomal position, and the vertical axis indicates the log2 normalized depth ratio; 0 indicates 2 copies and − 1.0 indicates 1 copy deletion, respectively. The red line indicates SB246–482, light-blue line indicates SB246–483, and green line indicates SB246–484, respectively. A heterozygote deletion in the *STRC* and *CATSPER2* region is seen in only SB246–482. The hypothesized mechanisms for the event is shown to the right. **b** The result of EXCAVATOR2 also indicates 1 copy deletion in the region where *STRC* and *CATSPER2* as well as *PPIP5K1* and *CKMT1A* were located in

Independently of molecular genetic testing using TES, WES of genomic DNA from three affected subjects (SB246–482, 483 and 484) in the SB246 family was performed in a parallel fashion. Following the basic filtering step, 1188 variants from 895 genes were selected as candidate pathogenic variants (Fig. 2b). The inheritance pattern was regarded as autosomal recessive, and variants that did not fit for this inheritance pattern were excluded. After excluding the variants without clinical significance, seven variants from five genes remained (Fig. 2b and Table 2). Among the five genes, only *MYO15A* was related to hearing loss (Table 2). Two candidate variants, c.5504G > A and c.10245_10247delCTC from a single gene, *MYO15A*, remained. In addition, a large heterozygous genomic deletion involving *STRC* was detected from SB246–482; however, it did not segregate among the other two affected subjects. In the evaluation of CNV, one copy number alteration residing in *STRC* and *CATSPER2,* which are both known hearing loss genes, was detected by both EXCAVATOR2 and in-house CNV tool from only SB246–482 (Fig. 5a, b). The deletion of *STRC* and *CATSPER2* of SB246-482, if in a homozygous form, could be pathogenic, leading to dieafness-infertility syndrome.

**Table 2:** Results of whole exome sequencing

Inheritance pattern	Gene	Exonic function	Relation in disease or functions	OMIM	Reference
Autosomal recessive	*PER3*	Non-frame shift deletion	FASPS3	616,882	Zhang et al.
	*LNP1*	Non-frame shift insertion	unknown	–	–
	*FADS6*	Non-frame shift insertion	Fatty acid metabolism	–	–
Compound heterozygote	*ABCA2*	Missense/missense	Macrophage lipid metabolism	–	Kaminski et al.
	*MYO15A*	Missense/Non-frame shift deletion	DFNB3	600,316	Wang et al., Liburd et al., Riahi et al.

## Discussion

Although TES of 129 genes in the deafness panel is an efficient and convenient tool for detecting known causative variants, pathogenic variants from a novel deafness gene may not be detected using only TES. To explore and minimize such a possibility, we performed genetic testing via two diagnostic pipelines, TES and WES, in a parallel fashion for the SB246 family. Filtering of candidate variants through these two pipelines indicated the same result, strongly supporting our molecular diagnosis. Moreover, certain genes, such as *OTOF* and *STRC*, have been reported not to be fully covered by next generation sequencing [29, 30]. To overcome this, we modified the mapping quality of bioinformatics analysis. Furthermore, we excluded the possibility of structural variations involving *STRC* and *CATSPER2*, which was reported to be an important molecular etiology of SNHL in Japan [30]. In the SB224 family, two variants, c.9790C > T (p.Q3264X) and c.10263C > G (p.I3421M), of *MYO15A* survived at the final filtering step of TES analyses; c.9790C > T was a nonsense variant causing truncation of the protein, and c.10263C > G has already been reported to be pathogenic. Therefore, it is most likely that hearing loss from SB224–437 was attributed to these two *MYO15A* variants. Sequentially, WES was not performed in SB224.

The conservation and pathogenicity prediction study also strengthened our hypothesis that *MYO15A* pathogenic variants, c.5504G > A (p.R1835H) and c.10245_10247delCTC (p.S3417del), in the SB246 family and c.9790C > T (p.Q3264X) and c.10263C > G (p.I3421M), in the SB224 family were the causative pathogenic variants of hearing loss. Among the four *MYO15A* pathogenic variants discovered here, c.5504G > A and c.9790C > T were novel variants; c.9790C > T was a nonsense pathogenic variant, and c.5504G > A resided in the motor domain. The motor domain is one of the most important domains in myosin XVA. Sequentially, it is reasonable to infer that variants in the motor domain may lead to profound hearing loss. c.10245_10247delCTC and c.10263C > G resided in the FERM domain; they have already been reported to cause congenital profound hearing loss [31–33]. Therefore, four variants detected in this study were expected to cause profound hearing loss; however, they were associated with partial deafness with significant residual or even near-normal hearing at low frequencies.

The establishment of genotype-phenotype correlation is one of the fundament goals of genetics, as it enables personalized and timely management of diseases, leading to great contribution to precision medicine. Personalized and timely auditory rehabilitation is crucial in hearing loss because there is a critical time window for auditory development and degeneration. Therefore, an establishment of genotype-phenotype correlation is a meaningful issue in hearing loss. Sequentially, genotype-phenotype correlations of several genes and their pathogenic variants have been investigated widely. However, genotype-phenotype correlations have generally been based on the observation of phenotypes, rather than prudent analysis of pathogenic mechanism. Previously established genotype-phenotype correlations could be modified. Recently, several genes, including *MYO15A*, *CDH23,* and *PTPRQ,* have been reported to cause different types of hearing loss from those previously postulated in the literature [9, 31].

*MYO15A* is one of the genes with a well-documented genotype-phenotype correlation. It has been reported that hearing loss phenotype related to *MYO15A* is different according to the affected domain. Specifically, pathogenic variants in the N-terminal domain of *MYO15A* have been suggested to be associated with residual hearing [5–8], especially at low frequencies, while pathogenic variants in other domains resulted in congenital severe-to-profound hearing loss [1, 10–15]. With the progress of various auditory rehabilitation technologies, appropriate auditory rehabilitation tailored to the type of hearing loss should be implemented. A moderate hearing loss can be fully rehabilitated with hearing aids; however, cochlear implantation may be necessary in cases of severe hearing loss. Moreover, for subjects with overall profound hearing loss, while retaining significant residual hearing at low frequencies, electroacoustic stimulation (EAS) may be the best option. Therefore, a prediction of phenotypes in accordance with the affected domain in *MYO15A* has greatly contributed to personalized and timely auditory rehabilitation. Recently, these previously established genotype-phenotype correlations appear to require an updated modification. Some *MYO15A* pathogenic variants affecting the domains other than the N-terminal, have shown to cause moderate-to-severe hearing loss, not profound hearing loss [9]. We also found two families carrying *MYO15A* pathogenic variants, which were expected to cause congenital severe-to-profound hearing loss, but resulted in postlingual onset of progressive partial deafness with residual hearing at low frequencies. Indeed, SB224–437 carrying two *MYO15A* mutant alleles in this current study had a shallower angular insertion depth of 393°, by Flex 24 rather than by the longer Flex 28 electrode to minimize the shift in low frequency thresholds from this subject (Fig. 1b).

A milder phenotype may have been influenced by factors like milder pathogenic potential from hypomorphic alleles of *MYO15A*, genetic modifiers that reduce severity of hearing loss, or environmental factors. A recent progress in the genetic diagnosis technique can also contribute to the expansion of phenotypic spectrum of *MYO15* alterations. In the past, genetic hearing loss had been found in consanguineous families using a linkage analysis, especially homozygous mapping. Therefore, it is likely that the severe phenotypes caused by severely pathogenic homozygous pathogenic variants had been preferentially recruited for the genetic study. However, with the development of next generation sequencing, there has been an increase in the frequency of molecular diagnostic testing of small-to-mid sized, non-consanguineous families, leading to the emergence of many compound heterozygotes with varying degrees of pathogenicity. Consequently, it has become possible to discover various phenotypes from the existing genes. Further studies are needed about other potential factors affecting phenotypes, such as genetic modifiers or environmental factors.

## Conclusions

We discovered that four *MYO15A* pathogenic variants in the motor and FERM domains caused partial deafness with significant residual hearing at low frequencies. This is interesting since these pathogenic variants were previously thought to cause profound hearing loss, without any association with partial deafness. This result suggests that some *MYO15A* variants may cause adult-onset, progressive partial deafness. The phenotypic spectrum of DFNB3 should be extended to include such partial deafness.

## Additional files

Additional file 1: **Table S1.** List of known 129 deafness genes targeted in this study. (DOCX 46 kb)

Additional file 2: **Table S2.** Depth of coverage of customized panel sequencing. The table enumerates the mean coverage of the targeted regions from customized panel sequencing sample calculated by mpileup of samtools. (DOCX 206 kb)

Additional file 3: **Table S3.** Exome sequencing statistics. The table lists information of human genome reference (v19), customized panel and whole exome sequencing aligned reads from each patient estimated by CalculateHsMetrics of picard tool. (DOCX 29 kb)

## Abbreviations

1000G: 1000 Genomes Project
CNV: Copy number variation
EAS: Electroacoustic stimulation
ESP6500: Exome Sequencing Project 6500
ExAC: Exome Aggregation Consortium
GO-ESP: Grand Opportunity Exome Sequencing Project
KRGDB: Korean Reference Genome Database
MAF: Minor allele frequency
SNHL: Sensorineural hearing loss
SNP: Single nucleotide polymorphism
TES: Targeted exome sequencing
WES: Whole exome sequencing
