# Supplementary material for: Expansion of phenotypic spectrum of MYO15A pathogenic variants to include postlingual onset of progressive partial deafness
Source: BMC Med Genet. 2018 Feb 27;19:29. doi: 10.1186/s12881-018-0541-9 (PMC6389081; doi:10.1186/s12881-018-0541-9)
Supplement: Supplementary file 1 — Table S1. List of known 129 deafness genes targeted in this study. (DOCX 46 kb) [file 12881_2018_541_MOESM1_ESM.docx]

**Additional file 1: Table S1. List of known 129 deafness genes targeted in this study.**

|  | | | |
| --- | --- | --- | --- |
| **Symbol** | **Annotation** | **HGNC** | **OMIM** |
| ACTB | actin, beta | 132 | [607371] DYSTONIA, JUVENILE-ONSET |
| ACTG1 | actin, gamma 1 | 144 | [604717] DEAFNESS, AUTOSOMAL DOMINANT 20; DFNA20 |
| ATP6V1B1 | ATPase, H+ transporting, lysosomal 56/58kDa, V1 subunit B1 | 853 | [267300] RENAL TUBULAR ACIDOSIS, DISTAL, WITH PROGRESSIVE NERVE DEAFNESS |
| ATP6V1B2 | ATPase, H+ transporting, lysosomal 56/58kDa, V1 subunit B2 | 854 | #N/A |
| BCS1L | BCS1-like (S. cerevisiae) | 1020 | [603358] GRACILE SYNDROME [262000] BJORNSTAD SYNDROME; BJS [256000] LEIGH SYNDROME; LS [124000] MITOCHONDRIAL COMPLEX III DEFICIENCY |
| BSND | Bartter syndrome, infantile, with sensorineural deafness (Barttin) | 16512 | [602522] BARTTER SYNDROME, TYPE 4A |
| CATSPER2 | cation channel, sperm associated 2 | 18810 | [611102] DEAFNESS, SENSORINEURAL, AND MALE INFERTILITY |
| CCDC50 | coiled-coil domain containing 50 | 18111 | [607453] DEAFNESS, AUTOSOMAL DOMINANT 44; DFNA44 |
| CDH23 | cadherin-related 23 | 13733 | [601386] DEAFNESS, AUTOSOMAL RECESSIVE 12; DFNB12 [601067] USHER SYNDROME, TYPE ID; USH1D [276900] USHER SYNDROME, TYPE I; USH1 |
| CEACAM16 | carcinoembryonic antigen-related cell adhesion molecule 16 | 31948 | [614614] DEAFNESS, AUTOSOMAL DOMINANT 4B |
| CLDN14 | claudin 14 | 2035 | [614035] DEAFNESS, AUTOSOMAL RECESSIVE 29; DFNB29 |
| CLRN1 | clarin 1 | 12605 | [276902] [USHER SYNDROME, TYPE 3](http://www.ncbi.nlm.nih.gov/gtr/conditions/C1568248) |
| COCH | coagulation factor C homolog, cochlin (Limulus polyphemus) | 2180 | [601369] DEAFNESS, AUTOSOMAL DOMINANT 9; DFNA9 |
| COL11A2 | collagen, type XI, alpha 2 | 2187 | [614524] FIBROCHONDROGENESIS 2; FBCG2 [609706] DEAFNESS, AUTOSOMAL RECESSIVE 53; DFNB53 [601868] DEAFNESS, AUTOSOMAL DOMINANT 13; DFNA13 [277610] WEISSENBACHER-ZWEYMULLER SYNDROME; WZS [215150] OTOSPONDYLOMEGAEPIPHYSEAL DYSPLASIA; OSMED [184840] STICKLER SYNDROME, TYPE III; STL3 |
| COL9A2 | collagen, type IX, alpha 2 | 2218 | [614284] [STICKLER SYNDROME, TYPE 5](http://www.ncbi.nlm.nih.gov/gtr/conditions/CN116437) |
| COL9A3 | collagen, type IX, alpha 3 | 2219 | [603932] INTERVERTEBRAL DISC DISEASE; IDD [600969] EPIPHYSEAL DYSPLASIA, MULTIPLE, 3; EDM3 |
| CRYM | crystallin, mu | 2418 | #N/A |
| DFNA5 | deafness, autosomal dominant 5 | 2810 | [600994] DEAFNESS, AUTOSOMAL DOMINANT 5; DFNA5 |
| DFNB31 | deafness, autosomal recessive 31 | 16361 | [611383] USHER SYNDROME, TYPE IID; USH2D [607084] DEAFNESS, AUTOSOMAL RECESSIVE 31; DFNB31 |
| DFNB59 | deafness, autosomal recessive 59 | 29502 | [610220] DEAFNESS, AUTOSOMAL RECESSIVE 59; DFNB59 |
| DIAPH1 | diaphanous homolog 1 (Drosophila) | 2876 | [124900] DEAFNESS, AUTOSOMAL DOMINANT 1; DFNA1 |
| DSPP | dentin sialophosphoprotein | 3054 | [605594] DEAFNESS, AUTOSOMAL DOMINANT 39, WITH DENTINOGENESIS IMPERFECTA 1 [125500] DENTINOGENESIS IMPERFECTA, SHIELDS TYPE III [125490] DENTINOGENESIS IMPERFECTA 1; DGI1 [125420] DENTIN DYSPLASIA, TYPE II |
| ECE1 | endothelin converting enzyme 1 | 3146 | [613870] [HIRSCHSPRUNG DISEASE, CARDIAC DEFECTS, AND AUTONOMIC DYSFUNCTION](http://www.ncbi.nlm.nih.gov/gtr/conditions/C3151237) |
| EDNRA | [endothelin receptor type A](http://www.genenames.org/cgi-bin/gene_symbol_report?hgnc_id=3179) | 3179 | [157300] [MIGRAINE](http://www.ncbi.nlm.nih.gov/gtr/conditions/C0149931) |
| EDNRB | endothelin receptor type B | 3180 | [600501] [ABCD SYNDROME](http://www.ncbi.nlm.nih.gov/gtr/conditions/C1838099) |
| ERCC2 | excision repair cross-complementing rodent repair deficiency, complementation group 2 | 3434 | [610756] CEREBROOCULOFACIOSKELETAL SYNDROME 2; COFS2 [601675] TRICHOTHIODYSTROPHY, PHOTOSENSITIVE; TTDP [278730] XERODERMA PIGMENTOSUM, COMPLEMENTATION GROUP D; XPD |
| ERCC3 | excision repair cross-complementing rodent repair deficiency, complementation group 3 (xeroderma pigmentosum group B complementing) | 3435 | [610651] XERODERMA PIGMENTOSUM, COMPLEMENTATION GROUP B; XPB [601675] TRICHOTHIODYSTROPHY, PHOTOSENSITIVE; TTDP |
| ESPN | espin | 13281 | [609006] DEAFNESS, AUTOSOMAL RECESSIVE 36, WITH OR WITHOUT VESTIBULAR INVOLVEMENT |
| ESRRB | estrogen-related receptor beta | 3473 | [608565] DEAFNESS, AUTOSOMAL RECESSIVE 35; DFNB35 |
| EYA4 | eyes absent homolog 4 (Drosophila) | 3522 | [605362] CARDIOMYOPATHY, DILATED, 1J; CMD1J [601316] DEAFNESS, AUTOSOMAL DOMINANT 10; DFNA10 |
| FAS | Fas cell surface death receptor | 11920 | [601859] [AUTOIMMUNE LYMPHOPROLIFERATIVE SYNDROME](http://www.ncbi.nlm.nih.gov/gtr/conditions/C1328840) |
| FGF3 | fibroblast growth factor 3 | 3681 | [610706] DEAFNESS, CONGENITAL, WITH INNER EAR AGENESIS, MICROTIA, AND MICRODONTIA |
| FGFR3 | [fibroblast growth factor receptor 3](http://www.genenames.org/cgi-bin/gene_symbol_report?hgnc_id=3690) | 3690 | [610474] [CAMPTODACTYLY, TALL STATURE, AND HEARING LOSS SYNDROME](http://www.ncbi.nlm.nih.gov/gtr/conditions/C1864852) |
| FOXI1 | [forkhead box I1](http://www.genenames.org/cgi-bin/gene_symbol_report?hgnc_id=3815) | 3815 | [600791] [ENLARGED VESTIBULAR AQUEDUCT SYNDROME](http://www.ncbi.nlm.nih.gov/gtr/conditions/C1863752)  [274600] [PENDRED'S SYNDROME](http://www.ncbi.nlm.nih.gov/gtr/conditions/C0271829) |
| GATA3 | GATA binding protein 3 | 4172 | [146255] HYPOPARATHYROIDISM, SENSORINEURAL DEAFNESS, AND RENAL DISEASE; HDR |
| GIPC3 | [GIPC PDZ domain containing family, member 3](http://www.genenames.org/cgi-bin/gene_symbol_report?hgnc_id=18183) | 18183 | [601869] [DEAFNESS, AUTOSOMAL RECESSIVE 15](http://www.ncbi.nlm.nih.gov/gtr/conditions/C1866094) |
| GJA1 | gap junction protein, alpha 1, 43kDa | 4274 | [600309] ATRIOVENTRICULAR SEPTAL DEFECT 3; AVSD3 [257850] OCULODENTODIGITAL DYSPLASIA, AUTOSOMAL RECESSIVE [241550] HYPOPLASTIC LEFT HEART SYNDROME 1; HLHS1 [234100] HALLERMANN-STREIFF SYNDROME; HSS [186100] SYNDACTYLY, TYPE III [164200] OCULODENTODIGITAL DYSPLASIA; ODDD |
| GJB1 | gap junction protein, beta 1, 32kDa | 4283 | [302800] CHARCOT-MARIE-TOOTH DISEASE, X-LINKED DOMINANT, 1; CMTX1 [145900] HYPERTROPHIC NEUROPATHY OF DEJERINE-SOTTAS |
| GJB2 | gap junction protein, beta 2, 26kDa | 4284 | [602540] ICHTHYOSIS, HYSTRIX-LIKE, WITH DEAFNESS [601544] DEAFNESS, AUTOSOMAL DOMINANT 3A; DFNA3A [220290] DEAFNESS, AUTOSOMAL RECESSIVE 1A; DFNB1A [149200] KNUCKLE PADS, LEUKONYCHIA, AND SENSORINEURAL DEAFNESS [148350] KERATODERMA, PALMOPLANTAR, WITH DEAFNESS [148210] KERATITIS-ICHTHYOSIS-DEAFNESS SYNDROME, AUTOSOMAL DOMINANT [124500] DEAFNESS, CONGENITAL, WITH KERATOPACHYDERMIA AND CONSTRICTIONS OF |
| GJB3 | gap junction protein, beta 3, 31kDa | 4285 | [612644] DEAFNESS, AUTOSOMAL DOMINANT 2B; DFNA2B [603324] GAP JUNCTION PROTEIN, BETA-3; GJB3 [600101] DEAFNESS, AUTOSOMAL DOMINANT 2A; DFNA2A [220290] DEAFNESS, AUTOSOMAL RECESSIVE 1A; DFNB1A [133200] ERYTHROKERATODERMIA VARIABILIS ET PROGRESSIVA; EKVP |
| GJB4 | gap junction protein, beta 4, 30.3kDa | 4286 | [133200] ERYTHROKERATODERMIA VARIABILIS ET PROGRESSIVA; EKVP |
| GJB6 | gap junction protein, beta 6, 30kDa | 4288 | [612645] DEAFNESS, AUTOSOMAL RECESSIVE 1B; DFNB1B [612643] DEAFNESS, AUTOSOMAL DOMINANT 3B; DFNA3B [601544] DEAFNESS, AUTOSOMAL DOMINANT 3A; DFNA3A [220290] DEAFNESS, AUTOSOMAL RECESSIVE 1A; DFNB1A [129500] ECTODERMAL DYSPLASIA, HIDROTIC, AUTOSOMAL DOMINANT |
| GPR98 | [G protein-coupled receptor 98](http://www.genenames.org/cgi-bin/gene_symbol_report?hgnc_id=17416) | 17416 | [605472] [USHER SYNDROME, TYPE 2C](http://www.ncbi.nlm.nih.gov/gtr/conditions/C1854237) |
| GPSM2 | [G-protein signaling modulator 2](http://www.genenames.org/cgi-bin/gene_symbol_report?hgnc_id=29501) | 29501 | [604213] [CHUDLEY-MCCULLOUGH SYNDROME](http://www.ncbi.nlm.nih.gov/gtr/conditions/C1858695) |
| GRHL2 | grainyhead-like 2 (Drosophila) | 2799 | [608641] DEAFNESS, AUTOSOMAL DOMINANT 28; DFNA28 |
| GRXCR1 | [glutaredoxin, cysteine rich 1](http://www.genenames.org/cgi-bin/gene_symbol_report?hgnc_id=31673) | 31673 | [613285] [DEAFNESS, AUTOSOMAL RECESSIVE 25](http://www.ncbi.nlm.nih.gov/gtr/conditions/C1414017) |
| GSTP1 | glutathione S-transferase pi 1 | 4638 | #N/A |
| HAL | [histidine ammonia-lyase](http://www.genenames.org/cgi-bin/gene_symbol_report?hgnc_id=4806) | 4806 | [235800] [HISTIDINEMIA](http://www.ncbi.nlm.nih.gov/gtr/conditions/C0220992) |
| HGF | [hepatocyte growth factor (hepapoietin A; scatter factor)](http://www.genenames.org/cgi-bin/gene_symbol_report?hgnc_id=4893) | 4893 | [608265] [DEAFNESS, AUTOSOMAL RECESSIVE 39](http://www.ncbi.nlm.nih.gov/gtr/conditions/C1842342) |
| ILDR1 | [immunoglobulin-like domain containing receptor 1](http://www.genenames.org/cgi-bin/gene_symbol_report?hgnc_id=28741) | 28741 | [609646] [DEAFNESS, AUTOSOMAL RECESSIVE 42](http://www.ncbi.nlm.nih.gov/gtr/conditions/C1864818) |
| JAG1 | jagged 1 | 6188 | [601920] JAGGED 1; JAG1 [187500] TETRALOGY OF FALLOT; TOF [118450] ALAGILLE SYNDROME 1; ALGS1 |
| KCNE1 | potassium voltage-gated channel, Isk-related family, member 1 | 6240 | [613695] LONG QT SYNDROME 5; LQT5 [612347] JERVELL AND LANGE-NIELSEN SYNDROME 2; JLNS2 [220400] JERVELL AND LANGE-NIELSEN SYNDROME 1; JLNS1 |
| KCNJ10 | potassium inwardly-rectifying channel, subfamily J, member 10 | 6256 | [612780] SEIZURES, SENSORINEURAL DEAFNESS, ATAXIA, MENTAL RETARDATION, AND [600791] ENLARGED VESTIBULAR AQUEDUCT; EVA |
| KCNQ1 | potassium voltage-gated channel, KQT-like subfamily, member 1 | 6294 | [220400] JERVELL AND LANGE-NIELSEN SYNDROME |
| KCNQ4 | potassium voltage-gated channel, KQT-like subfamily, member 4 | 6298 | [600101] DEAFNESS, AUTOSOMAL DOMINANT 2A; DFNA2A |
| KIAA1199 | KIAA1199 | #N/A | #N/A |
| LHFPL5 | lipoma HMGIC fusion partner-like 5 | 21253 | [610265] DEAFNESS, AUTOSOMAL RECESSIVE 67; DFNB67 |
| LHX3 | LIM homeobox 3 | 6595 | [262600] PITUITARY HORMONE DEFICIENCY, COMBINED, 2; CPHD2 [221750] PITUITARY HORMONE DEFICIENCY, COMBINED, 3; CPHD3 |
| LOXHD1 | [lipoxygenase homology domains 1](http://www.genenames.org/cgi-bin/gene_symbol_report?hgnc_id=26521) | 26521 | [613079] [DEAFNESS, AUTOSOMAL RECESSIVE 77](http://www.ncbi.nlm.nih.gov/gtr/conditions/C2746083) |
| LRTOMT | leucine rich transmembrane and 0-methyltransferase domain containing | 25033 | [611451] DEAFNESS, AUTOSOMAL RECESSIVE 63; DFNB63 |
| MARVELD2 | MARVEL domain containing 2 | 26401 | [610153] DEAFNESS, AUTOSOMAL RECESSIVE 49; DFNB49 |
| mir182 | [microRNA 182](http://www.genenames.org/cgi-bin/gene_symbol_report?hgnc_id=31553) | 31553 | #N/A |
| mir183 | [microRNA 183](http://www.genenames.org/cgi-bin/gene_symbol_report?hgnc_id=31554) | 31554 | #N/A |
| mir96 | [microRNA 96](http://www.genenames.org/cgi-bin/gene_symbol_report?hgnc_id=31648) | 31648 | [613074] [DEAFNESS, AUTOSOMAL DOMINANT 50](http://www.ncbi.nlm.nih.gov/gtr/conditions/CN035509) |
| MITF | [microphthalmia-associated transcription factor](http://www.genenames.org/cgi-bin/gene_symbol_report?hgnc_id=7105) | 7105 | [103470] [ALBINISM, OCULAR, WITH SENSORINEURAL DEAFNESS](http://www.ncbi.nlm.nih.gov/gtr/conditions/C1863198) |
| MSRB3 | [methionine sulfoxide reductase B3](http://www.genenames.org/cgi-bin/gene_symbol_report?hgnc_id=27375) | 27375 | [613718] [DEAFNESS, AUTOSOMAL RECESSIVE 74](http://www.ncbi.nlm.nih.gov/gtr/conditions/C2239351) |
| MTAP | methylthioadenosine phosphorylase | 7413 | #N/A |
| MT-TD | [mitochondrially encoded tRNA aspartic acid](http://www.genenames.org/cgi-bin/gene_symbol_report?hgnc_id=7478) | 7478 | #N/A |
| MT-TH | [mitochondrially encoded tRNA histidine](http://www.genenames.org/cgi-bin/gene_symbol_report?hgnc_id=7487) | 7487 | #N/A |
| MT-TI | mitochondrially encoded tRNA isoleucine | 7488 | #N/A |
| MT-TK | [mitochondrially encoded tRNA lysine](http://www.genenames.org/cgi-bin/gene_symbol_report?hgnc_id=7489) | 7489 | [540000] [JUVENILE MYOPATHY, ENCEPHALOPATHY, LACTIC ACIDOSIS AND STROKE](http://www.ncbi.nlm.nih.gov/gtr/conditions/C0162671) |
| MT-TL1 | [mitochondrially encoded tRNA leucine 1 (UUA/G)](http://www.genenames.org/cgi-bin/gene_symbol_report?hgnc_id=7490) | 7490 | [540000] [JUVENILE MYOPATHY, ENCEPHALOPATHY, LACTIC ACIDOSIS AND STROKE](http://www.ncbi.nlm.nih.gov/gtr/conditions/C0162671) |
| MT-TL2 | [mitochondrially encoded tRNA leucine 2 (CUN)](http://www.genenames.org/cgi-bin/gene_symbol_report?hgnc_id=7491) | 7491 | #N/A |
| MT-TM | [mitochondrially encoded tRNA methionine](http://www.genenames.org/cgi-bin/gene_symbol_report?hgnc_id=7492) | 7492 | #N/A |
| MT-TQ | [mitochondrially encoded tRNA glutamine](http://www.genenames.org/cgi-bin/gene_symbol_report?hgnc_id=7495) | 7495 | [540000] [JUVENILE MYOPATHY, ENCEPHALOPATHY, LACTIC ACIDOSIS AND STROKE](http://www.ncbi.nlm.nih.gov/gtr/conditions/C0162671) |
| MT-TS1 | [mitochondrially encoded tRNA serine 1 (UCN)](http://www.genenames.org/cgi-bin/gene_symbol_report?hgnc_id=7497) | 7497 | [500008] [DEAFNESS, NONSYNDROMIC SENSORINEURAL, MITOCHONDRIAL](http://www.ncbi.nlm.nih.gov/gtr/conditions/C3151897)  [580000] [AMINOGLYCOSIDE-INDUCED DEAFNESS](http://www.ncbi.nlm.nih.gov/gtr/conditions/C1838854)  [540000] [JUVENILE MYOPATHY, ENCEPHALOPATHY, LACTIC ACIDOSIS AND STROKE](http://www.ncbi.nlm.nih.gov/gtr/conditions/C0162671) |
| MT-TS2 | [mitochondrially encoded tRNA serine 2 (AGU/C)](http://www.genenames.org/cgi-bin/gene_symbol_report?hgnc_id=7498) | 7498 | [540000] [JUVENILE MYOPATHY, ENCEPHALOPATHY, LACTIC ACIDOSIS AND STROKE](http://www.ncbi.nlm.nih.gov/gtr/conditions/C0162671) |
| MYH14 | myosin, heavy chain 14, non-muscle | 23212 | [614369] PERIPHERAL NEUROPATHY, MYOPATHY, HOARSENESS, AND HEARING LOSS; PNMHH [600652] DEAFNESS, AUTOSOMAL DOMINANT 4; DFNA4 |
| MYH9 | myosin, heavy chain 9, non-muscle | 7579 | [605249] SEBASTIAN SYNDROME; SBS [603622] DEAFNESS, AUTOSOMAL DOMINANT 17; DFNA17 [600208] MACROTHROMBOCYTOPENIA AND PROGRESSIVE SENSORINEURAL DEAFNESS [155100] MAY-HEGGLIN ANOMALY; MHA [153650] EPSTEIN SYNDROME, [153640] FECHTNER SYNDROME; FTNS |
| MYO15A | myosin XVA | 7594 | [600316] DEAFNESS, AUTOSOMAL RECESSIVE 3; DFNB3 |
| MYO1A | myosin IA | 7595 | [607841] DEAFNESS, AUTOSOMAL DOMINANT 48; DFNA48 |
| MYO1C | myosin IC | 7597 | #N/A |
| MYO1F | myosin IF | 7600 | #N/A |
| MYO3A | myosin IIIA | 7601 | [607101] DEAFNESS, AUTOSOMAL RECESSIVE 30; DFNB30 |
| MYO6 | myosin VI | 7605 | [607821] DEAFNESS, AUTOSOMAL RECESSIVE 37; DFNB37 [606346] DEAFNESS, AUTOSOMAL DOMINANT 22; DFNA22 |
| MYO7A | myosin VIIA | 7606 | [601317] DEAFNESS, AUTOSOMAL DOMINANT 11; DFNA11 [600060] DEAFNESS, AUTOSOMAL RECESSIVE 2; DFNB2 [276900] USHER SYNDROME, TYPE I; USH1 |
| NDP | Norrie disease (pseudoglioma) | 7678 | [310600] [ATROPHIA BULBORUM HEREDITARIA](http://www.ncbi.nlm.nih.gov/gtr/conditions/C0266526)  [305390] [X-LINKED FAMILIAL EXUDATIVE VITREORETINOPATHY](http://www.ncbi.nlm.nih.gov/gtr/conditions/C1844579) |
| NR2F1 | nuclear receptor subfamily 2, group F, member 1 | 7975 | #N/A |
| OTOA | otoancorin | 16378 | [607039] DEAFNESS, AUTOSOMAL RECESSIVE 22; DFNB22 |
| OTOF | otoferlin | 8515 | [601071] DEAFNESS, AUTOSOMAL RECESSIVE 9; DFNB9 |
| OTOR | otoraplin | 8517 | #N/A |
| P2RX2 | [purinergic receptor P2X, ligand-gated ion channel, 2](http://www.genenames.org/cgi-bin/gene_symbol_report?hgnc_id=15459) | 15459 | [608224] [DEAFNESS, AUTOSOMAL DOMINANT 41](http://www.ncbi.nlm.nih.gov/gtr/conditions/C1842371) |
| PAX3 | paired box 3 | 8617 | [268220] RHABDOMYOSARCOMA 2; RMS2 [193500] WAARDENBURG SYNDROME, TYPE 1; WS1 [148820] WAARDENBURG SYNDROME, TYPE 3; WS3 [122880] CRANIOFACIAL-DEAFNESS-HAND SYNDROME; CDHS |
| PCDH15 | protocadherin-related 15 | 14674 | [609533] DEAFNESS, AUTOSOMAL RECESSIVE 23; DFNB23 [602083] USHER SYNDROME, TYPE IF; USH1F [601067] USHER SYNDROME, TYPE ID; USH1D [276900] USHER SYNDROME, TYPE I; USH1 |
| PDZD7 | PDZ domain containing 7 | 26257 | [605472] USHER SYNDROME, TYPE IIC; USH2C [276901] USHER SYNDROME, TYPE IIA; USH2A |
| PMP22 | peripheral myelin protein 22 | 9118 | [180800] ROUSSY-LEVY HEREDITARY AREFLEXIC DYSTASIA [162500] NEUROPATHY, HEREDITARY, WITH LIABILITY TO PRESSURE PALSIES; HNPP [145900] HYPERTROPHIC NEUROPATHY OF DEJERINE-SOTTAS [139393] GUILLAIN-BARRE SYNDROME, FAMILIAL; GBS [118300] CHARCOT-MARIE-TOOTH DISEASE AND DEAFNESS [118220] CHARCOT-MARIE-TOOTH DISEASE, DEMYELINATING, TYPE 1A; CMT1A |
| POU3F4 | [POU class 3 homeobox 4](http://www.genenames.org/cgi-bin/gene_symbol_report?hgnc_id=9217) | 9217 | [304400] [DEAFNESS, X-LINKED 2](http://www.ncbi.nlm.nih.gov/gtr/conditions/C1844678) |
| POU4F3 | POU class 4 homeobox 3 | 9220 | [602459] DEAFNESS, AUTOSOMAL DOMINANT 15; DFNA15 |
| PRPS1 | [phosphoribosyl pyrophosphate synthetase 1](http://www.genenames.org/cgi-bin/gene_symbol_report?hgnc_id=9462) | 9462 | [30185] [ARTS SYNDROME](http://www.ncbi.nlm.nih.gov/gtr/conditions/C0796028)  [311070] [CHARCOT-MARIE-TOOTH DISEASE, X-LINKED RECESSIVE, TYPE 5](http://www.ncbi.nlm.nih.gov/gtr/conditions/C1839566)  [300661] [PHOSPHORIBOSYLPYROPHOSPHATE SYNTHETASE SUPERACTIVITY](http://www.ncbi.nlm.nih.gov/gtr/conditions/C1970827) |
| PTPRQ | [protein tyrosine phosphatase, receptor type, Q](http://www.genenames.org/cgi-bin/gene_symbol_report?hgnc_id=9679) | 9679 | [613391] [DEAFNESS, AUTOSOMAL RECESSIVE 84](http://www.ncbi.nlm.nih.gov/gtr/conditions/C3150654) |
| RDX | radixin | 9944 | [611022] DEAFNESS, AUTOSOMAL RECESSIVE 24; DFNB24 |
| SERPINB6 | [serpin peptidase inhibitor, clade B (ovalbumin), member 6](http://www.genenames.org/cgi-bin/gene_symbol_report?hgnc_id=8950) | 8950 | [613453] [DEAFNESS, AUTOSOMAL RECESSIVE 91](http://www.ncbi.nlm.nih.gov/gtr/conditions/C3150704) |
| SIX1 | [SIX homeobox 1](http://www.genenames.org/cgi-bin/gene_symbol_report?hgnc_id=10887) | 10887 | [605192] [DEAFNESS, AUTOSOMAL DOMINANT 23](http://www.ncbi.nlm.nih.gov/gtr/conditions/C1854594)  [608389] [BRANCHIOOTIC SYNDROME 3](http://www.ncbi.nlm.nih.gov/gtr/conditions/C1842124)  [113650] [MELNICK-FRASER SYNDROME](http://www.ncbi.nlm.nih.gov/gtr/conditions/C0265234) |
| SIX5 | [SIX homeobox 5](http://www.genenames.org/cgi-bin/gene_symbol_report?hgnc_id=10891) | 10891 | [610896] [BRANCHIOOTORENAL SYNDROME 2](http://www.ncbi.nlm.nih.gov/gtr/conditions/C1970479) |
| SLC17A8 | solute carrier family 17 (sodium-dependent inorganic phosphate cotransporter), member 8 | 20151 | [605583] DEAFNESS, AUTOSOMAL DOMINANT 25; DFNA25 |
| SLC26A4 | solute carrier family 26, member 4 | 8818 | [600791] ENLARGED VESTIBULAR AQUEDUCT; EVA [274600] PENDRED SYNDROME; PDS |
| SLC26A5 | solute carrier family 26, member 5 (prestin) | 9359 | [613865] DEAFNESS, AUTOSOMAL RECESSIVE 61; DFNB61 |
| SLC4A11 | solute carrier family 4, sodium borate transporter, member 11 | 16438 | [613268] CORNEAL DYSTROPHY, FUCHS ENDOTHELIAL, 4; FECD4 [217700] CORNEAL ENDOTHELIAL DYSTROPHY 2, AUTOSOMAL RECESSIVE; CHED2 [217400] CORNEAL DYSTROPHY AND PERCEPTIVE DEAFNESS |
| SMPX | [small muscle protein, X-linked](http://www.genenames.org/cgi-bin/gene_symbol_report?hgnc_id=11122) | 11122 | #N/A |
| SNAI2 | [snail family zinc finger 2](http://www.genenames.org/cgi-bin/gene_symbol_report?hgnc_id=11094) | 11094 | [608890] [WAARDENBURG SYNDROME TYPE 2D](http://www.ncbi.nlm.nih.gov/gtr/conditions/C1837203) |
| SOX2 | SRY (sex determining region Y)-box 2 | 11195 | [206900] MICROPHTHALMIA, SYNDROMIC 3; MCOPS3 |
| SPINK5 | serine peptidase inhibitor, Kazal type 5 | 15464 | [256500] NETHERTON SYNDROME; NETH [147050] IGE RESPONSIVENESS, ATOPIC; IGER |
| STRC | stereocilin | 16035 | [611102] DEAFNESS, SENSORINEURAL, AND MALE INFERTILITY [603720] DEAFNESS, AUTOSOMAL RECESSIVE 16; DFNB16 |
| TBL1X | transducin (beta)-like 1X-linked | 11585 | #N/A |
| TCF21 | transcription factor 21 | 11632 | #N/A |
| TECTA | tectorin alpha | 11720 | [603629] DEAFNESS, AUTOSOMAL RECESSIVE 21; DFNB21 [601543] DEAFNESS, AUTOSOMAL DOMINANT 12; DFNA12 |
| TFCP2 | [transcription factor CP2](http://www.genenames.org/cgi-bin/gene_symbol_report?hgnc_id=11748) | 11748 | #N/A |
| TIMM8A | translocase of inner mitochondrial membrane 8 homolog A (yeast) | 11817 | [311150] OPTICOACOUSTIC NERVE ATROPHY WITH DEMENTIA [304700] MOHR-TRANEBJAERG SYNDROME; MTS |
| TJP2 | [tight junction protein 2](http://www.genenames.org/cgi-bin/gene_symbol_report?hgnc_id=11828) | 11828 | [607748] [HYPERCHOLANEMIA, FAMILIAL](http://www.ncbi.nlm.nih.gov/gtr/conditions/C1843139) |
| TMC1 | transmembrane channel-like 1 | 16513 | [606705] DEAFNESS, AUTOSOMAL DOMINANT 36; DFNA36 [600974] DEAFNESS, AUTOSOMAL RECESSIVE 7; DFNB7 |
| TMIE | transmembrane inner ear | 30800 | [600971] DEAFNESS, AUTOSOMAL RECESSIVE 6; DFNB6 |
| TMPRSS3 | transmembrane protease, serine 3 | 11877 | [605316] DEAFNESS, AUTOSOMAL RECESSIVE 10; DFNB10 [601072] DEAFNESS, CHILDHOOD-ONSET NEUROSENSORY, AUTOSOMAL RECESSIVE 8; DFNB8 |
| TMPRSS5 | transmembrane protease, serine 5 | 14908 | #N/A |
| TPRN | [taperin](http://www.genenames.org/cgi-bin/gene_symbol_report?hgnc_id=26894) | 26894 | [613307] [DEAFNESS, AUTOSOMAL RECESSIVE 79](http://www.ncbi.nlm.nih.gov/gtr/conditions/C2750082) |
| TRIOBP | TRIO and F-actin binding protein | 17009 | [609823] DEAFNESS, AUTOSOMAL RECESSIVE 28; DFNB28 |
| USH1C | Usher syndrome 1C (autosomal recessive, severe) | 12597 | [602092] DEAFNESS, AUTOSOMAL RECESSIVE 18; DFNB18 [276904] USHER SYNDROME, TYPE IC; USH1C [276900] USHER SYNDROME, TYPE I; USH1 |
| USH1G | [Usher syndrome 1G (autosomal recessive)](http://www.genenames.org/cgi-bin/gene_symbol_report?hgnc_id=16356) | 16356 | [606943] [USHER SYNDROME, TYPE 1G](http://www.ncbi.nlm.nih.gov/gtr/conditions/C1847089) |
| USH2A | [Usher syndrome 2A (autosomal recessive, mild)](http://www.genenames.org/cgi-bin/gene_symbol_report?hgnc_id=12601) | 12601 | [276901] [USHER SYNDROME, TYPE 2A](http://www.ncbi.nlm.nih.gov/gtr/conditions/C1848634) |
| WFS1 | Wolfram syndrome 1 (wolframin) | 12762 | [614296] WOLFRAM-LIKE SYNDROME, AUTOSOMAL DOMINANT; WFSL [600965] DEAFNESS, AUTOSOMAL DOMINANT 6; DFNA6 [222300] WOLFRAM SYNDROME 1; WFS1 [125853] DIABETES MELLITUS, NONINSULIN-DEPENDENT; NIDDM |
