# Supplementary material for: Expansion of phenotypic spectrum of MYO15A pathogenic variants to include postlingual onset of progressive partial deafness
Source: BMC Med Genet. 2018 Feb 27;19:29. doi: 10.1186/s12881-018-0541-9 (PMC6389081; doi:10.1186/s12881-018-0541-9)
Supplement: Supplementary file 2 — Table S2. Depth of coverage of customized panel sequencing. The table enumerates the mean coverage of the targeted regions from customized panel sequencing sample calculated by mpileup of samtools. (DOCX 206 kb) [file 12881_2018_541_MOESM2_ESM.docx]

**Additional file 2: Table S2. Depth of coverage of customized panel sequencing.** The table enumerates the mean coverage of the targeted regions from customized panel sequencing sample calculated by mpileup of samtools.

| Chrom | Start | End | Length | Name | SB246-482-index46 |
| --- | --- | --- | --- | --- | --- |
| chrM | 626 | 1615 | 990 | MIR1978 | 1945.69 |
| chrM | 3229 | 3304 | 76 | MT-TL1 | 4071.41 |
| chrM | 4262 | 4469 | 208 | MT-TI\|MT-TQ\|MT-TM | 3147.29 |
| chrM | 7445 | 7514 | 70 | MT-TS1 | 2292.97 |
| chrM | 7517 | 7585 | 69 | MT-TD | 3632.22 |
| chrM | 8294 | 8364 | 71 | MT-TK | 1783.06 |
| chrM | 12137 | 12336 | 200 | MT-TH\|MT-TS2\|MT-TL2 | 4151.03 |
| chrM | 14673 | 14742 | 70 | MIR1974 | 4067.21 |
| chr1 | 6485016 | 6485309 | 294 | *ESPN* | 36.23 |
| chr1 | 6488286 | 6488479 | 194 | *ESPN* | 346.67 |
| chr1 | 6500314 | 6500500 | 187 | *ESPN* | 262.36 |
| chr1 | 6500686 | 6500868 | 183 | *ESPN* | 137.99 |
| chr1 | 6500994 | 6501125 | 132 | *ESPN* | 104.33 |
| chr1 | 6504541 | 6504742 | 202 | *ESPN* | 449.15 |
| chr1 | 6505449 | 6505834 | 386 | *ESPN* | 50.84 |
| chr1 | 6505858 | 6506250 | 393 | *ESPN* | 100.11 |
| chr1 | 6508701 | 6509151 | 451 | *ESPN* | 63.92 |
| chr1 | 6511663 | 6511808 | 146 | *ESPN* | 235.45 |
| chr1 | 6511893 | 6512156 | 264 | *ESPN* | 274.28 |
| chr1 | 6517234 | 6517333 | 100 | *ESPN* | 338.09 |
| chr1 | 6517377 | 6517476 | 100 | *ESPN* | 353.34 |
| chr1 | 6520059 | 6520206 | 148 | *ESPN* | 345.65 |
| chr1 | 21546447 | 21546624 | 178 | *ECE1* | 214.89 |
| chr1 | 21548239 | 21548335 | 97 | *ECE1* | 97.78 |
| chr1 | 21551742 | 21551933 | 192 | *ECE1* | 165.71 |
| chr1 | 21553651 | 21553719 | 69 | *ECE1* | 81 |
| chr1 | 21554423 | 21554534 | 112 | *ECE1* | 146.68 |
| chr1 | 21560050 | 21560154 | 105 | *ECE1* | 118.59 |
| chr1 | 21562342 | 21562420 | 79 | *ECE1* | 89.28 |
| chr1 | 21563238 | 21563337 | 100 | *ECE1* | 110.08 |
| chr1 | 21564626 | 21564737 | 112 | *ECE1* | 152.72 |
| chr1 | 21571481 | 21571596 | 116 | *ECE1* | 165.41 |
| chr1 | 21573713 | 21573856 | 144 | *ECE1* | 171.31 |
| chr1 | 21582439 | 21582631 | 193 | *ECE1* | 155.79 |
| chr1 | 21584017 | 21584083 | 67 | *ECE1* | 49.04 |
| chr1 | 21585185 | 21585332 | 148 | *ECE1* | 129.2 |
| chr1 | 21586763 | 21586885 | 123 | *ECE1* | 93.32 |
| chr1 | 21599191 | 21599404 | 214 | *ECE1* | 151.73 |
| chr1 | 21605683 | 21605825 | 143 | *ECE1* | 146.42 |
| chr1 | 21616562 | 21616691 | 130 | *ECE1* | 53.67 |
| chr1 | 35226856 | 35227656 | 801 | *GJB4* | 361.47 |
| chr1 | 35250364 | 35251176 | 813 | *GJB3* | 481.34 |
| chr1 | 40766853 | 40767053 | 201 | *COL9A2* | 275.8 |
| chr1 | 40767483 | 40767561 | 79 | *COL9A2* | 238.05 |
| chr1 | 40768292 | 40768481 | 190 | *COL9A2* | 157.55 |
| chr1 | 40768802 | 40768857 | 56 | *COL9A2* | 229.55 |
| chr1 | 40769201 | 40769348 | 148 | *COL9A2* | 314.09 |
| chr1 | 40769478 | 40769511 | 34 | *COL9A2* | 232.62 |
| chr1 | 40769599 | 40769644 | 46 | *COL9A2* | 372.8 |
| chr1 | 40769735 | 40769771 | 37 | *COL9A2* | 358.84 |
| chr1 | 40769991 | 40770063 | 73 | *COL9A2* | 195.71 |
| chr1 | 40770147 | 40770201 | 55 | *COL9A2* | 336.4 |
| chr1 | 40770465 | 40770519 | 55 | *COL9A2* | 126 |
| chr1 | 40771386 | 40771440 | 55 | *COL9A2* | 233.05 |
| chr1 | 40771814 | 40771859 | 46 | *COL9A2* | 267.72 |
| chr1 | 40773117 | 40773171 | 55 | *COL9A2* | 247.65 |
| chr1 | 40773374 | 40773428 | 55 | *COL9A2* | 318.73 |
| chr1 | 40773863 | 40773917 | 55 | *COL9A2* | 291.89 |
| chr1 | 40775609 | 40775663 | 55 | *COL9A2* | 405.15 |
| chr1 | 40775782 | 40775836 | 55 | *COL9A2* | 466.36 |
| chr1 | 40775935 | 40775989 | 55 | *COL9A2* | 358.13 |
| chr1 | 40776385 | 40776439 | 55 | *COL9A2* | 295.73 |
| chr1 | 40776764 | 40776818 | 55 | *COL9A2* | 308.35 |
| chr1 | 40776901 | 40776958 | 58 | *COL9A2* | 240.9 |
| chr1 | 40777171 | 40777219 | 49 | *COL9A2* | 148.86 |
| chr1 | 40777333 | 40777387 | 55 | *COL9A2* | 222.58 |
| chr1 | 40777730 | 40777784 | 55 | *COL9A2* | 229.62 |
| chr1 | 40778127 | 40778151 | 25 | *COL9A2* | 274.24 |
| chr1 | 40778245 | 40778281 | 37 | *COL9A2* | 441.05 |
| chr1 | 40778402 | 40778456 | 55 | *COL9A2* | 358.49 |
| chr1 | 40779876 | 40779939 | 64 | *COL9A2* | 462.53 |
| chr1 | 40780023 | 40780059 | 37 | *COL9A2* | 338.03 |
| chr1 | 40781261 | 40781336 | 76 | *COL9A2* | 273.57 |
| chr1 | 40782794 | 40782869 | 76 | *COL9A2* | 290.61 |
| chr1 | 41249919 | 41250300 | 382 | *KCNQ4* | 108.22 |
| chr1 | 41282932 | 41283032 | 101 | *KCNQ4* | 219.44 |
| chr1 | 41283836 | 41283962 | 127 | *KCNQ4* | 144.98 |
| chr1 | 41284177 | 41284352 | 176 | *KCNQ4* | 118.98 |
| chr1 | 41285019 | 41285144 | 126 | *KCNQ4* | 208.6 |
| chr1 | 41285547 | 41285657 | 111 | *KCNQ4* | 187.76 |
| chr1 | 41285835 | 41285934 | 100 | *KCNQ4* | 179.3 |
| chr1 | 41287980 | 41288080 | 101 | *KCNQ4* | 149.85 |
| chr1 | 41289769 | 41289930 | 162 | *KCNQ4* | 49.17 |
| chr1 | 41296756 | 41296976 | 221 | *KCNQ4* | 144.28 |
| chr1 | 41298686 | 41298785 | 100 | *KCNQ4* | 74.57 |
| chr1 | 41300639 | 41300770 | 132 | *KCNQ4* | 102.05 |
| chr1 | 41303337 | 41303466 | 130 | *KCNQ4* | 80.4 |
| chr1 | 41303983 | 41304195 | 213 | *KCNQ4* | 233.71 |
| chr1 | 55464860 | 55465036 | 177 | *BSND* | 311.01 |
| chr1 | 55470692 | 55470792 | 101 | *BSND* | 256.19 |
| chr1 | 55472670 | 55472945 | 276 | *BSND* | 358.48 |
| chr1 | 55473887 | 55474301 | 415 | *BSND* | 356.2 |
| chr1 | 109428144 | 109428200 | 57 | *GPSM2* | 278.42 |
| chr1 | 109439485 | 109439707 | 223 | *GPSM2* | 256.45 |
| chr1 | 109440113 | 109440249 | 137 | *GPSM2* | 184.61 |
| chr1 | 109440580 | 109440723 | 144 | *GPSM2* | 219.56 |
| chr1 | 109441263 | 109441387 | 125 | *GPSM2* | 358.1 |
| chr1 | 109441500 | 109441616 | 117 | *GPSM2* | 264.81 |
| chr1 | 109444411 | 109444567 | 157 | *GPSM2* | 319.56 |
| chr1 | 109445747 | 109445856 | 110 | *GPSM2* | 311.52 |
| chr1 | 109446746 | 109446876 | 131 | *GPSM2* | 212.93 |
| chr1 | 109456959 | 109457030 | 72 | *GPSM2* | 126.6 |
| chr1 | 109461234 | 109461411 | 178 | *GPSM2* | 289.63 |
| chr1 | 109465038 | 109465198 | 161 | *GPSM2* | 300.28 |
| chr1 | 109466621 | 109466836 | 216 | *GPSM2* | 332.59 |
| chr1 | 109472322 | 109472562 | 241 | *GPSM2* | 291.69 |
| chr1 | 160011183 | 160012322 | 1140 | *KCNJ10* | 282.34 |
| chr1 | 215799122 | 215799212 | 91 | *USH2A* | 211.79 |
| chr1 | 215802155 | 215802377 | 223 | *USH2A* | 304.78 |
| chr1 | 215807800 | 215808045 | 246 | *USH2A* | 260.78 |
| chr1 | 215812496 | 215812580 | 85 | *USH2A* | 39.69 |
| chr1 | 215813899 | 215814076 | 178 | *USH2A* | 65.34 |
| chr1 | 215820863 | 215821072 | 210 | *USH2A* | 25.71 |
| chr1 | 215821869 | 215822108 | 240 | *USH2A* | 19.83 |
| chr1 | 215823933 | 215824143 | 211 | *USH2A* | 131.69 |
| chr1 | 215844313 | 215844635 | 323 | *USH2A* | 178.88 |
| chr1 | 215847441 | 215848958 | 1518 | *USH2A* | 68.37 |
| chr1 | 215853490 | 215853718 | 229 | *USH2A* | 235.68 |
| chr1 | 215901371 | 215901726 | 356 | *USH2A* | 362.19 |
| chr1 | 215914716 | 215914879 | 164 | *USH2A* | 243.4 |
| chr1 | 215916518 | 215916677 | 160 | *USH2A* | 194.92 |
| chr1 | 215931936 | 215932094 | 159 | *USH2A* | 109.24 |
| chr1 | 215933001 | 215933185 | 185 | *USH2A* | 145.4 |
| chr1 | 215940022 | 215940130 | 109 | *USH2A* | 351.83 |
| chr1 | 215953184 | 215953383 | 200 | *USH2A* | 412.67 |
| chr1 | 215955383 | 215955538 | 156 | *USH2A* | 186.47 |
| chr1 | 215956079 | 215956277 | 199 | *USH2A* | 147.71 |
| chr1 | 215960011 | 215960216 | 206 | *USH2A* | 337.62 |
| chr1 | 215963400 | 215963624 | 225 | *USH2A* | 241.77 |
| chr1 | 215972248 | 215972467 | 220 | *USH2A* | 310.33 |
| chr1 | 215987077 | 215987246 | 170 | *USH2A* | 284.55 |
| chr1 | 215990338 | 215990537 | 200 | *USH2A* | 241.22 |
| chr1 | 216011332 | 216011445 | 114 | *USH2A* | 360.16 |
| chr1 | 216017635 | 216017838 | 204 | *USH2A* | 164.47 |
| chr1 | 216019165 | 216019375 | 211 | *USH2A* | 179.43 |
| chr1 | 216040348 | 216040512 | 165 | *USH2A* | 309.18 |
| chr1 | 216051099 | 216051222 | 124 | *USH2A* | 164.02 |
| chr1 | 216052105 | 216052440 | 336 | *USH2A* | 225.76 |
| chr1 | 216061767 | 216062396 | 630 | *USH2A* | 279.35 |
| chr1 | 216073416 | 216073559 | 144 | *USH2A* | 199.56 |
| chr1 | 216074096 | 216074247 | 152 | *USH2A* | 234.12 |
| chr1 | 216107957 | 216108137 | 181 | *USH2A* | 166.14 |
| chr1 | 216138658 | 216138821 | 164 | *USH2A* | 255.91 |
| chr1 | 216143966 | 216144118 | 153 | *USH2A* | 313.05 |
| chr1 | 216166361 | 216166509 | 149 | *USH2A* | 320.54 |
| chr1 | 216172228 | 216172400 | 173 | *USH2A* | 42.11 |
| chr1 | 216173744 | 216173904 | 161 | *USH2A* | 56.94 |
| chr1 | 216219772 | 216219934 | 163 | *USH2A* | 279.2 |
| chr1 | 216221875 | 216221989 | 115 | *USH2A* | 318.61 |
| chr1 | 216243442 | 216243634 | 193 | *USH2A* | 278.19 |
| chr1 | 216246230 | 216246311 | 82 | *USH2A* | 291.37 |
| chr1 | 216246438 | 216246642 | 205 | *USH2A* | 281.64 |
| chr1 | 216251430 | 216251704 | 275 | *USH2A* | 469.25 |
| chr1 | 216256797 | 216256928 | 132 | *USH2A* | 58.79 |
| chr1 | 216258039 | 216258219 | 181 | *USH2A* | 74.32 |
| chr1 | 216260060 | 216260162 | 103 | *USH2A* | 284.4 |
| chr1 | 216262354 | 216262481 | 128 | *USH2A* | 319.61 |
| chr1 | 216270424 | 216270555 | 132 | *USH2A* | 241.34 |
| chr1 | 216348593 | 216348824 | 232 | *USH2A* | 254.55 |
| chr1 | 216363564 | 216363709 | 146 | *USH2A* | 271.88 |
| chr1 | 216369894 | 216370064 | 171 | *USH2A* | 234.3 |
| chr1 | 216371656 | 216371926 | 271 | *USH2A* | 155.41 |
| chr1 | 216372968 | 216373463 | 496 | *USH2A* | 151.58 |
| chr1 | 216380614 | 216380773 | 160 | *USH2A* | 346.54 |
| chr1 | 216390728 | 216390892 | 165 | *USH2A* | 321.44 |
| chr1 | 216405294 | 216405478 | 185 | *USH2A* | 240.51 |
| chr1 | 216419926 | 216420568 | 643 | *USH2A* | 156.09 |
| chr1 | 216424244 | 216424440 | 197 | *USH2A* | 309.1 |
| chr1 | 216462621 | 216462752 | 132 | *USH2A* | 368.23 |
| chr1 | 216465516 | 216465712 | 197 | *USH2A* | 320.15 |
| chr1 | 216495224 | 216495318 | 95 | *USH2A* | 273.47 |
| chr1 | 216496815 | 216497037 | 223 | *USH2A* | 127.75 |
| chr1 | 216497509 | 216497694 | 186 | *USH2A* | 74.01 |
| chr1 | 216498646 | 216498941 | 296 | *USH2A* | 223.5 |
| chr1 | 216500932 | 216500996 | 65 | *USH2A* | 319.03 |
| chr1 | 216538294 | 216538427 | 134 | *USH2A* | 357.08 |
| chr1 | 216591855 | 216592021 | 167 | *USH2A* | 275.35 |
| chr1 | 216595193 | 216595678 | 486 | *USH2A* | 144.45 |
| chr2 | 26680908 | 26681088 | 181 | *OTOF* | 11.79 |
| chr2 | 26682893 | 26683073 | 181 | *OTOF* | 283.92 |
| chr2 | 26683515 | 26683615 | 101 | *OTOF* | 174.84 |
| chr2 | 26683720 | 26683898 | 179 | *OTOF* | 136.5 |
| chr2 | 26684564 | 26684805 | 242 | *OTOF* | 338.17 |
| chr2 | 26684951 | 26685049 | 99 | *OTOF* | 194.31 |
| chr2 | 26686345 | 26686445 | 101 | *OTOF* | 209.42 |
| chr2 | 26686832 | 26686974 | 143 | *OTOF* | 309.16 |
| chr2 | 26687737 | 26687897 | 161 | *OTOF* | 117.73 |
| chr2 | 26688540 | 26688710 | 171 | *OTOF* | 123.82 |
| chr2 | 26688817 | 26688944 | 128 | *OTOF* | 164.09 |
| chr2 | 26689582 | 26689719 | 138 | *OTOF* | 60.74 |
| chr2 | 26689967 | 26690101 | 135 | *OTOF* | 37.64 |
| chr2 | 26690233 | 26690369 | 137 | *OTOF* | 32.16 |
| chr2 | 26691260 | 26691359 | 100 | *OTOF* | 75.44 |
| chr2 | 26693461 | 26693589 | 129 | *OTOF* | 322.11 |
| chr2 | 26693954 | 26694053 | 100 | *OTOF* | 258.03 |
| chr2 | 26695383 | 26695517 | 135 | *OTOF* | 137.69 |
| chr2 | 26696000 | 26696162 | 163 | *OTOF* | 78.14 |
| chr2 | 26696274 | 26696435 | 162 | *OTOF* | 87.01 |
| chr2 | 26696859 | 26696978 | 120 | *OTOF* | 72.61 |
| chr2 | 26697381 | 26697542 | 162 | *OTOF* | 142.31 |
| chr2 | 26698227 | 26698361 | 135 | *OTOF* | 122.47 |
| chr2 | 26698782 | 26698906 | 125 | *OTOF* | 109.66 |
| chr2 | 26698996 | 26699185 | 190 | *OTOF* | 129.62 |
| chr2 | 26699759 | 26699911 | 153 | *OTOF* | 107.96 |
| chr2 | 26700040 | 26700156 | 117 | *OTOF* | 112.29 |
| chr2 | 26700279 | 26700379 | 101 | *OTOF* | 157.8 |
| chr2 | 26700514 | 26700761 | 248 | *OTOF* | 198.58 |
| chr2 | 26702132 | 26702252 | 121 | *OTOF* | 376.04 |
| chr2 | 26702341 | 26702521 | 181 | *OTOF* | 204.23 |
| chr2 | 26703071 | 26703179 | 109 | *OTOF* | 149.6 |
| chr2 | 26703654 | 26703877 | 224 | *OTOF* | 123.74 |
| chr2 | 26705274 | 26705460 | 187 | *OTOF* | 405.09 |
| chr2 | 26706330 | 26706516 | 187 | *OTOF* | 256.06 |
| chr2 | 26707342 | 26707501 | 160 | *OTOF* | 176.57 |
| chr2 | 26712071 | 26712171 | 101 | *OTOF* | 325.47 |
| chr2 | 26712528 | 26712627 | 100 | *OTOF* | 272.47 |
| chr2 | 26717810 | 26717941 | 132 | *OTOF* | 429.66 |
| chr2 | 26724600 | 26724699 | 100 | *OTOF* | 146.02 |
| chr2 | 26725168 | 26725294 | 127 | *OTOF* | 131.4 |
| chr2 | 26726627 | 26726726 | 100 | *OTOF* | 323.58 |
| chr2 | 26739286 | 26739467 | 182 | *OTOF* | 331.3 |
| chr2 | 26741878 | 26741977 | 100 | *OTOF* | 337.66 |
| chr2 | 26750694 | 26750794 | 101 | *OTOF* | 306.94 |
| chr2 | 26760564 | 26760663 | 100 | *OTOF* | 307.85 |
| chr2 | 26781351 | 26781450 | 100 | *OTOF* | 256.04 |
| chr2 | 71163085 | 71163202 | 118 | *ATP6V1B1* | 246.64 |
| chr2 | 71170766 | 71170865 | 100 | *ATP6V1B1* | 204.94 |
| chr2 | 71185176 | 71185274 | 99 | *ATP6V1B1* | 237.34 |
| chr2 | 71185460 | 71185559 | 100 | *ATP6V1B1* | 208.17 |
| chr2 | 71186137 | 71186236 | 100 | *ATP6V1B1* | 155.06 |
| chr2 | 71187069 | 71187208 | 140 | *ATP6V1B1* | 399.98 |
| chr2 | 71188051 | 71188152 | 102 | *ATP6V1B1* | 241.86 |
| chr2 | 71188725 | 71188824 | 100 | *ATP6V1B1* | 247.68 |
| chr2 | 71189907 | 71190030 | 124 | *ATP6V1B1* | 318.15 |
| chr2 | 71190292 | 71190442 | 151 | *ATP6V1B1* | 314.23 |
| chr2 | 71190693 | 71190793 | 101 | *ATP6V1B1* | 242.33 |
| chr2 | 71191568 | 71191672 | 105 | *ATP6V1B1* | 264.78 |
| chr2 | 71191866 | 71191995 | 130 | *ATP6V1B1* | 207.61 |
| chr2 | 71192088 | 71192251 | 164 | *ATP6V1B1* | 255.98 |
| chr2 | 128015172 | 128015303 | 132 | *ERCC3* | 185.67 |
| chr2 | 128016872 | 128017024 | 153 | *ERCC3* | 129.52 |
| chr2 | 128018804 | 128018922 | 119 | *ERCC3* | 94.82 |
| chr2 | 128028912 | 128029029 | 118 | *ERCC3* | 98.88 |
| chr2 | 128030439 | 128030539 | 101 | *ERCC3* | 83.15 |
| chr2 | 128036749 | 128036951 | 203 | *ERCC3* | 139.47 |
| chr2 | 128038023 | 128038207 | 185 | *ERCC3* | 145.37 |
| chr2 | 128044279 | 128044593 | 315 | *ERCC3* | 406.78 |
| chr2 | 128046236 | 128046440 | 205 | *ERCC3* | 204.44 |
| chr2 | 128046913 | 128047077 | 165 | *ERCC3* | 229.67 |
| chr2 | 128047265 | 128047400 | 136 | *ERCC3* | 319.31 |
| chr2 | 128047775 | 128047874 | 100 | *ERCC3* | 307.29 |
| chr2 | 128050186 | 128050422 | 237 | *ERCC3* | 375.88 |
| chr2 | 128051089 | 128051294 | 206 | *ERCC3* | 216.61 |
| chr2 | 128051594 | 128051693 | 100 | *ERCC3* | 185.76 |
| chr2 | 179318136 | 179318347 | 212 | *DFNB59* | 115.03 |
| chr2 | 179319058 | 179319254 | 197 | *DFNB59* | 76.79 |
| chr2 | 179320736 | 179320878 | 143 | *DFNB59* | 105.38 |
| chr2 | 179323236 | 179323354 | 119 | *DFNB59* | 68.77 |
| chr2 | 179325074 | 179325173 | 100 | *DFNB59* | 64.15 |
| chr2 | 179325708 | 179326001 | 294 | *DFNB59* | 154.55 |
| chr2 | 219525711 | 219526030 | 320 | *BCS1L* | 249.36 |
| chr2 | 219526129 | 219526268 | 140 | *BCS1L* | 250.92 |
| chr2 | 219526482 | 219526676 | 195 | *BCS1L* | 198 |
| chr2 | 219526902 | 219527001 | 100 | *BCS1L* | 211.26 |
| chr2 | 219527233 | 219527402 | 170 | *BCS1L* | 184.44 |
| chr2 | 219527606 | 219527723 | 118 | *BCS1L* | 202.22 |
| chr2 | 219527857 | 219528109 | 253 | *BCS1L* | 191.17 |
| chr2 | 223065887 | 223065989 | 103 | *PAX3* | 154.49 |
| chr2 | 223066095 | 223066194 | 100 | *PAX3* | 191.24 |
| chr2 | 223066643 | 223066909 | 267 | *PAX3* | 222.47 |
| chr2 | 223084859 | 223085073 | 215 | *PAX3* | 319.9 |
| chr2 | 223085941 | 223086106 | 166 | *PAX3* | 296.36 |
| chr2 | 223096797 | 223097002 | 206 | *PAX3* | 374.67 |
| chr2 | 223158410 | 223158509 | 100 | *PAX3* | 278.81 |
| chr2 | 223158824 | 223159020 | 197 | *PAX3* | 340.32 |
| chr2 | 223160247 | 223160376 | 130 | *PAX3* | 324.39 |
| chr2 | 223161697 | 223161932 | 236 | *PAX3* | 363.37 |
| chr2 | 223163242 | 223163342 | 101 | *PAX3* | 271.39 |
| chr3 | 46747280 | 46747397 | 118 | *TMIE* | 408 |
| chr3 | 46750616 | 46750765 | 150 | *TMIE* | 310.92 |
| chr3 | 46751069 | 46751178 | 110 | *TMIE* | 333.75 |
| chr3 | 69788748 | 69788852 | 105 | *MITF* | 195.69 |
| chr3 | 69928284 | 69928534 | 251 | *MITF* | 166.46 |
| chr3 | 69986972 | 69987200 | 229 | *MITF* | 158.34 |
| chr3 | 69988248 | 69988332 | 85 | *MITF* | 52.58 |
| chr3 | 69990386 | 69990482 | 97 | *MITF* | 60.42 |
| chr3 | 69998201 | 69998319 | 119 | *MITF* | 81.11 |
| chr3 | 70000980 | 70001037 | 58 | *MITF* | 56.53 |
| chr3 | 70005605 | 70005681 | 77 | *MITF* | 37.62 |
| chr3 | 70008423 | 70008571 | 149 | *MITF* | 93.91 |
| chr3 | 70013997 | 70014399 | 403 | *MITF* | 224.66 |
| chr3 | 121707213 | 121707255 | 43 | *ILDR1* | 277.26 |
| chr3 | 121711996 | 121712817 | 822 | *ILDR1* | 344.33 |
| chr3 | 121713028 | 121713160 | 133 | *ILDR1* | 274.2 |
| chr3 | 121724090 | 121724240 | 151 | *ILDR1* | 272.79 |
| chr3 | 121725837 | 121726008 | 172 | *ILDR1* | 453.38 |
| chr3 | 121740866 | 121740924 | 59 | *ILDR1* | 296.61 |
| chr3 | 150644638 | 150644659 | 22 | *CLRN1* | 228.68 |
| chr3 | 150645722 | 150645988 | 267 | *CLRN1* | 255.87 |
| chr3 | 150658267 | 150658306 | 40 | *CLRN1* | 288.05 |
| chr3 | 150659368 | 150659548 | 181 | *CLRN1* | 301.82 |
| chr3 | 150661606 | 150661631 | 26 | *CLRN1* | 186.85 |
| chr3 | 150690242 | 150690495 | 254 | *CLRN1* | 378.13 |
| chr3 | 181430149 | 181431102 | 954 | *SOX2* | 84.25 |
| chr3 | 191047439 | 191047538 | 100 | *CCDC50* | 44.44 |
| chr3 | 191074859 | 191074958 | 100 | *CCDC50* | 24.41 |
| chr3 | 191075787 | 191075913 | 127 | *CCDC50* | 73.72 |
| chr3 | 191078853 | 191078953 | 101 | *CCDC50* | 56.51 |
| chr3 | 191087708 | 191087825 | 118 | *CCDC50* | 66.36 |
| chr3 | 191092851 | 191093378 | 528 | *CCDC50* | 239.89 |
| chr3 | 191097948 | 191098063 | 116 | *CCDC50* | 171.77 |
| chr3 | 191098449 | 191098548 | 100 | *CCDC50* | 198.77 |
| chr3 | 191098617 | 191098721 | 105 | *CCDC50* | 147.11 |
| chr3 | 191100525 | 191100624 | 100 | *CCDC50* | 32.87 |
| chr3 | 191107285 | 191107391 | 107 | *CCDC50* | 101.78 |
| chr3 | 191109490 | 191109589 | 100 | *CCDC50* | 50.45 |
| chr4 | 1800980 | 1801250 | 271 | *FGFR3* | 245.84 |
| chr4 | 1801473 | 1801539 | 67 | *FGFR3* | 286.66 |
| chr4 | 1803093 | 1803263 | 171 | *FGFR3* | 139.51 |
| chr4 | 1803346 | 1803470 | 125 | *FGFR3* | 126.77 |
| chr4 | 1803561 | 1803752 | 192 | *FGFR3* | 126.95 |
| chr4 | 1805418 | 1805563 | 146 | *FGFR3* | 102.53 |
| chr4 | 1806056 | 1806247 | 192 | *FGFR3* | 78.23 |
| chr4 | 1806550 | 1806696 | 147 | *FGFR3* | 80.29 |
| chr4 | 1807081 | 1807203 | 123 | *FGFR3* | 134.09 |
| chr4 | 1807285 | 1807396 | 112 | *FGFR3* | 212.51 |
| chr4 | 1807476 | 1807667 | 192 | *FGFR3* | 202.83 |
| chr4 | 1807777 | 1807900 | 124 | *FGFR3* | 236.74 |
| chr4 | 1807983 | 1808054 | 72 | *FGFR3* | 183.89 |
| chr4 | 1808272 | 1808410 | 139 | *FGFR3* | 144.33 |
| chr4 | 1808555 | 1808661 | 107 | *FGFR3* | 128.55 |
| chr4 | 1808842 | 1808989 | 148 | *FGFR3* | 215.34 |
| chr4 | 6279183 | 6279414 | 232 | *WFS1* | 134.68 |
| chr4 | 6288811 | 6288911 | 101 | *WFS1* | 59.28 |
| chr4 | 6290714 | 6290858 | 145 | *WFS1* | 68.3 |
| chr4 | 6292924 | 6293094 | 171 | *WFS1* | 114.81 |
| chr4 | 6293634 | 6293734 | 101 | *WFS1* | 36.13 |
| chr4 | 6296768 | 6296916 | 149 | *WFS1* | 108.89 |
| chr4 | 6302384 | 6304195 | 1812 | *WFS1* | 206.27 |
| chr4 | 42895260 | 42895765 | 506 | *GRXCR1* | 335.4 |
| chr4 | 42964816 | 42965170 | 355 | *GRXCR1* | 314.09 |
| chr4 | 43022249 | 43022567 | 319 | *GRXCR1* | 287.9 |
| chr4 | 43032358 | 43032701 | 344 | *GRXCR1* | 237.61 |
| chr4 | 88532036 | 88532136 | 101 | *DSPP* | 172.72 |
| chr4 | 88533249 | 88533348 | 100 | *DSPP* | 111.04 |
| chr4 | 88533474 | 88534460 | 987 | *DSPP* | 168.02 |
| chr4 | 88534937 | 88537720 | 2784 | *DSPP* | 593.22 |
| chr4 | 148406833 | 148407253 | 421 | *EDNRA* | 173.69 |
| chr4 | 148457028 | 148457181 | 154 | *EDNRA* | 108.18 |
| chr4 | 148460968 | 148461102 | 135 | *EDNRA* | 63.76 |
| chr4 | 148461528 | 148461637 | 110 | *EDNRA* | 52.06 |
| chr4 | 148463629 | 148463770 | 142 | *EDNRA* | 161.02 |
| chr5 | 68715213 | 68716358 | 1146 | *MARVELD2* | 208.51 |
| chr5 | 68720396 | 68720495 | 100 | *MARVELD2* | 267.17 |
| chr5 | 68728354 | 68728502 | 149 | *MARVELD2* | 189.87 |
| chr5 | 68728749 | 68728920 | 172 | *MARVELD2* | 153.35 |
| chr5 | 68736253 | 68736352 | 100 | *MARVELD2* | 240.23 |
| chr5 | 68737359 | 68737481 | 123 | *MARVELD2* | 116.75 |
| chr5 | 89854712 | 89854734 | 23 | *GPR98* | 222.13 |
| chr5 | 89910651 | 89910836 | 186 | *GPR98* | 221.23 |
| chr5 | 89913620 | 89913770 | 151 | *GPR98* | 69.28 |
| chr5 | 89914902 | 89914998 | 97 | *GPR98* | 16.65 |
| chr5 | 89918413 | 89918518 | 106 | *GPR98* | 304.79 |
| chr5 | 89920946 | 89921060 | 115 | *GPR98* | 327.64 |
| chr5 | 89923027 | 89923593 | 567 | *GPR98* | 216.49 |
| chr5 | 89924378 | 89924649 | 272 | *GPR98* | 134.38 |
| chr5 | 89925026 | 89925356 | 331 | *GPR98* | 110.02 |
| chr5 | 89930930 | 89931107 | 178 | *GPR98* | 235.2 |
| chr5 | 89933541 | 89933765 | 225 | *GPR98* | 255.22 |
| chr5 | 89938452 | 89938579 | 128 | *GPR98* | 93.8 |
| chr5 | 89938672 | 89938858 | 187 | *GPR98* | 101.51 |
| chr5 | 89939619 | 89939800 | 182 | *GPR98* | 95.84 |
| chr5 | 89940522 | 89940686 | 165 | *GPR98* | 71.73 |
| chr5 | 89941784 | 89941908 | 125 | *GPR98* | 90.93 |
| chr5 | 89943314 | 89943581 | 268 | *GPR98* | 341.64 |
| chr5 | 89947420 | 89947547 | 128 | *GPR98* | 111.73 |
| chr5 | 89948162 | 89948380 | 219 | *GPR98* | 98.9 |
| chr5 | 89949025 | 89949769 | 745 | *GPR98* | 218.61 |
| chr5 | 89953721 | 89954095 | 375 | *GPR98* | 240.94 |
| chr5 | 89968362 | 89968539 | 178 | *GPR98* | 47.33 |
| chr5 | 89969870 | 89970051 | 182 | *GPR98* | 52.21 |
| chr5 | 89971059 | 89971262 | 204 | *GPR98* | 200.42 |
| chr5 | 89971896 | 89972026 | 131 | *GPR98* | 93.04 |
| chr5 | 89975365 | 89975446 | 82 | *GPR98* | 303.38 |
| chr5 | 89977131 | 89977271 | 141 | *GPR98* | 346.9 |
| chr5 | 89979402 | 89980012 | 611 | *GPR98* | 230.3 |
| chr5 | 89981596 | 89981812 | 217 | *GPR98* | 218.29 |
| chr5 | 89985677 | 89985893 | 217 | *GPR98* | 99.65 |
| chr5 | 89986613 | 89986858 | 246 | *GPR98* | 154.83 |
| chr5 | 89988421 | 89988603 | 183 | *GPR98* | 294.5 |
| chr5 | 89989706 | 89990518 | 813 | *GPR98* | 188.14 |
| chr5 | 89992753 | 89992963 | 211 | *GPR98* | 270.09 |
| chr5 | 89999481 | 89999612 | 132 | *GPR98* | 149.12 |
| chr5 | 90000205 | 90000305 | 101 | *GPR98* | 161.59 |
| chr5 | 90001216 | 90001396 | 181 | *GPR98* | 197.68 |
| chr5 | 90002047 | 90002211 | 165 | *GPR98* | 191.42 |
| chr5 | 90004632 | 90004726 | 95 | *GPR98* | 224.35 |
| chr5 | 90006797 | 90006876 | 80 | *GPR98* | 148.66 |
| chr5 | 90007000 | 90007139 | 140 | *GPR98* | 173.96 |
| chr5 | 90008103 | 90008245 | 143 | *GPR98* | 215.02 |
| chr5 | 90012283 | 90012546 | 264 | *GPR98* | 246.08 |
| chr5 | 90015864 | 90016040 | 177 | *GPR98* | 52.23 |
| chr5 | 90016751 | 90016876 | 126 | *GPR98* | 50.17 |
| chr5 | 90020648 | 90020806 | 159 | *GPR98* | 144.24 |
| chr5 | 90020902 | 90021049 | 148 | *GPR98* | 92.39 |
| chr5 | 90021365 | 90021473 | 109 | *GPR98* | 110.71 |
| chr5 | 90024485 | 90024750 | 266 | *GPR98* | 94.83 |
| chr5 | 90025458 | 90025581 | 124 | *GPR98* | 82.87 |
| chr5 | 90040862 | 90041082 | 221 | *GPR98* | 194.67 |
| chr5 | 90041407 | 90041612 | 206 | *GPR98* | 155.07 |
| chr5 | 90046367 | 90046514 | 148 | *GPR98* | 318.09 |
| chr5 | 90049390 | 90049646 | 257 | *GPR98* | 218.35 |
| chr5 | 90050799 | 90051002 | 204 | *GPR98* | 292.13 |
| chr5 | 90052270 | 90052447 | 178 | *GPR98* | 194.78 |
| chr5 | 90052795 | 90052978 | 184 | *GPR98* | 243.71 |
| chr5 | 90055225 | 90055405 | 181 | *GPR98* | 234.15 |
| chr5 | 90059121 | 90059286 | 166 | *GPR98* | 331.97 |
| chr5 | 90070002 | 90070120 | 119 | *GPR98* | 341.81 |
| chr5 | 90072269 | 90072393 | 125 | *GPR98* | 335.06 |
| chr5 | 90073721 | 90073860 | 140 | *GPR98* | 136.74 |
| chr5 | 90074243 | 90074426 | 184 | *GPR98* | 116.46 |
| chr5 | 90074681 | 90074914 | 234 | *GPR98* | 130.93 |
| chr5 | 90077246 | 90077395 | 150 | *GPR98* | 240.5 |
| chr5 | 90078940 | 90079142 | 203 | *GPR98* | 170.09 |
| chr5 | 90079654 | 90079874 | 221 | *GPR98* | 130.05 |
| chr5 | 90083887 | 90084127 | 241 | *GPR98* | 315.66 |
| chr5 | 90085518 | 90085668 | 151 | *GPR98* | 71.72 |
| chr5 | 90086689 | 90087163 | 475 | *GPR98* | 132.14 |
| chr5 | 90098555 | 90098699 | 145 | *GPR98* | 298.46 |
| chr5 | 90101100 | 90101275 | 176 | *GPR98* | 272.23 |
| chr5 | 90103418 | 90103554 | 137 | *GPR98* | 262.39 |
| chr5 | 90106049 | 90107155 | 1107 | *GPR98* | 180.6 |
| chr5 | 90111435 | 90111553 | 119 | *GPR98* | 42.17 |
| chr5 | 90119241 | 90119413 | 173 | *GPR98* | 43.55 |
| chr5 | 90124760 | 90125003 | 244 | *GPR98* | 42.23 |
| chr5 | 90136394 | 90136802 | 409 | *GPR98* | 58.84 |
| chr5 | 90144453 | 90144638 | 186 | *GPR98* | 30.7 |
| chr5 | 90149100 | 90149350 | 251 | *GPR98* | 162.22 |
| chr5 | 90149878 | 90150018 | 141 | *GPR98* | 124.59 |
| chr5 | 90151557 | 90151718 | 162 | *GPR98* | 339.2 |
| chr5 | 90159573 | 90159674 | 102 | *GPR98* | 332 |
| chr5 | 90261231 | 90261348 | 118 | *GPR98* | 321.08 |
| chr5 | 90281160 | 90281339 | 180 | *GPR98* | 318.04 |
| chr5 | 90368263 | 90368421 | 159 | *GPR98* | 398.21 |
| chr5 | 90398035 | 90398157 | 123 | *GPR98* | 318.97 |
| chr5 | 90445846 | 90446038 | 193 | *GPR98* | 255.39 |
| chr5 | 90449037 | 90449215 | 179 | *GPR98* | 313.13 |
| chr5 | 90459598 | 90459717 | 120 | *GPR98* | 149.53 |
| chr5 | 92920649 | 92920810 | 162 | *NR2F1* | 20.56 |
| chr5 | 92920874 | 92921320 | 447 | *NR2F1* | 150.79 |
| chr5 | 92923623 | 92924150 | 528 | *NR2F1* | 310.52 |
| chr5 | 92929268 | 92929548 | 281 | *NR2F1* | 308.72 |
| chr5 | 140896418 | 140896575 | 158 | *DIAPH1* | 163.51 |
| chr5 | 140903703 | 140903803 | 101 | *DIAPH1* | 85.04 |
| chr5 | 140905605 | 140905740 | 136 | *DIAPH1* | 109.14 |
| chr5 | 140905864 | 140906028 | 165 | *DIAPH1* | 95.09 |
| chr5 | 140907140 | 140907264 | 125 | *DIAPH1* | 26.04 |
| chr5 | 140908020 | 140908149 | 130 | *DIAPH1* | 114.89 |
| chr5 | 140908269 | 140908508 | 240 | *DIAPH1* | 131.92 |
| chr5 | 140908739 | 140908840 | 102 | *DIAPH1* | 97.68 |
| chr5 | 140909167 | 140909267 | 101 | *DIAPH1* | 117.77 |
| chr5 | 140913902 | 140914000 | 99 | *DIAPH1* | 82.97 |
| chr5 | 140915576 | 140915675 | 100 | *DIAPH1* | 116.4 |
| chr5 | 140950949 | 140951049 | 101 | *DIAPH1* | 149.35 |
| chr5 | 140951493 | 140951607 | 115 | *DIAPH1* | 250.97 |
| chr5 | 140953059 | 140953775 | 717 | *DIAPH1* | 106.01 |
| chr5 | 140954534 | 140954713 | 180 | *DIAPH1* | 115.59 |
| chr5 | 140955779 | 140955879 | 101 | *DIAPH1* | 41.4 |
| chr5 | 140956323 | 140956438 | 116 | *DIAPH1* | 134.72 |
| chr5 | 140957042 | 140957158 | 117 | *DIAPH1* | 38.09 |
| chr5 | 140957792 | 140957910 | 119 | *DIAPH1* | 85.19 |
| chr5 | 140958082 | 140958192 | 111 | *DIAPH1* | 64.25 |
| chr5 | 140958655 | 140958763 | 109 | *DIAPH1* | 39.67 |
| chr5 | 140960311 | 140960450 | 140 | *DIAPH1* | 43.95 |
| chr5 | 140961861 | 140961960 | 100 | *DIAPH1* | 13.88 |
| chr5 | 140962766 | 140962866 | 101 | *DIAPH1* | 52.38 |
| chr5 | 140963052 | 140963182 | 131 | *DIAPH1* | 55.4 |
| chr5 | 140963691 | 140963792 | 102 | *DIAPH1* | 48.87 |
| chr5 | 140966609 | 140966764 | 156 | *DIAPH1* | 200.9 |
| chr5 | 140967754 | 140967854 | 101 | *DIAPH1* | 187.01 |
| chr5 | 145718676 | 145718795 | 120 | *POU4F3* | 145.66 |
| chr5 | 145719111 | 145720007 | 897 | *POU4F3* | 228.53 |
| chr5 | 147443585 | 147443685 | 101 | *SPINK5* | 237.49 |
| chr5 | 147444873 | 147444972 | 100 | *SPINK5* | 218.08 |
| chr5 | 147449886 | 147450013 | 128 | *SPINK5* | 218.98 |
| chr5 | 147451697 | 147451797 | 101 | *SPINK5* | 186.94 |
| chr5 | 147465968 | 147466095 | 128 | *SPINK5* | 211.45 |
| chr5 | 147468087 | 147468186 | 100 | *SPINK5* | 193.86 |
| chr5 | 147469057 | 147469184 | 128 | *SPINK5* | 167.6 |
| chr5 | 147470710 | 147470809 | 100 | *SPINK5* | 221.17 |
| chr5 | 147473917 | 147474044 | 128 | *SPINK5* | 338.84 |
| chr5 | 147475375 | 147475474 | 100 | *SPINK5* | 243.22 |
| chr5 | 147477430 | 147477557 | 128 | *SPINK5* | 274.3 |
| chr5 | 147478788 | 147478887 | 100 | *SPINK5* | 242.72 |
| chr5 | 147480017 | 147480144 | 128 | *SPINK5* | 353.71 |
| chr5 | 147480909 | 147481008 | 100 | *SPINK5* | 186.85 |
| chr5 | 147481344 | 147481471 | 128 | *SPINK5* | 289.09 |
| chr5 | 147484489 | 147484589 | 101 | *SPINK5* | 190.5 |
| chr5 | 147486600 | 147486727 | 128 | *SPINK5* | 212.68 |
| chr5 | 147488308 | 147488408 | 101 | *SPINK5* | 277.57 |
| chr5 | 147491331 | 147491458 | 128 | *SPINK5* | 327.06 |
| chr5 | 147492414 | 147492514 | 101 | *SPINK5* | 241.03 |
| chr5 | 147493925 | 147494052 | 128 | *SPINK5* | 276.61 |
| chr5 | 147495931 | 147496031 | 101 | *SPINK5* | 330.75 |
| chr5 | 147498000 | 147498127 | 128 | *SPINK5* | 167.96 |
| chr5 | 147498535 | 147498635 | 101 | *SPINK5* | 135.4 |
| chr5 | 147499572 | 147499699 | 128 | *SPINK5* | 242.59 |
| chr5 | 147499856 | 147499956 | 101 | *SPINK5* | 300.46 |
| chr5 | 147503396 | 147503523 | 128 | *SPINK5* | 240 |
| chr5 | 147504320 | 147504420 | 101 | *SPINK5* | 97.56 |
| chr5 | 147505082 | 147505181 | 100 | *SPINK5* | 232.87 |
| chr5 | 147505286 | 147505413 | 128 | *SPINK5* | 210.77 |
| chr5 | 147506544 | 147506644 | 101 | *SPINK5* | 169.23 |
| chr5 | 147510822 | 147510952 | 131 | *SPINK5* | 260.57 |
| chr5 | 147513355 | 147513455 | 101 | *SPINK5* | 345.22 |
| chr5 | 147516500 | 147516600 | 101 | *SPINK5* | 199.91 |
| chr5 | 169532961 | 169533535 | 575 | *FOXI1* | 284.61 |
| chr5 | 169535052 | 169535615 | 564 | *FOXI1* | 339.48 |
| chr6 | 2948531 | 2948933 | 403 | *SERPINB6* | 314.95 |
| chr6 | 2949147 | 2949303 | 157 | *SERPINB6* | 236.9 |
| chr6 | 2953277 | 2953420 | 144 | *SERPINB6* | 325.73 |
| chr6 | 2954825 | 2954943 | 119 | *SERPINB6* | 280.52 |
| chr6 | 2955757 | 2955904 | 148 | *SERPINB6* | 337.45 |
| chr6 | 2959401 | 2959566 | 166 | *SERPINB6* | 418.84 |
| chr6 | 33132629 | 33132741 | 113 | *COL11A2* | 44.87 |
| chr6 | 33133326 | 33133593 | 268 | *COL11A2* | 122.29 |
| chr6 | 33133685 | 33133784 | 100 | *COL11A2* | 152.23 |
| chr6 | 33133916 | 33134015 | 100 | *COL11A2* | 137.04 |
| chr6 | 33134267 | 33134366 | 100 | *COL11A2* | 149.17 |
| chr6 | 33134824 | 33134923 | 100 | *COL11A2* | 170.96 |
| chr6 | 33136460 | 33136559 | 100 | *COL11A2* | 40.37 |
| chr6 | 33137585 | 33137684 | 100 | *COL11A2* | 27.34 |
| chr6 | 33138868 | 33138967 | 100 | *COL11A2* | 37.52 |
| chr6 | 33139025 | 33139124 | 100 | *COL11A2* | 78.33 |
| chr6 | 33139244 | 33139351 | 108 | *COL11A2* | 63.88 |
| chr6 | 33141256 | 33141355 | 100 | *COL11A2* | 114.22 |
| chr6 | 33141627 | 33141726 | 100 | *COL11A2* | 227.8 |
| chr6 | 33144471 | 33144570 | 100 | *COL11A2* | 128.84 |
| chr6 | 33146827 | 33146926 | 100 | *COL11A2* | 74.37 |
| chr6 | 33147489 | 33147589 | 101 | *COL11A2* | 120.49 |
| chr6 | 33148712 | 33148811 | 100 | *COL11A2* | 115.29 |
| chr6 | 33148868 | 33148967 | 100 | *COL11A2* | 129.09 |
| chr6 | 33151923 | 33152101 | 179 | *COL11A2* | 279.23 |
| chr6 | 33152751 | 33152850 | 100 | *COL11A2* | 185.56 |
| chr6 | 33154330 | 33154595 | 266 | *COL11A2* | 314.6 |
| chr6 | 33156755 | 33156965 | 211 | *COL11A2* | 309.39 |
| chr6 | 35773448 | 35773859 | 412 | *LHFPL5* | 379.68 |
| chr6 | 35782323 | 35782559 | 237 | *LHFPL5* | 439.23 |
| chr6 | 35787170 | 35787269 | 100 | *LHFPL5* | 178.74 |
| chr6 | 76527265 | 76527381 | 117 | *MYO6* | 111 |
| chr6 | 76532484 | 76532583 | 100 | *MYO6* | 34.26 |
| chr6 | 76538244 | 76538343 | 100 | *MYO6* | 27.82 |
| chr6 | 76540133 | 76540262 | 130 | *MYO6* | 44.35 |
| chr6 | 76542559 | 76542664 | 106 | *MYO6* | 44.07 |
| chr6 | 76545596 | 76545695 | 100 | *MYO6* | 11.76 |
| chr6 | 76550301 | 76550400 | 100 | *MYO6* | 32.59 |
| chr6 | 76550931 | 76551095 | 165 | *MYO6* | 55.67 |
| chr6 | 76554604 | 76554704 | 101 | *MYO6* | 35.09 |
| chr6 | 76558068 | 76558248 | 181 | *MYO6* | 87.91 |
| chr6 | 76564856 | 76565000 | 145 | *MYO6* | 77.81 |
| chr6 | 76566814 | 76566971 | 158 | *MYO6* | 100.61 |
| chr6 | 76568615 | 76568714 | 100 | *MYO6* | 39.93 |
| chr6 | 76570727 | 76570826 | 100 | *MYO6* | 210.51 |
| chr6 | 76572313 | 76572440 | 128 | *MYO6* | 250.61 |
| chr6 | 76576241 | 76576340 | 100 | *MYO6* | 182.35 |
| chr6 | 76576649 | 76576822 | 174 | *MYO6* | 166.84 |
| chr6 | 76580334 | 76580433 | 100 | *MYO6* | 205.33 |
| chr6 | 76582921 | 76583020 | 100 | *MYO6* | 225.55 |
| chr6 | 76589537 | 76589667 | 131 | *MYO6* | 235.71 |
| chr6 | 76589749 | 76589848 | 100 | *MYO6* | 176.74 |
| chr6 | 76591406 | 76591535 | 130 | *MYO6* | 344.89 |
| chr6 | 76595716 | 76595816 | 101 | *MYO6* | 139.46 |
| chr6 | 76596561 | 76596711 | 151 | *MYO6* | 232.28 |
| chr6 | 76599774 | 76599982 | 209 | *MYO6* | 342.02 |
| chr6 | 76600935 | 76601034 | 100 | *MYO6* | 205.66 |
| chr6 | 76602247 | 76602407 | 161 | *MYO6* | 321.66 |
| chr6 | 76604913 | 76605012 | 100 | *MYO6* | 182.59 |
| chr6 | 76608060 | 76608159 | 100 | *MYO6* | 125.35 |
| chr6 | 76617322 | 76617425 | 104 | *MYO6* | 128.15 |
| chr6 | 76618213 | 76618344 | 132 | *MYO6* | 131.18 |
| chr6 | 76621353 | 76621452 | 100 | *MYO6* | 206.62 |
| chr6 | 76623780 | 76623998 | 219 | *MYO6* | 165.97 |
| chr6 | 76624530 | 76624729 | 200 | *MYO6* | 211.91 |
| chr6 | 121767994 | 121769142 | 1149 | *GJA1* | 134.93 |
| chr6 | 133595886 | 133595985 | 100 | *EYA4* | 74.83 |
| chr6 | 133703505 | 133703604 | 100 | *EYA4* | 42.24 |
| chr6 | 133767768 | 133767892 | 125 | *EYA4* | 118.28 |
| chr6 | 133769234 | 133769333 | 100 | *EYA4* | 371.84 |
| chr6 | 133777690 | 133777790 | 101 | *EYA4* | 113.46 |
| chr6 | 133782236 | 133782335 | 100 | *EYA4* | 47.51 |
| chr6 | 133783473 | 133783615 | 143 | *EYA4* | 162.69 |
| chr6 | 133783759 | 133783902 | 144 | *EYA4* | 140.78 |
| chr6 | 133785907 | 133786006 | 100 | *EYA4* | 42.42 |
| chr6 | 133789704 | 133789869 | 166 | *EYA4* | 142.68 |
| chr6 | 133802601 | 133802737 | 137 | *EYA4* | 127.43 |
| chr6 | 133804162 | 133804261 | 100 | *EYA4* | 76.64 |
| chr6 | 133827239 | 133827338 | 100 | *EYA4* | 234.54 |
| chr6 | 133833839 | 133833938 | 100 | *EYA4* | 374.84 |
| chr6 | 133834016 | 133834176 | 161 | *EYA4* | 327.3 |
| chr6 | 133836459 | 133836573 | 115 | *EYA4* | 371.61 |
| chr6 | 133844194 | 133844315 | 122 | *EYA4* | 325.39 |
| chr6 | 133846153 | 133846253 | 101 | *EYA4* | 159 |
| chr6 | 133846292 | 133846392 | 101 | *EYA4* | 344.31 |
| chr6 | 133849853 | 133849953 | 101 | *EYA4* | 269.93 |
| chr6 | 134210536 | 134210985 | 450 | *TCF21* | 252.37 |
| chr6 | 134212846 | 134212945 | 100 | *TCF21* | 204.98 |
| chr7 | 5567379 | 5567522 | 144 | *ACTB* | 116.16 |
| chr7 | 5567635 | 5567816 | 182 | *ACTB* | 132.52 |
| chr7 | 5567912 | 5568350 | 439 | *ACTB* | 152.62 |
| chr7 | 5568792 | 5569031 | 240 | *ACTB* | 232.3 |
| chr7 | 5569166 | 5569288 | 123 | *ACTB* | 202.87 |
| chr7 | 24738645 | 24738878 | 234 | *DFNA5* | 185.14 |
| chr7 | 24742366 | 24742465 | 100 | *DFNA5* | 54.1 |
| chr7 | 24745803 | 24745995 | 193 | *DFNA5* | 93.3 |
| chr7 | 24747746 | 24747873 | 128 | *DFNA5* | 103.37 |
| chr7 | 24749843 | 24750007 | 165 | *DFNA5* | 116.49 |
| chr7 | 24756873 | 24756993 | 121 | *DFNA5* | 92.95 |
| chr7 | 24758666 | 24758837 | 172 | *DFNA5* | 103.18 |
| chr7 | 24784181 | 24784373 | 193 | *DFNA5* | 164.35 |
| chr7 | 24789183 | 24789393 | 211 | *DFNA5* | 124.37 |
| chr7 | 81331896 | 81332073 | 178 | *HGF* | 350.79 |
| chr7 | 81334705 | 81334851 | 147 | *HGF* | 292.8 |
| chr7 | 81334962 | 81335069 | 108 | *HGF* | 231.7 |
| chr7 | 81335602 | 81335743 | 142 | *HGF* | 310.2 |
| chr7 | 81336605 | 81336680 | 76 | *HGF* | 291.84 |
| chr7 | 81339462 | 81339559 | 98 | *HGF* | 346.48 |
| chr7 | 81340796 | 81340835 | 40 | *HGF* | 197.9 |
| chr7 | 81346547 | 81346681 | 135 | *HGF* | 403.96 |
| chr7 | 81350060 | 81350163 | 104 | *HGF* | 289.06 |
| chr7 | 81355205 | 81355333 | 129 | *HGF* | 391.22 |
| chr7 | 81358920 | 81359095 | 176 | *HGF* | 325.29 |
| chr7 | 81372257 | 81372265 | 9 | *HGF* | 295.11 |
| chr7 | 81372668 | 81372787 | 120 | *HGF* | 236.69 |
| chr7 | 81374315 | 81374436 | 122 | *HGF* | 314.28 |
| chr7 | 81381435 | 81381563 | 129 | *HGF* | 302.57 |
| chr7 | 81386504 | 81386619 | 116 | *HGF* | 343.66 |
| chr7 | 81388007 | 81388120 | 114 | *HGF* | 236.3 |
| chr7 | 81392022 | 81392188 | 167 | *HGF* | 245.81 |
| chr7 | 81399199 | 81399287 | 89 | *HGF* | 423.94 |
| chr7 | 102993306 | 102993405 | 100 | *SLC26A5* | 137.97 |
| chr7 | 103014846 | 103015039 | 194 | *SLC26A5* | 280.05 |
| chr7 | 103017233 | 103017332 | 100 | *SLC26A5* | 210.62 |
| chr7 | 103018046 | 103018246 | 201 | *SLC26A5* | 328.91 |
| chr7 | 103018893 | 103019000 | 108 | *SLC26A5* | 210.81 |
| chr7 | 103019686 | 103019786 | 101 | *SLC26A5* | 111.03 |
| chr7 | 103020912 | 103021011 | 100 | *SLC26A5* | 179.9 |
| chr7 | 103029455 | 103029561 | 107 | *SLC26A5* | 242.53 |
| chr7 | 103029774 | 103029873 | 100 | *SLC26A5* | 297.97 |
| chr7 | 103030865 | 103030964 | 100 | *SLC26A5* | 120.4 |
| chr7 | 103032069 | 103032182 | 114 | *SLC26A5* | 129.41 |
| chr7 | 103033366 | 103033513 | 148 | *SLC26A5* | 145.22 |
| chr7 | 103038370 | 103038470 | 101 | *SLC26A5* | 289.7 |
| chr7 | 103048298 | 103048450 | 153 | *SLC26A5* | 331.39 |
| chr7 | 103050832 | 103050996 | 165 | *SLC26A5* | 295.39 |
| chr7 | 103051867 | 103052033 | 167 | *SLC26A5* | 168.12 |
| chr7 | 103053449 | 103053559 | 111 | *SLC26A5* | 132.57 |
| chr7 | 103061185 | 103061324 | 140 | *SLC26A5* | 242.28 |
| chr7 | 103061810 | 103061961 | 152 | *SLC26A5* | 255.14 |
| chr7 | 107302087 | 107302250 | 164 | *SLC26A4* | 123.98 |
| chr7 | 107303741 | 107303880 | 140 | *SLC26A4* | 168.29 |
| chr7 | 107312583 | 107312693 | 111 | *SLC26A4* | 77.05 |
| chr7 | 107314609 | 107314793 | 185 | *SLC26A4* | 154.14 |
| chr7 | 107315390 | 107315554 | 165 | *SLC26A4* | 136.01 |
| chr7 | 107323647 | 107323799 | 153 | *SLC26A4* | 157.85 |
| chr7 | 107323891 | 107323991 | 101 | *SLC26A4* | 125.27 |
| chr7 | 107329498 | 107329645 | 148 | *SLC26A4* | 161.07 |
| chr7 | 107330569 | 107330682 | 114 | *SLC26A4* | 117.32 |
| chr7 | 107334837 | 107334936 | 100 | *SLC26A4* | 127.72 |
| chr7 | 107335064 | 107335163 | 100 | *SLC26A4* | 124.21 |
| chr7 | 107336378 | 107336484 | 107 | *SLC26A4* | 120.36 |
| chr7 | 107338472 | 107338571 | 100 | *SLC26A4* | 54.39 |
| chr7 | 107340524 | 107340624 | 101 | *SLC26A4* | 90.12 |
| chr7 | 107341544 | 107341643 | 100 | *SLC26A4* | 94.44 |
| chr7 | 107342272 | 107342502 | 231 | *SLC26A4* | 183.95 |
| chr7 | 107344754 | 107344853 | 100 | *SLC26A4* | 34.18 |
| chr7 | 107350499 | 107350644 | 146 | *SLC26A4* | 142.62 |
| chr7 | 107352976 | 107353075 | 100 | *SLC26A4* | 66.38 |
| chr7 | 107355830 | 107355929 | 100 | *SLC26A4* | 141.6 |
| chr7 | 129410145 | 129410432 | 288 | *MIR182* | 413.26 |
| chr7 | 129414479 | 129414834 | 356 | *MIR183* | 329.64 |
| chr8 | 20054917 | 20055053 | 137 | *ATP6V1B2* | 305.91 |
| chr8 | 20061994 | 20062050 | 57 | *ATP6V1B2* | 253.51 |
| chr8 | 20066943 | 20067042 | 100 | *ATP6V1B2* | 373.46 |
| chr8 | 20067856 | 20067950 | 95 | *ATP6V1B2* | 240.91 |
| chr8 | 20068079 | 20068157 | 79 | *ATP6V1B2* | 243.42 |
| chr8 | 20068687 | 20068827 | 141 | *ATP6V1B2* | 277.12 |
| chr8 | 20069162 | 20069264 | 103 | *ATP6V1B2* | 171.25 |
| chr8 | 20069612 | 20069710 | 99 | *ATP6V1B2* | 246.38 |
| chr8 | 20070292 | 20070416 | 125 | *ATP6V1B2* | 184.51 |
| chr8 | 20072328 | 20072479 | 152 | *ATP6V1B2* | 345.76 |
| chr8 | 20073923 | 20074006 | 84 | *ATP6V1B2* | 64.38 |
| chr8 | 20074730 | 20074835 | 106 | *ATP6V1B2* | 369.83 |
| chr8 | 20075663 | 20075793 | 131 | *ATP6V1B2* | 377.64 |
| chr8 | 20077773 | 20077913 | 141 | *ATP6V1B2* | 347.72 |
| chr8 | 49831365 | 49831547 | 183 | *SNAI2* | 375.84 |
| chr8 | 49832454 | 49833000 | 547 | *SNAI2* | 326.61 |
| chr8 | 49833745 | 49833824 | 80 | *SNAI2* | 369.41 |
| chr8 | 102504958 | 102505057 | 100 | *GRHL2* | 245.76 |
| chr8 | 102555469 | 102555664 | 196 | *GRHL2* | 113.3 |
| chr8 | 102564927 | 102565026 | 100 | *GRHL2* | 39.89 |
| chr8 | 102570647 | 102571040 | 394 | *GRHL2* | 114.08 |
| chr8 | 102582545 | 102582644 | 100 | *GRHL2* | 32.96 |
| chr8 | 102585896 | 102586052 | 157 | *GRHL2* | 89.92 |
| chr8 | 102589636 | 102589747 | 112 | *GRHL2* | 46.73 |
| chr8 | 102611282 | 102611382 | 101 | *GRHL2* | 37.52 |
| chr8 | 102631767 | 102631925 | 159 | *GRHL2* | 54.28 |
| chr8 | 102643859 | 102643958 | 100 | *GRHL2* | 48.46 |
| chr8 | 102644454 | 102644593 | 140 | *GRHL2* | 65.76 |
| chr8 | 102649091 | 102649190 | 100 | *GRHL2* | 282.85 |
| chr8 | 102656356 | 102656456 | 101 | *GRHL2* | 65.14 |
| chr8 | 102661635 | 102661734 | 100 | *GRHL2* | 54.38 |
| chr8 | 102676665 | 102676764 | 100 | *GRHL2* | 36.45 |
| chr8 | 102678817 | 102678931 | 115 | *GRHL2* | 71.49 |
| chr9 | 21802715 | 21802814 | 100 | *MTAP* | 82.89 |
| chr9 | 21815425 | 21815525 | 101 | *MTAP* | 52.77 |
| chr9 | 21816693 | 21816792 | 100 | *MTAP* | 28.62 |
| chr9 | 21818034 | 21818201 | 168 | *MTAP* | 106.11 |
| chr9 | 21837907 | 21838009 | 103 | *MTAP* | 70.64 |
| chr9 | 21854630 | 21854869 | 240 | *MTAP* | 96.88 |
| chr9 | 21859302 | 21859424 | 123 | *MTAP* | 38.29 |
| chr9 | 21861945 | 21862044 | 100 | *MTAP* | 79.06 |
| chr9 | 71789288 | 71789348 | 61 | *TJP2* | 343.66 |
| chr9 | 71820077 | 71820230 | 154 | *TJP2* | 350.16 |
| chr9 | 71827463 | 71827517 | 55 | *TJP2* | 330.44 |
| chr9 | 71831254 | 71831379 | 126 | *TJP2* | 291.89 |
| chr9 | 71833172 | 71833275 | 104 | *TJP2* | 349.2 |
| chr9 | 71840219 | 71840323 | 105 | *TJP2* | 323.1 |
| chr9 | 71840937 | 71841091 | 155 | *TJP2* | 335.46 |
| chr9 | 71842680 | 71842789 | 110 | *TJP2* | 190.53 |
| chr9 | 71842896 | 71843030 | 135 | *TJP2* | 337.1 |
| chr9 | 71844099 | 71844166 | 68 | *TJP2* | 376.29 |
| chr9 | 71844997 | 71845148 | 152 | *TJP2* | 395.93 |
| chr9 | 71849354 | 71849463 | 110 | *TJP2* | 225.56 |
| chr9 | 71850943 | 71851154 | 212 | *TJP2* | 362.36 |
| chr9 | 71851864 | 71852052 | 189 | *TJP2* | 350.54 |
| chr9 | 71852793 | 71852889 | 97 | *TJP2* | 322.99 |
| chr9 | 71853625 | 71853705 | 81 | *TJP2* | 365.44 |
| chr9 | 71854852 | 71855063 | 212 | *TJP2* | 340.89 |
| chr9 | 71861605 | 71861706 | 102 | *TJP2* | 358.7 |
| chr9 | 71862927 | 71863140 | 214 | *TJP2* | 383.89 |
| chr9 | 71864290 | 71864401 | 112 | *TJP2* | 353.69 |
| chr9 | 71865950 | 71866280 | 331 | *TJP2* | 378.62 |
| chr9 | 71867730 | 71867816 | 87 | *TJP2* | 354.1 |
| chr9 | 71869124 | 71869290 | 167 | *TJP2* | 345.8 |
| chr9 | 75263523 | 75263622 | 100 | *TMC1* | 351.29 |
| chr9 | 75303599 | 75303698 | 100 | *TMC1* | 183.67 |
| chr9 | 75309459 | 75309630 | 172 | *TMC1* | 334.88 |
| chr9 | 75315434 | 75315559 | 126 | *TMC1* | 200.63 |
| chr9 | 75355030 | 75355130 | 101 | *TMC1* | 181.34 |
| chr9 | 75357351 | 75357450 | 100 | *TMC1* | 348.78 |
| chr9 | 75366766 | 75366872 | 107 | *TMC1* | 193.42 |
| chr9 | 75369702 | 75369800 | 99 | *TMC1* | 276.11 |
| chr9 | 75387329 | 75387471 | 143 | *TMC1* | 298.64 |
| chr9 | 75403255 | 75403399 | 145 | *TMC1* | 40.5 |
| chr9 | 75404039 | 75404233 | 195 | *TMC1* | 154.42 |
| chr9 | 75406802 | 75406981 | 180 | *TMC1* | 98.73 |
| chr9 | 75407107 | 75407268 | 162 | *TMC1* | 93.94 |
| chr9 | 75420298 | 75420426 | 129 | *TMC1* | 59.43 |
| chr9 | 75431043 | 75431142 | 100 | *TMC1* | 48.74 |
| chr9 | 75435758 | 75435997 | 240 | *TMC1* | 136.61 |
| chr9 | 75441785 | 75441910 | 126 | *TMC1* | 103.03 |
| chr9 | 75445357 | 75445456 | 100 | *TMC1* | 50.95 |
| chr9 | 75445523 | 75445622 | 100 | *TMC1* | 37.5 |
| chr9 | 75450829 | 75450928 | 100 | *TMC1* | 76.01 |
| chr9 | 117165034 | 117165216 | 183 | *DFNB31* | 132.62 |
| chr9 | 117165497 | 117165619 | 123 | *DFNB31* | 103.57 |
| chr9 | 117166176 | 117166357 | 182 | *DFNB31* | 171.37 |
| chr9 | 117168635 | 117169172 | 538 | *DFNB31* | 384.93 |
| chr9 | 117170213 | 117170312 | 100 | *DFNB31* | 370.33 |
| chr9 | 117185594 | 117185803 | 210 | *DFNB31* | 395.83 |
| chr9 | 117186614 | 117186826 | 213 | *DFNB31* | 276.53 |
| chr9 | 117187237 | 117187336 | 100 | *DFNB31* | 174.82 |
| chr9 | 117188491 | 117188693 | 203 | *DFNB31* | 271.76 |
| chr9 | 117228547 | 117228672 | 126 | *DFNB31* | 282.05 |
| chr9 | 117240833 | 117241051 | 219 | *DFNB31* | 261.01 |
| chr9 | 117266464 | 117267081 | 618 | *DFNB31* | 368.91 |
| chr9 | 139089171 | 139089589 | 419 | *LHX3* | 120.29 |
| chr9 | 139090498 | 139090666 | 169 | *LHX3* | 122.14 |
| chr9 | 139090754 | 139090905 | 152 | *LHX3* | 84.23 |
| chr9 | 139091524 | 139091726 | 203 | *LHX3* | 223.13 |
| chr9 | 139092428 | 139092599 | 172 | *LHX3* | 347.58 |
| chr9 | 139094789 | 139094888 | 100 | *LHX3* | 51.24 |
| chr9 | 139096770 | 139096869 | 100 | *LHX3* | 109.77 |
| chr9 | 140093099 | 140094500 | 1402 | *TPRN* | 219.6 |
| chr10 | 8097619 | 8097859 | 241 | *GATA3* | 303.2 |
| chr10 | 8100268 | 8100804 | 537 | *GATA3* | 185.26 |
| chr10 | 8105956 | 8106101 | 146 | *GATA3* | 308.24 |
| chr10 | 8111436 | 8111561 | 126 | *GATA3* | 379.53 |
| chr10 | 8115702 | 8115986 | 285 | *GATA3* | 375.34 |
| chr10 | 26241040 | 26241207 | 168 | *MYO3A* | 262.83 |
| chr10 | 26243803 | 26243937 | 135 | *MYO3A* | 199.94 |
| chr10 | 26285419 | 26285523 | 105 | *MYO3A* | 176.33 |
| chr10 | 26286088 | 26286187 | 100 | *MYO3A* | 188.18 |
| chr10 | 26305738 | 26305837 | 100 | *MYO3A* | 275.83 |
| chr10 | 26310432 | 26310577 | 146 | *MYO3A* | 277.1 |
| chr10 | 26312934 | 26313033 | 100 | *MYO3A* | 236.01 |
| chr10 | 26315306 | 26315461 | 156 | *MYO3A* | 285.93 |
| chr10 | 26355904 | 26356003 | 100 | *MYO3A* | 260.24 |
| chr10 | 26357697 | 26357813 | 117 | *MYO3A* | 97.61 |
| chr10 | 26359040 | 26359144 | 105 | *MYO3A* | 101.11 |
| chr10 | 26359239 | 26359338 | 100 | *MYO3A* | 87.48 |
| chr10 | 26377132 | 26377334 | 203 | *MYO3A* | 173.67 |
| chr10 | 26385310 | 26385408 | 99 | *MYO3A* | 32.98 |
| chr10 | 26385497 | 26385611 | 115 | *MYO3A* | 29.52 |
| chr10 | 26409605 | 26409731 | 127 | *MYO3A* | 43.15 |
| chr10 | 26414327 | 26414537 | 211 | *MYO3A* | 63.35 |
| chr10 | 26417320 | 26417467 | 148 | *MYO3A* | 35.66 |
| chr10 | 26432377 | 26432530 | 154 | *MYO3A* | 37.64 |
| chr10 | 26434369 | 26434469 | 101 | *MYO3A* | 37.58 |
| chr10 | 26436359 | 26436488 | 130 | *MYO3A* | 57.5 |
| chr10 | 26442769 | 26442868 | 100 | *MYO3A* | 18.49 |
| chr10 | 26443664 | 26443763 | 100 | *MYO3A* | 384.83 |
| chr10 | 26446239 | 26446444 | 206 | *MYO3A* | 303.14 |
| chr10 | 26454996 | 26455107 | 112 | *MYO3A* | 311.14 |
| chr10 | 26457641 | 26457803 | 163 | *MYO3A* | 295.5 |
| chr10 | 26459345 | 26459468 | 124 | *MYO3A* | 272.41 |
| chr10 | 26462592 | 26463486 | 895 | *MYO3A* | 195.89 |
| chr10 | 26465630 | 26465774 | 145 | *MYO3A* | 165.66 |
| chr10 | 26482134 | 26482240 | 107 | *MYO3A* | 336.28 |
| chr10 | 26490165 | 26490264 | 100 | *MYO3A* | 36.29 |
| chr10 | 26491893 | 26492036 | 144 | *MYO3A* | 208.58 |
| chr10 | 26500772 | 26500892 | 121 | *MYO3A* | 322.99 |
| chr10 | 55566340 | 55566890 | 551 | *PCDH15* | 234.64 |
| chr10 | 55568454 | 55569306 | 853 | *PCDH15* | 202.35 |
| chr10 | 55570316 | 55570426 | 111 | *PCDH15* | 327.84 |
| chr10 | 55571295 | 55571395 | 101 | *PCDH15* | 112.02 |
| chr10 | 55581618 | 55583118 | 1501 | *PCDH15* | 46.35 |
| chr10 | 55583142 | 55583241 | 100 | *PCDH15* | 25.35 |
| chr10 | 55584848 | 55584948 | 101 | *PCDH15* | 229.67 |
| chr10 | 55587150 | 55587308 | 159 | *PCDH15* | 134.4 |
| chr10 | 55588280 | 55588379 | 100 | *PCDH15* | 88.23 |
| chr10 | 55591075 | 55591293 | 219 | *PCDH15* | 246.69 |
| chr10 | 55600080 | 55600256 | 177 | *PCDH15* | 271.99 |
| chr10 | 55616929 | 55617029 | 101 | *PCDH15* | 217.5 |
| chr10 | 55626402 | 55626617 | 216 | *PCDH15* | 284.59 |
| chr10 | 55663003 | 55663130 | 128 | *PCDH15* | 298.62 |
| chr10 | 55698575 | 55698715 | 141 | *PCDH15* | 251.23 |
| chr10 | 55700626 | 55700735 | 110 | *PCDH15* | 209.34 |
| chr10 | 55719492 | 55719604 | 113 | *PCDH15* | 243.18 |
| chr10 | 55721512 | 55721652 | 141 | *PCDH15* | 125.94 |
| chr10 | 55755409 | 55755525 | 117 | *PCDH15* | 359.59 |
| chr10 | 55779952 | 55780176 | 225 | *PCDH15* | 333.55 |
| chr10 | 55782652 | 55782957 | 306 | *PCDH15* | 269.74 |
| chr10 | 55826517 | 55826645 | 129 | *PCDH15* | 185.12 |
| chr10 | 55839088 | 55839187 | 100 | *PCDH15* | 304.94 |
| chr10 | 55849734 | 55849833 | 100 | *PCDH15* | 211.02 |
| chr10 | 55892635 | 55892767 | 133 | *PCDH15* | 271.23 |
| chr10 | 55912860 | 55913053 | 194 | *PCDH15* | 305.35 |
| chr10 | 55943204 | 55943353 | 150 | *PCDH15* | 265.61 |
| chr10 | 55944894 | 55945028 | 135 | *PCDH15* | 224.35 |
| chr10 | 55949085 | 55949184 | 100 | *PCDH15* | 200.81 |
| chr10 | 55955443 | 55955649 | 207 | *PCDH15* | 284.46 |
| chr10 | 55973696 | 55973808 | 113 | *PCDH15* | 286.08 |
| chr10 | 55996583 | 55996691 | 109 | *PCDH15* | 175.74 |
| chr10 | 56077031 | 56077201 | 171 | *PCDH15* | 330.02 |
| chr10 | 56089356 | 56089466 | 111 | *PCDH15* | 207.32 |
| chr10 | 56106125 | 56106244 | 120 | *PCDH15* | 286.28 |
| chr10 | 56128880 | 56129035 | 156 | *PCDH15* | 165.07 |
| chr10 | 56138542 | 56138702 | 161 | *PCDH15* | 194.66 |
| chr10 | 56287555 | 56287654 | 100 | *PCDH15* | 140.25 |
| chr10 | 56288106 | 56288205 | 100 | *PCDH15* | 287.52 |
| chr10 | 56367626 | 56367726 | 101 | *PCDH15* | 160.68 |
| chr10 | 56423927 | 56424027 | 101 | *PCDH15* | 199.75 |
| chr10 | 73199572 | 73199672 | 101 | *CDH23* | 340.71 |
| chr10 | 73206064 | 73206163 | 100 | *CDH23* | 445.55 |
| chr10 | 73269839 | 73269981 | 143 | *CDH23* | 301.43 |
| chr10 | 73270685 | 73270784 | 100 | *CDH23* | 206.04 |
| chr10 | 73270873 | 73270973 | 101 | *CDH23* | 247.31 |
| chr10 | 73326499 | 73326693 | 195 | *CDH23* | 300.08 |
| chr10 | 73330547 | 73330675 | 129 | *CDH23* | 456.21 |
| chr10 | 73337660 | 73337760 | 101 | *CDH23* | 391.64 |
| chr10 | 73375261 | 73375373 | 113 | *CDH23* | 303.12 |
| chr10 | 73376962 | 73377150 | 189 | *CDH23* | 233.82 |
| chr10 | 73403571 | 73403670 | 100 | *CDH23* | 438.78 |
| chr10 | 73405583 | 73405737 | 155 | *CDH23* | 183.21 |
| chr10 | 73406216 | 73406518 | 303 | *CDH23* | 157.11 |
| chr10 | 73434852 | 73434951 | 100 | *CDH23* | 366.26 |
| chr10 | 73437214 | 73437450 | 237 | *CDH23* | 350.88 |
| chr10 | 73439145 | 73439249 | 105 | *CDH23* | 254.54 |
| chr10 | 73442203 | 73442329 | 127 | *CDH23* | 379.39 |
| chr10 | 73447391 | 73447490 | 100 | *CDH23* | 394.14 |
| chr10 | 73450226 | 73450341 | 116 | *CDH23* | 284.27 |
| chr10 | 73453905 | 73454016 | 112 | *CDH23* | 136.61 |
| chr10 | 73455176 | 73455282 | 107 | *CDH23* | 229.69 |
| chr10 | 73461780 | 73461968 | 189 | *CDH23* | 243.61 |
| chr10 | 73462307 | 73462451 | 145 | *CDH23* | 262.31 |
| chr10 | 73464669 | 73464887 | 219 | *CDH23* | 399.46 |
| chr10 | 73466655 | 73466806 | 152 | *CDH23* | 291.72 |
| chr10 | 73468856 | 73468968 | 113 | *CDH23* | 310.81 |
| chr10 | 73472423 | 73472570 | 148 | *CDH23* | 344.04 |
| chr10 | 73483783 | 73483882 | 100 | *CDH23* | 315.49 |
| chr10 | 73485130 | 73485277 | 148 | *CDH23* | 354.89 |
| chr10 | 73490227 | 73490361 | 135 | *CDH23* | 316.75 |
| chr10 | 73491745 | 73492132 | 388 | *CDH23* | 339.06 |
| chr10 | 73493998 | 73494098 | 101 | *CDH23* | 342.48 |
| chr10 | 73498253 | 73498404 | 152 | *CDH23* | 204.56 |
| chr10 | 73499402 | 73499529 | 128 | *CDH23* | 176.27 |
| chr10 | 73500580 | 73500707 | 128 | *CDH23* | 224.73 |
| chr10 | 73501452 | 73501678 | 227 | *CDH23* | 230.92 |
| chr10 | 73537438 | 73537658 | 221 | *CDH23* | 131.27 |
| chr10 | 73537947 | 73538065 | 119 | *CDH23* | 149.09 |
| chr10 | 73539025 | 73539204 | 180 | *CDH23* | 125.52 |
| chr10 | 73544045 | 73544177 | 133 | *CDH23* | 142.05 |
| chr10 | 73544649 | 73544857 | 209 | *CDH23* | 199.89 |
| chr10 | 73545389 | 73545495 | 107 | *CDH23* | 186.1 |
| chr10 | 73548698 | 73548799 | 102 | *CDH23* | 445.96 |
| chr10 | 73550046 | 73550170 | 125 | *CDH23* | 47.82 |
| chr10 | 73550890 | 73551092 | 203 | *CDH23* | 81.34 |
| chr10 | 73552940 | 73553397 | 458 | *CDH23* | 355.89 |
| chr10 | 73556862 | 73556977 | 116 | *CDH23* | 170.06 |
| chr10 | 73558112 | 73558335 | 224 | *CDH23* | 173.79 |
| chr10 | 73558869 | 73559037 | 169 | *CDH23* | 211.64 |
| chr10 | 73559250 | 73559386 | 137 | *CDH23* | 206.31 |
| chr10 | 73560394 | 73560512 | 119 | *CDH23* | 214.14 |
| chr10 | 73562656 | 73562832 | 177 | *CDH23* | 289.29 |
| chr10 | 73562967 | 73563177 | 211 | *CDH23* | 274 |
| chr10 | 73565564 | 73565754 | 191 | *CDH23* | 229.28 |
| chr10 | 73565926 | 73566038 | 113 | *CDH23* | 204.51 |
| chr10 | 73567035 | 73567163 | 129 | *CDH23* | 226.12 |
| chr10 | 73567274 | 73567524 | 251 | *CDH23* | 247.92 |
| chr10 | 73567604 | 73567764 | 161 | *CDH23* | 234.38 |
| chr10 | 73569578 | 73569833 | 256 | *CDH23* | 216.51 |
| chr10 | 73570228 | 73570328 | 101 | *CDH23* | 229.89 |
| chr10 | 73571073 | 73571192 | 120 | *CDH23* | 229.61 |
| chr10 | 73571258 | 73571358 | 101 | *CDH23* | 348.12 |
| chr10 | 73571441 | 73571540 | 100 | *CDH23* | 231.83 |
| chr10 | 73571693 | 73571792 | 100 | *CDH23* | 166.08 |
| chr10 | 73572238 | 73572366 | 129 | *CDH23* | 303.67 |
| chr10 | 73572526 | 73572647 | 122 | *CDH23* | 362.28 |
| chr10 | 73573002 | 73573105 | 104 | *CDH23* | 197.04 |
| chr10 | 73574710 | 73575035 | 326 | *CDH23* | 382.75 |
| chr10 | 90750633 | 90750663 | 31 | *FAS* | 110.52 |
| chr10 | 90762785 | 90762951 | 167 | *FAS* | 138.45 |
| chr10 | 90767456 | 90767594 | 139 | *FAS* | 148.97 |
| chr10 | 90768645 | 90768754 | 110 | *FAS* | 103.63 |
| chr10 | 90770295 | 90770357 | 63 | *FAS* | 65.79 |
| chr10 | 90771755 | 90771838 | 84 | *FAS* | 73.65 |
| chr10 | 90773875 | 90774207 | 333 | *FAS* | 193.6 |
| chr10 | 102777856 | 102778053 | 198 | *PDZD7* | 130.66 |
| chr10 | 102778579 | 102778974 | 396 | *PDZD7* | 306.4 |
| chr10 | 102780356 | 102780455 | 100 | *PDZD7* | 349.93 |
| chr10 | 102781555 | 102781702 | 148 | *PDZD7* | 298.57 |
| chr10 | 102781966 | 102782142 | 177 | *PDZD7* | 314.86 |
| chr10 | 102783193 | 102783367 | 175 | *PDZD7* | 183.78 |
| chr10 | 102783685 | 102783825 | 141 | *PDZD7* | 321.36 |
| chr10 | 102789751 | 102789976 | 226 | *PDZD7* | 284.85 |
| chr11 | 2549157 | 2549248 | 92 | *KCNQ1* | 433.41 |
| chr11 | 2591857 | 2591984 | 128 | *KCNQ1* | 252.81 |
| chr11 | 2592554 | 2592633 | 80 | *KCNQ1* | 205.46 |
| chr11 | 2593242 | 2593339 | 98 | *KCNQ1* | 325.56 |
| chr11 | 2594075 | 2594216 | 142 | *KCNQ1* | 349.79 |
| chr11 | 2604664 | 2604775 | 112 | *KCNQ1* | 363.81 |
| chr11 | 2606441 | 2606537 | 97 | *KCNQ1* | 459.9 |
| chr11 | 2608799 | 2608922 | 124 | *KCNQ1* | 408.63 |
| chr11 | 2609942 | 2610084 | 143 | *KCNQ1* | 352.53 |
| chr11 | 2683190 | 2683311 | 122 | *KCNQ1* | 383.15 |
| chr11 | 2790073 | 2790149 | 77 | *KCNQ1* | 415.92 |
| chr11 | 2797189 | 2797284 | 96 | *KCNQ1* | 374.42 |
| chr11 | 2798215 | 2798262 | 48 | *KCNQ1* | 421.15 |
| chr11 | 2799205 | 2799267 | 63 | *KCNQ1* | 379.08 |
| chr11 | 2868996 | 2869233 | 238 | *KCNQ1* | 292.01 |
| chr11 | 17515860 | 17515959 | 100 | *USH1C* | 267.81 |
| chr11 | 17517116 | 17517224 | 109 | *USH1C* | 178.38 |
| chr11 | 17518283 | 17518382 | 100 | *USH1C* | 201.65 |
| chr11 | 17519709 | 17519818 | 110 | *USH1C* | 427.37 |
| chr11 | 17522598 | 17522697 | 100 | *USH1C* | 256.38 |
| chr11 | 17523006 | 17523105 | 100 | *USH1C* | 254.72 |
| chr11 | 17523457 | 17523556 | 100 | *USH1C* | 276.66 |
| chr11 | 17526170 | 17526269 | 100 | *USH1C* | 183.77 |
| chr11 | 17527377 | 17527496 | 120 | *USH1C* | 173 |
| chr11 | 17530903 | 17531385 | 483 | *USH1C* | 161.01 |
| chr11 | 17531952 | 17532068 | 117 | *USH1C* | 393.4 |
| chr11 | 17533449 | 17533601 | 153 | *USH1C* | 387.12 |
| chr11 | 17537763 | 17537862 | 100 | *USH1C* | 416.69 |
| chr11 | 17538935 | 17539034 | 100 | *USH1C* | 378.63 |
| chr11 | 17542417 | 17542541 | 125 | *USH1C* | 298.07 |
| chr11 | 17542876 | 17542975 | 100 | *USH1C* | 227.98 |
| chr11 | 17544331 | 17544473 | 143 | *USH1C* | 136.41 |
| chr11 | 17544737 | 17544836 | 100 | *USH1C* | 70.22 |
| chr11 | 17544946 | 17545045 | 100 | *USH1C* | 104.62 |
| chr11 | 17545990 | 17546090 | 101 | *USH1C* | 98.64 |
| chr11 | 17547891 | 17547991 | 101 | *USH1C* | 400.65 |
| chr11 | 17548279 | 17548378 | 100 | *USH1C* | 500.08 |
| chr11 | 17548526 | 17548625 | 100 | *USH1C* | 145.62 |
| chr11 | 17548770 | 17548878 | 109 | *USH1C* | 279.98 |
| chr11 | 17552701 | 17552839 | 139 | *USH1C* | 199.95 |
| chr11 | 17552946 | 17553089 | 144 | *USH1C* | 184.62 |
| chr11 | 17554786 | 17554885 | 100 | *USH1C* | 87.06 |
| chr11 | 17565787 | 17565886 | 100 | *USH1C* | 117.37 |
| chr11 | 67351265 | 67351365 | 101 | *GSTP1* | 155.33 |
| chr11 | 67351573 | 67351672 | 100 | *GSTP1* | 232.46 |
| chr11 | 67351935 | 67352041 | 107 | *GSTP1* | 174.7 |
| chr11 | 67352150 | 67352249 | 100 | *GSTP1* | 229.53 |
| chr11 | 67352609 | 67352712 | 104 | *GSTP1* | 211.02 |
| chr11 | 67353575 | 67353682 | 108 | *GSTP1* | 363.2 |
| chr11 | 67353860 | 67354048 | 189 | *GSTP1* | 373.52 |
| chr11 | 69625073 | 69625468 | 396 | *FGF3* | 196.96 |
| chr11 | 69631088 | 69631191 | 104 | *FGF3* | 273.53 |
| chr11 | 69633482 | 69633701 | 220 | *FGF3* | 61.22 |
| chr11 | 71800121 | 71800220 | 100 | *LRTOMT* | 150.99 |
| chr11 | 71804542 | 71804747 | 206 | *LRTOMT* | 204.39 |
| chr11 | 71805994 | 71806142 | 149 | *LRTOMT* | 146.66 |
| chr11 | 71806425 | 71806566 | 142 | *LRTOMT* | 206.05 |
| chr11 | 71807739 | 71807838 | 100 | *LRTOMT* | 165.75 |
| chr11 | 71815984 | 71816084 | 101 | *LRTOMT* | 146.14 |
| chr11 | 71816982 | 71817256 | 275 | *LRTOMT* | 152.19 |
| chr11 | 71818969 | 71819165 | 197 | *LRTOMT* | 160.6 |
| chr11 | 71819651 | 71819971 | 321 | *LRTOMT* | 126.12 |
| chr11 | 76841640 | 76841740 | 101 | *MYO7A* | 295.62 |
| chr11 | 76853756 | 76853868 | 113 | *MYO7A* | 461.35 |
| chr11 | 76866954 | 76867137 | 184 | *MYO7A* | 361.93 |
| chr11 | 76867707 | 76867827 | 121 | *MYO7A* | 273.35 |
| chr11 | 76867909 | 76868050 | 142 | *MYO7A* | 315.6 |
| chr11 | 76868326 | 76868438 | 113 | *MYO7A* | 300.27 |
| chr11 | 76869324 | 76869476 | 153 | *MYO7A* | 94.59 |
| chr11 | 76870482 | 76870581 | 100 | *MYO7A* | 56.01 |
| chr11 | 76871210 | 76871328 | 119 | *MYO7A* | 130.55 |
| chr11 | 76872020 | 76872161 | 142 | *MYO7A* | 126.13 |
| chr11 | 76873167 | 76873376 | 210 | *MYO7A* | 128.65 |
| chr11 | 76873900 | 76874034 | 135 | *MYO7A* | 105.44 |
| chr11 | 76877103 | 76877208 | 106 | *MYO7A* | 72.83 |
| chr11 | 76883795 | 76883931 | 137 | *MYO7A* | 47.39 |
| chr11 | 76885803 | 76885960 | 158 | *MYO7A* | 89.47 |
| chr11 | 76886415 | 76886514 | 100 | *MYO7A* | 71.36 |
| chr11 | 76888593 | 76888692 | 100 | *MYO7A* | 114.53 |
| chr11 | 76890084 | 76890183 | 100 | *MYO7A* | 190.77 |
| chr11 | 76890782 | 76890999 | 218 | *MYO7A* | 94.17 |
| chr11 | 76891421 | 76891527 | 107 | *MYO7A* | 99.73 |
| chr11 | 76892427 | 76892635 | 209 | *MYO7A* | 146.48 |
| chr11 | 76892998 | 76893200 | 203 | *MYO7A* | 175.68 |
| chr11 | 76893470 | 76893645 | 176 | *MYO7A* | 159.68 |
| chr11 | 76894108 | 76894208 | 101 | *MYO7A* | 97.5 |
| chr11 | 76895634 | 76895794 | 161 | *MYO7A* | 160.27 |
| chr11 | 76900390 | 76900515 | 126 | *MYO7A* | 351.9 |
| chr11 | 76901066 | 76901184 | 119 | *MYO7A* | 202.46 |
| chr11 | 76901743 | 76901915 | 173 | *MYO7A* | 199.39 |
| chr11 | 76903097 | 76903323 | 227 | *MYO7A* | 407.39 |
| chr11 | 76905400 | 76905569 | 170 | *MYO7A* | 408.69 |
| chr11 | 76908527 | 76908643 | 117 | *MYO7A* | 248.16 |
| chr11 | 76909541 | 76909666 | 126 | *MYO7A* | 216.29 |
| chr11 | 76910581 | 76910863 | 283 | *MYO7A* | 393.9 |
| chr11 | 76912494 | 76912683 | 190 | *MYO7A* | 227.61 |
| chr11 | 76913346 | 76913469 | 124 | *MYO7A* | 193.85 |
| chr11 | 76914106 | 76914262 | 157 | *MYO7A* | 92.71 |
| chr11 | 76915121 | 76915274 | 154 | *MYO7A* | 181.77 |
| chr11 | 76916508 | 76916662 | 155 | *MYO7A* | 291.08 |
| chr11 | 76917143 | 76917247 | 105 | *MYO7A* | 258.01 |
| chr11 | 76918335 | 76918447 | 113 | *MYO7A* | 398.46 |
| chr11 | 76919469 | 76919569 | 101 | *MYO7A* | 321.84 |
| chr11 | 76919743 | 76919848 | 106 | *MYO7A* | 347.4 |
| chr11 | 76922198 | 76922382 | 185 | *MYO7A* | 261.95 |
| chr11 | 76922867 | 76922982 | 116 | *MYO7A* | 326.22 |
| chr11 | 76923989 | 76924089 | 101 | *MYO7A* | 387.19 |
| chr11 | 76924906 | 76925024 | 119 | *MYO7A* | 503.34 |
| chr11 | 76925647 | 76925747 | 101 | *MYO7A* | 456.2 |
| chr11 | 110102594 | 110102758 | 165 | *RDX* | 226.24 |
| chr11 | 110103962 | 110104204 | 243 | *RDX* | 338.53 |
| chr11 | 110106820 | 110106920 | 101 | *RDX* | 286.75 |
| chr11 | 110108217 | 110108377 | 161 | *RDX* | 352.2 |
| chr11 | 110118428 | 110118558 | 131 | *RDX* | 214.36 |
| chr11 | 110124671 | 110124834 | 164 | *RDX* | 221.9 |
| chr11 | 110126012 | 110126112 | 101 | *RDX* | 185.69 |
| chr11 | 110128492 | 110128638 | 147 | *RDX* | 193.11 |
| chr11 | 110128823 | 110128922 | 100 | *RDX* | 175.04 |
| chr11 | 110134685 | 110134959 | 275 | *RDX* | 139.69 |
| chr11 | 110135502 | 110135601 | 100 | *RDX* | 125 |
| chr11 | 110143253 | 110143352 | 100 | *RDX* | 310.78 |
| chr11 | 110150362 | 110150461 | 100 | *RDX* | 121.06 |
| chr11 | 113558939 | 113559039 | 101 | *TMPRSS5* | 334.56 |
| chr11 | 113560487 | 113560639 | 153 | *TMPRSS5* | 159.34 |
| chr11 | 113560953 | 113561095 | 143 | *TMPRSS5* | 141.56 |
| chr11 | 113561563 | 113561661 | 99 | *TMPRSS5* | 361.63 |
| chr11 | 113563793 | 113563971 | 179 | *TMPRSS5* | 298.09 |
| chr11 | 113565200 | 113565362 | 163 | *TMPRSS5* | 192.37 |
| chr11 | 113566094 | 113566193 | 100 | *TMPRSS5* | 197.19 |
| chr11 | 113567580 | 113567693 | 114 | *TMPRSS5* | 274.68 |
| chr11 | 113568005 | 113568140 | 136 | *TMPRSS5* | 242.53 |
| chr11 | 113569627 | 113569749 | 123 | *TMPRSS5* | 344.76 |
| chr11 | 113570317 | 113570415 | 99 | *TMPRSS5* | 434.61 |
| chr11 | 113570788 | 113570890 | 103 | *TMPRSS5* | 411.46 |
| chr11 | 113576895 | 113576995 | 101 | *TMPRSS5* | 381.03 |
| chr11 | 120973357 | 120973456 | 100 | *TECTA* | 193.54 |
| chr11 | 120976540 | 120976673 | 134 | *TECTA* | 368.01 |
| chr11 | 120979920 | 120980207 | 288 | *TECTA* | 343.17 |
| chr11 | 120983781 | 120983918 | 138 | *TECTA* | 239.94 |
| chr11 | 120984262 | 120984427 | 166 | *TECTA* | 218.17 |
| chr11 | 120989015 | 120989427 | 413 | *TECTA* | 328.11 |
| chr11 | 120996011 | 120996581 | 571 | *TECTA* | 272.31 |
| chr11 | 120998461 | 120999053 | 593 | *TECTA* | 264.77 |
| chr11 | 121000347 | 121000920 | 574 | *TECTA* | 341.55 |
| chr11 | 121008130 | 121008731 | 602 | *TECTA* | 246.39 |
| chr11 | 121016264 | 121016825 | 562 | *TECTA* | 316.64 |
| chr11 | 121023590 | 121023789 | 200 | *TECTA* | 372.86 |
| chr11 | 121028550 | 121028933 | 384 | *TECTA* | 300.82 |
| chr11 | 121030844 | 121031130 | 287 | *TECTA* | 264.13 |
| chr11 | 121032784 | 121033079 | 296 | *TECTA* | 342.87 |
| chr11 | 121035982 | 121036092 | 111 | *TECTA* | 175.09 |
| chr11 | 121037287 | 121037489 | 203 | *TECTA* | 169.18 |
| chr11 | 121038763 | 121038926 | 164 | *TECTA* | 283.36 |
| chr11 | 121039386 | 121039634 | 249 | *TECTA* | 268.26 |
| chr11 | 121058541 | 121058703 | 163 | *TECTA* | 211.52 |
| chr11 | 121059783 | 121059882 | 100 | *TECTA* | 199.88 |
| chr11 | 121060473 | 121060589 | 117 | *TECTA* | 227.29 |
| chr11 | 121061415 | 121061515 | 101 | *TECTA* | 207.34 |
| chr12 | 51489023 | 51489061 | 39 | *TFCP2* | 230.26 |
| chr12 | 51489771 | 51489823 | 53 | *TFCP2* | 53.19 |
| chr12 | 51492558 | 51492701 | 144 | *TFCP2* | 198.31 |
| chr12 | 51493437 | 51493562 | 126 | *TFCP2* | 248.55 |
| chr12 | 51495717 | 51495808 | 92 | *TFCP2* | 126.4 |
| chr12 | 51497452 | 51497546 | 95 | *TFCP2* | 292.38 |
| chr12 | 51497937 | 51497986 | 50 | *TFCP2* | 264.58 |
| chr12 | 51500307 | 51500396 | 90 | *TFCP2* | 183.68 |
| chr12 | 51501018 | 51501129 | 112 | *TFCP2* | 185.35 |
| chr12 | 51502903 | 51503056 | 154 | *TFCP2* | 322.06 |
| chr12 | 51504659 | 51504766 | 108 | *TFCP2* | 322.51 |
| chr12 | 51510097 | 51510203 | 107 | *TFCP2* | 336.17 |
| chr12 | 51511453 | 51511530 | 78 | *TFCP2* | 100.45 |
| chr12 | 51512403 | 51512555 | 153 | *TFCP2* | 204.44 |
| chr12 | 51566083 | 51566205 | 123 | *TFCP2* | 315.17 |
| chr12 | 57422539 | 57422665 | 127 | *MYO1A* | 153.35 |
| chr12 | 57422916 | 57423043 | 128 | *MYO1A* | 146.66 |
| chr12 | 57423219 | 57423371 | 153 | *MYO1A* | 126.83 |
| chr12 | 57423524 | 57423656 | 133 | *MYO1A* | 124.93 |
| chr12 | 57423994 | 57424100 | 107 | *MYO1A* | 115.41 |
| chr12 | 57424824 | 57424958 | 135 | *MYO1A* | 101.42 |
| chr12 | 57430079 | 57430178 | 100 | *MYO1A* | 58.04 |
| chr12 | 57430541 | 57430640 | 100 | *MYO1A* | 83.77 |
| chr12 | 57430726 | 57430875 | 150 | *MYO1A* | 100.38 |
| chr12 | 57431329 | 57431428 | 100 | *MYO1A* | 80.46 |
| chr12 | 57431653 | 57431853 | 201 | *MYO1A* | 133.46 |
| chr12 | 57432196 | 57432422 | 227 | *MYO1A* | 216.97 |
| chr12 | 57432593 | 57432793 | 201 | *MYO1A* | 210.39 |
| chr12 | 57432978 | 57433077 | 100 | *MYO1A* | 59.1 |
| chr12 | 57434968 | 57435072 | 105 | *MYO1A* | 70.19 |
| chr12 | 57435199 | 57435298 | 100 | *MYO1A* | 57.53 |
| chr12 | 57436849 | 57436949 | 101 | *MYO1A* | 126.53 |
| chr12 | 57437024 | 57437142 | 119 | *MYO1A* | 144.67 |
| chr12 | 57437640 | 57437787 | 148 | *MYO1A* | 153.97 |
| chr12 | 57437890 | 57437993 | 104 | *MYO1A* | 61.54 |
| chr12 | 57440336 | 57440434 | 99 | *MYO1A* | 173.97 |
| chr12 | 57440629 | 57440728 | 100 | *MYO1A* | 293.74 |
| chr12 | 57440819 | 57440918 | 100 | *MYO1A* | 271.38 |
| chr12 | 57441087 | 57441191 | 105 | *MYO1A* | 239.33 |
| chr12 | 57441408 | 57441508 | 101 | *MYO1A* | 283.72 |
| chr12 | 57441773 | 57441888 | 116 | *MYO1A* | 123.43 |
| chr12 | 57441994 | 57442107 | 114 | *MYO1A* | 133.63 |
| chr12 | 65672548 | 65672645 | 98 | *MSRB3* | 309.7 |
| chr12 | 65702359 | 65702435 | 77 | *MSRB3* | 419.45 |
| chr12 | 65720605 | 65720714 | 110 | *MSRB3* | 328.04 |
| chr12 | 65722305 | 65722383 | 79 | *MSRB3* | 276.94 |
| chr12 | 65762777 | 65762806 | 30 | *MSRB3* | 169.8 |
| chr12 | 65847507 | 65847605 | 99 | *MSRB3* | 380.52 |
| chr12 | 65856934 | 65857102 | 169 | *MSRB3* | 326.34 |
| chr12 | 80849272 | 80849842 | 571 | *PTPRQ* | 258.14 |
| chr12 | 80878211 | 80878383 | 173 | *PTPRQ* | 231.77 |
| chr12 | 96368009 | 96368150 | 142 | *HAL* | 165.84 |
| chr12 | 96370206 | 96370276 | 71 | *HAL* | 133.82 |
| chr12 | 96370375 | 96370484 | 110 | *HAL* | 142.29 |
| chr12 | 96371721 | 96371856 | 136 | *HAL* | 149.1 |
| chr12 | 96374333 | 96374499 | 167 | *HAL* | 170.07 |
| chr12 | 96374575 | 96374641 | 67 | *HAL* | 121.66 |
| chr12 | 96377688 | 96377769 | 82 | *HAL* | 80.11 |
| chr12 | 96379685 | 96379744 | 60 | *HAL* | 109.68 |
| chr12 | 96379842 | 96379938 | 97 | *HAL* | 133.42 |
| chr12 | 96380844 | 96380992 | 149 | *HAL* | 146.66 |
| chr12 | 96381970 | 96382018 | 49 | *HAL* | 19.08 |
| chr12 | 96384170 | 96384310 | 141 | *HAL* | 205.87 |
| chr12 | 96386457 | 96386583 | 127 | *HAL* | 174.98 |
| chr12 | 96387228 | 96387266 | 39 | *HAL* | 252.36 |
| chr12 | 96387549 | 96387616 | 68 | *HAL* | 161.46 |
| chr12 | 96387703 | 96387776 | 74 | *HAL* | 166.89 |
| chr12 | 96387867 | 96387942 | 76 | *HAL* | 237.68 |
| chr12 | 96388575 | 96388603 | 29 | *HAL* | 187.83 |
| chr12 | 96388710 | 96388771 | 62 | *HAL* | 231.77 |
| chr12 | 96389441 | 96389688 | 248 | *HAL* | 225.76 |
| chr12 | 100751170 | 100751270 | 101 | *SLC17A8* | 363.7 |
| chr12 | 100774479 | 100774731 | 253 | *SLC17A8* | 318.08 |
| chr12 | 100784779 | 100784897 | 119 | *SLC17A8* | 303.58 |
| chr12 | 100787147 | 100787261 | 115 | *SLC17A8* | 374.27 |
| chr12 | 100790102 | 100790201 | 100 | *SLC17A8* | 196.4 |
| chr12 | 100795548 | 100795648 | 101 | *SLC17A8* | 133.56 |
| chr12 | 100796118 | 100796257 | 140 | *SLC17A8* | 91.79 |
| chr12 | 100796374 | 100796523 | 150 | *SLC17A8* | 87.75 |
| chr12 | 100797816 | 100797948 | 133 | *SLC17A8* | 278.19 |
| chr12 | 100806548 | 100806658 | 111 | *SLC17A8* | 301.88 |
| chr12 | 100811807 | 100811934 | 128 | *SLC17A8* | 390.21 |
| chr12 | 100813593 | 100813937 | 345 | *SLC17A8* | 214.26 |
| chr12 | 122692927 | 122693124 | 198 | *DIABLO* | 468.8 |
| chr12 | 122701034 | 122701131 | 98 | *DIABLO* | 256.83 |
| chr12 | 122701305 | 122701416 | 112 | *DIABLO* | 330.21 |
| chr12 | 122702812 | 122702944 | 133 | *DIABLO* | 388.92 |
| chr12 | 122709058 | 122709191 | 134 | *DIABLO* | 373.07 |
| chr12 | 122710511 | 122710561 | 51 | *DIABLO* | 426.22 |
| chr13 | 20761430 | 20772114 | 10685 | *GJB2* | 249.74 |
| chr13 | 20796834 | 20797619 | 786 | *GJB6* | 267.77 |
| chr13 | 78472334 | 78472469 | 136 | *EDNRB* | 96 |
| chr13 | 78473993 | 78474102 | 110 | *EDNRB* | 51.26 |
| chr13 | 78474655 | 78474789 | 135 | *EDNRB* | 68.58 |
| chr13 | 78475192 | 78475342 | 151 | *EDNRB* | 32.94 |
| chr13 | 78477290 | 78477495 | 206 | *EDNRB* | 76.82 |
| chr13 | 78477629 | 78477742 | 114 | *EDNRB* | 54.4 |
| chr13 | 78492225 | 78492708 | 484 | *EDNRB* | 136.39 |
| chr14 | 31344112 | 31344211 | 100 | *COCH* | 405.47 |
| chr14 | 31344236 | 31344335 | 100 | *COCH* | 379.76 |
| chr14 | 31346778 | 31346934 | 157 | *COCH* | 124.75 |
| chr14 | 31348017 | 31348150 | 134 | *COCH* | 93.01 |
| chr14 | 31348611 | 31348710 | 100 | *COCH* | 129.17 |
| chr14 | 31349631 | 31349730 | 100 | *COCH* | 154.87 |
| chr14 | 31349793 | 31349940 | 148 | *COCH* | 153.22 |
| chr14 | 31353759 | 31353862 | 104 | *COCH* | 55.37 |
| chr14 | 31354600 | 31354826 | 227 | *COCH* | 139.08 |
| chr14 | 31355002 | 31355518 | 517 | *COCH* | 177.85 |
| chr14 | 31358822 | 31358997 | 176 | *COCH* | 108.41 |
| chr14 | 61113000 | 61113295 | 296 | *SIX1* | 245.4 |
| chr14 | 61115347 | 61115907 | 561 | *SIX1* | 179.1 |
| chr14 | 76905697 | 76906093 | 397 | *ESRRB* | 356.35 |
| chr14 | 76928888 | 76929004 | 117 | *ESRRB* | 336.47 |
| chr14 | 76948359 | 76948469 | 111 | *ESRRB* | 262.39 |
| chr14 | 76948941 | 76949102 | 162 | *ESRRB* | 242.94 |
| chr14 | 76957790 | 76958059 | 270 | *ESRRB* | 387.21 |
| chr14 | 76964557 | 76964795 | 239 | *ESRRB* | 396.32 |
| chr14 | 76966176 | 76966409 | 234 | *ESRRB* | 188.29 |
| chr14 | 76967002 | 76967101 | 100 | *ESRRB* | 186.38 |
| chr15 | 43891864 | 43891964 | 101 | *STRC* | 165.91 |
| chr15 | 43892158 | 43892305 | 148 | *STRC* | 159.56 |
| chr15 | 43892393 | 43892492 | 100 | *STRC* | 135.4 |
| chr15 | 43892732 | 43892880 | 149 | *STRC* | 115.5 |
| chr15 | 43893070 | 43893212 | 143 | *STRC* | 105.94 |
| chr15 | 43893594 | 43893749 | 156 | *STRC* | 83.59 |
| chr15 | 43895440 | 43895609 | 170 | *STRC* | 80.3 |
| chr15 | 43896194 | 43896350 | 157 | *STRC* | 47.24 |
| chr15 | 43896554 | 43896654 | 101 | *STRC* | 67.15 |
| chr15 | 43896848 | 43897044 | 197 | *STRC* | 88.2 |
| chr15 | 43897462 | 43897597 | 136 | *STRC* | 24.95 |
| chr15 | 43900061 | 43900173 | 113 | *STRC* | 46.12 |
| chr15 | 43900278 | 43900401 | 124 | *STRC* | 54.39 |
| chr15 | 43901454 | 43901553 | 100 | *STRC* | 24.48 |
| chr15 | 43902510 | 43902635 | 126 | *STRC* | 55.23 |
| chr15 | 43903100 | 43903199 | 100 | *STRC* | 118.28 |
| chr15 | 43903346 | 43903513 | 168 | *STRC* | 145.32 |
| chr15 | 43903667 | 43903766 | 100 | *STRC* | 117.16 |
| chr15 | 43904040 | 43904222 | 183 | *STRC* | 122.9 |
| chr15 | 43904575 | 43904707 | 133 | *STRC* | 199.45 |
| chr15 | 43904989 | 43905104 | 116 | *STRC* | 173.03 |
| chr15 | 43905243 | 43905429 | 187 | *STRC* | 125.17 |
| chr15 | 43906085 | 43906251 | 167 | *STRC* | 201.05 |
| chr15 | 43906394 | 43906510 | 117 | *STRC* | 232.57 |
| chr15 | 43906594 | 43906693 | 100 | *STRC* | 213.58 |
| chr15 | 43907631 | 43908888 | 1258 | *STRC* | 47.79 |
| chr15 | 43909619 | 43909718 | 100 | *STRC* | 192.9 |
| chr15 | 43909769 | 43910554 | 786 | *STRC* | 209.08 |
| chr15 | 43910839 | 43910938 | 100 | *STRC* | 96.72 |
| chr15 | 43922865 | 43922964 | 100 | *CATSPER2* | 125.96 |
| chr15 | 43924397 | 43924561 | 165 | *CATSPER2* | 149.84 |
| chr15 | 43924915 | 43925132 | 218 | *CATSPER2* | 134.83 |
| chr15 | 43927537 | 43927636 | 100 | *CATSPER2* | 73.7 |
| chr15 | 43927925 | 43928024 | 100 | *CATSPER2* | 77.27 |
| chr15 | 43928239 | 43928417 | 179 | *CATSPER2* | 104.08 |
| chr15 | 43931102 | 43931226 | 125 | *CATSPER2* | 169.54 |
| chr15 | 43931841 | 43931996 | 156 | *CATSPER2* | 77.72 |
| chr15 | 43932484 | 43932694 | 211 | *CATSPER2* | 78.78 |
| chr15 | 43939233 | 43939332 | 100 | *CATSPER2* | 100.08 |
| chr15 | 43939492 | 43939665 | 174 | *CATSPER2* | 115.93 |
| chr15 | 43940115 | 43940259 | 145 | *CATSPER2* | 92.74 |
| chr15 | 81166220 | 81166314 | 95 | *KIAA1199* | 411.26 |
| chr15 | 81171061 | 81171208 | 148 | *KIAA1199* | 399.06 |
| chr15 | 81172056 | 81172195 | 140 | *KIAA1199* | 444.47 |
| chr15 | 81173240 | 81173477 | 238 | *KIAA1199* | 391.5 |
| chr15 | 81176515 | 81176695 | 181 | *KIAA1199* | 401.02 |
| chr15 | 81180034 | 81180105 | 72 | *KIAA1199* | 287.74 |
| chr15 | 81181041 | 81181137 | 97 | *KIAA1199* | 220.46 |
| chr15 | 81181811 | 81181933 | 123 | *KIAA1199* | 265.93 |
| chr15 | 81187330 | 81187463 | 134 | *KIAA1199* | 403.2 |
| chr15 | 81188209 | 81188401 | 193 | *KIAA1199* | 418.26 |
| chr15 | 81199003 | 81199179 | 177 | *KIAA1199* | 436.89 |
| chr15 | 81201437 | 81201647 | 211 | *KIAA1199* | 402.16 |
| chr15 | 81212434 | 81212640 | 207 | *KIAA1199* | 424.97 |
| chr15 | 81213372 | 81213442 | 71 | *KIAA1199* | 387.08 |
| chr15 | 81214349 | 81214478 | 130 | *KIAA1199* | 461.86 |
| chr15 | 81216961 | 81217047 | 87 | *KIAA1199* | 383.94 |
| chr15 | 81217964 | 81218096 | 133 | *KIAA1199* | 430.38 |
| chr15 | 81221242 | 81221278 | 37 | *KIAA1199* | 316.43 |
| chr15 | 81221359 | 81221515 | 157 | *KIAA1199* | 435.86 |
| chr15 | 81224199 | 81224380 | 182 | *KIAA1199* | 343.86 |
| chr15 | 81225585 | 81225801 | 217 | *KIAA1199* | 396.33 |
| chr15 | 81229014 | 81229226 | 213 | *KIAA1199* | 395.32 |
| chr15 | 81230134 | 81230320 | 187 | *KIAA1199* | 414.12 |
| chr15 | 81234189 | 81234394 | 206 | *KIAA1199* | 313.8 |
| chr15 | 81234591 | 81234678 | 88 | *KIAA1199* | 273.25 |
| chr15 | 81235285 | 81235443 | 159 | *KIAA1199* | 417.74 |
| chr15 | 81239305 | 81239406 | 102 | *KIAA1199* | 344.36 |
| chr15 | 81241137 | 81241265 | 129 | *KIAA1199* | 346.05 |
| chr16 | 21270085 | 21270184 | 100 | *CRYM* | 151.59 |
| chr16 | 21272567 | 21272667 | 101 | *CRYM* | 135.82 |
| chr16 | 21273358 | 21273479 | 122 | *CRYM* | 140.17 |
| chr16 | 21278875 | 21279058 | 184 | *CRYM* | 182.7 |
| chr16 | 21281111 | 21281212 | 102 | *CRYM* | 40.65 |
| chr16 | 21286835 | 21286934 | 100 | *CRYM* | 55.46 |
| chr16 | 21288752 | 21288905 | 154 | *CRYM* | 137.12 |
| chr16 | 21289403 | 21289572 | 170 | *CRYM* | 137.48 |
| chr16 | 21689831 | 21689931 | 101 | *OTOA* | 178.46 |
| chr16 | 21690201 | 21690300 | 100 | *OTOA* | 250.89 |
| chr16 | 21690323 | 21690422 | 100 | *OTOA* | 263.74 |
| chr16 | 21690469 | 21690568 | 100 | *OTOA* | 261.48 |
| chr16 | 21693053 | 21693152 | 100 | *OTOA* | 386.99 |
| chr16 | 21695809 | 21695909 | 101 | *OTOA* | 253.29 |
| chr16 | 21696551 | 21696682 | 132 | *OTOA* | 376.53 |
| chr16 | 21698734 | 21698969 | 236 | *OTOA* | 316.86 |
| chr16 | 21702905 | 21703008 | 104 | *OTOA* | 392.77 |
| chr16 | 21709096 | 21709196 | 101 | *OTOA* | 351.64 |
| chr16 | 21712209 | 21712348 | 140 | *OTOA* | 328.31 |
| chr16 | 21716305 | 21716404 | 100 | *OTOA* | 94.7 |
| chr16 | 21716490 | 21716613 | 124 | *OTOA* | 415.16 |
| chr16 | 21721209 | 21721424 | 216 | *OTOA* | 362.89 |
| chr16 | 21726306 | 21726473 | 168 | *OTOA* | 488.42 |
| chr16 | 21728228 | 21728368 | 141 | *OTOA* | 301.04 |
| chr16 | 21730434 | 21730533 | 100 | *OTOA* | 380.5 |
| chr16 | 21730708 | 21730825 | 118 | *OTOA* | 325.56 |
| chr16 | 21734213 | 21734312 | 100 | *OTOA* | 362 |
| chr16 | 21737844 | 21737979 | 136 | *OTOA* | 329.51 |
| chr16 | 21739562 | 21739752 | 191 | *OTOA* | 355.87 |
| chr16 | 21742155 | 21742254 | 100 | *OTOA* | 366.59 |
| chr16 | 21747582 | 21747711 | 130 | *OTOA* | 186.68 |
| chr16 | 21752042 | 21752229 | 188 | *OTOA* | 692.18 |
| chr16 | 21756202 | 21756357 | 156 | *OTOA* | 486.74 |
| chr16 | 21763256 | 21763398 | 143 | *OTOA* | 219.82 |
| chr16 | 21763690 | 21763826 | 137 | *OTOA* | 241.45 |
| chr16 | 21764347 | 21764446 | 100 | *OTOA* | 155.5 |
| chr16 | 21768403 | 21768598 | 196 | *OTOA* | 447.36 |
| chr16 | 21771777 | 21771876 | 100 | *OTOA* | 213.4 |
| chr17 | 1368984 | 1369083 | 100 | *MYO1C* | 96.77 |
| chr17 | 1370536 | 1370635 | 100 | *MYO1C* | 89.98 |
| chr17 | 1370773 | 1370872 | 100 | *MYO1C* | 67.76 |
| chr17 | 1371120 | 1371219 | 100 | *MYO1C* | 50.5 |
| chr17 | 1371282 | 1371417 | 136 | *MYO1C* | 82.42 |
| chr17 | 1371525 | 1371624 | 100 | *MYO1C* | 36.78 |
| chr17 | 1371694 | 1371793 | 100 | *MYO1C* | 64.58 |
| chr17 | 1372817 | 1372916 | 100 | *MYO1C* | 70.11 |
| chr17 | 1373469 | 1373628 | 160 | *MYO1C* | 55.34 |
| chr17 | 1373721 | 1373821 | 101 | *MYO1C* | 51.26 |
| chr17 | 1373900 | 1373999 | 100 | *MYO1C* | 39.61 |
| chr17 | 1374354 | 1374453 | 100 | *MYO1C* | 103.85 |
| chr17 | 1374517 | 1374630 | 114 | *MYO1C* | 96.25 |
| chr17 | 1375201 | 1375318 | 118 | *MYO1C* | 95.75 |
| chr17 | 1375417 | 1375522 | 106 | *MYO1C* | 127.51 |
| chr17 | 1377894 | 1377994 | 101 | *MYO1C* | 297.01 |
| chr17 | 1378080 | 1378179 | 100 | *MYO1C* | 188.21 |
| chr17 | 1378229 | 1378329 | 101 | *MYO1C* | 265.94 |
| chr17 | 1380795 | 1380894 | 100 | *MYO1C* | 126.97 |
| chr17 | 1381175 | 1381275 | 101 | *MYO1C* | 126.57 |
| chr17 | 1381381 | 1381486 | 106 | *MYO1C* | 168.93 |
| chr17 | 1381695 | 1381795 | 101 | *MYO1C* | 55.41 |
| chr17 | 1381910 | 1382029 | 120 | *MYO1C* | 100.5 |
| chr17 | 1382711 | 1382810 | 100 | *MYO1C* | 54.51 |
| chr17 | 1382886 | 1382999 | 114 | *MYO1C* | 49.79 |
| chr17 | 1383821 | 1383919 | 99 | *MYO1C* | 95.82 |
| chr17 | 1384000 | 1384179 | 180 | *MYO1C* | 132.9 |
| chr17 | 1385762 | 1385862 | 101 | *MYO1C* | 46.52 |
| chr17 | 1386155 | 1386353 | 199 | *MYO1C* | 76.46 |
| chr17 | 1386904 | 1387019 | 116 | *MYO1C* | 80.8 |
| chr17 | 1387442 | 1387597 | 156 | *MYO1C* | 82.16 |
| chr17 | 1395694 | 1395794 | 101 | *MYO1C* | 125.18 |
| chr17 | 15134234 | 15134397 | 164 | *PMP22* | 155.08 |
| chr17 | 15142788 | 15142928 | 141 | *PMP22* | 333.99 |
| chr17 | 15162411 | 15162510 | 100 | *PMP22* | 353.58 |
| chr17 | 15163956 | 15164055 | 100 | *PMP22* | 363.02 |
| chr17 | 18021799 | 18024100 | 2302 | *MYO15A* | 270.67 |
| chr17 | 18024179 | 18024600 | 422 | *MYO15A* | 128.08 |
| chr17 | 18024639 | 18025850 | 1212 | *MYO15A* | 127.99 |
| chr17 | 18027788 | 18027888 | 101 | *MYO15A* | 79.15 |
| chr17 | 18028464 | 18028563 | 100 | *MYO15A* | 64.98 |
| chr17 | 18029661 | 18029770 | 110 | *MYO15A* | 88.35 |
| chr17 | 18030092 | 18030192 | 101 | *MYO15A* | 82.24 |
| chr17 | 18030386 | 18030486 | 101 | *MYO15A* | 145.76 |
| chr17 | 18034072 | 18034171 | 100 | *MYO15A* | 42.52 |
| chr17 | 18034552 | 18034656 | 105 | *MYO15A* | 149.26 |
| chr17 | 18034764 | 18034863 | 100 | *MYO15A* | 137.01 |
| chr17 | 18035767 | 18035880 | 114 | *MYO15A* | 90.22 |
| chr17 | 18036539 | 18036700 | 162 | *MYO15A* | 108.08 |
| chr17 | 18039025 | 18039138 | 114 | *MYO15A* | 74.74 |
| chr17 | 18039710 | 18039810 | 101 | *MYO15A* | 117.36 |
| chr17 | 18039877 | 18040000 | 124 | *MYO15A* | 107.62 |
| chr17 | 18040896 | 18040995 | 100 | *MYO15A* | 77.68 |
| chr17 | 18041429 | 18041560 | 132 | *MYO15A* | 67.3 |
| chr17 | 18042125 | 18042250 | 126 | *MYO15A* | 127.48 |
| chr17 | 18042837 | 18042936 | 100 | *MYO15A* | 91.76 |
| chr17 | 18043831 | 18043979 | 149 | *MYO15A* | 121.3 |
| chr17 | 18044074 | 18044173 | 100 | *MYO15A* | 136.38 |
| chr17 | 18044333 | 18044457 | 125 | *MYO15A* | 132.53 |
| chr17 | 18044967 | 18045084 | 118 | *MYO15A* | 102.18 |
| chr17 | 18045393 | 18045568 | 176 | *MYO15A* | 69.69 |
| chr17 | 18046062 | 18046162 | 101 | *MYO15A* | 107.01 |
| chr17 | 18046857 | 18046956 | 100 | *MYO15A* | 89.97 |
| chr17 | 18047020 | 18047119 | 100 | *MYO15A* | 106.24 |
| chr17 | 18047184 | 18047314 | 131 | *MYO15A* | 91.15 |
| chr17 | 18047809 | 18047908 | 100 | *MYO15A* | 68.06 |
| chr17 | 18049186 | 18049421 | 236 | *MYO15A* | 119.18 |
| chr17 | 18051343 | 18051524 | 182 | *MYO15A* | 100.2 |
| chr17 | 18051796 | 18051896 | 101 | *MYO15A* | 126.42 |
| chr17 | 18052075 | 18052266 | 192 | *MYO15A* | 112.73 |
| chr17 | 18052530 | 18052690 | 161 | *MYO15A* | 139.01 |
| chr17 | 18052797 | 18052897 | 101 | *MYO15A* | 100.15 |
| chr17 | 18053743 | 18053857 | 115 | *MYO15A* | 93.35 |
| chr17 | 18053996 | 18054095 | 100 | *MYO15A* | 85.18 |
| chr17 | 18054139 | 18054238 | 100 | *MYO15A* | 76.12 |
| chr17 | 18054424 | 18054604 | 181 | *MYO15A* | 96.44 |
| chr17 | 18054709 | 18054841 | 133 | *MYO15A* | 97.58 |
| chr17 | 18055160 | 18055265 | 106 | *MYO15A* | 107.37 |
| chr17 | 18055412 | 18055512 | 101 | *MYO15A* | 142.86 |
| chr17 | 18057089 | 18057210 | 122 | *MYO15A* | 73.84 |
| chr17 | 18057425 | 18057524 | 100 | *MYO15A* | 103.65 |
| chr17 | 18057982 | 18058081 | 100 | *MYO15A* | 107.98 |
| chr17 | 18058424 | 18058539 | 116 | *MYO15A* | 84.39 |
| chr17 | 18058628 | 18058746 | 119 | *MYO15A* | 69.88 |
| chr17 | 18059509 | 18059650 | 142 | *MYO15A* | 96.61 |
| chr17 | 18060268 | 18060379 | 112 | *MYO15A* | 84.16 |
| chr17 | 18060457 | 18060557 | 101 | *MYO15A* | 128.88 |
| chr17 | 18061036 | 18061214 | 179 | *MYO15A* | 74.26 |
| chr17 | 18061837 | 18061952 | 116 | *MYO15A* | 101.98 |
| chr17 | 18062225 | 18062325 | 101 | *MYO15A* | 125.72 |
| chr17 | 18062575 | 18062675 | 101 | *MYO15A* | 192.26 |
| chr17 | 18062907 | 18063006 | 100 | *MYO15A* | 76.58 |
| chr17 | 18063240 | 18063340 | 101 | *MYO15A* | 110.07 |
| chr17 | 18064631 | 18064761 | 131 | *MYO15A* | 77.01 |
| chr17 | 18065896 | 18065996 | 101 | *MYO15A* | 74.61 |
| chr17 | 18066547 | 18066646 | 100 | *MYO15A* | 91.09 |
| chr17 | 18067054 | 18067154 | 101 | *MYO15A* | 59.69 |
| chr17 | 18069675 | 18069835 | 161 | *MYO15A* | 99.29 |
| chr17 | 18070904 | 18071037 | 134 | *MYO15A* | 66.13 |
| chr17 | 18074952 | 18075085 | 134 | *MYO15A* | 77.97 |
| chr17 | 18075471 | 18075604 | 134 | *MYO15A* | 103 |
| chr17 | 18077095 | 18077235 | 141 | *MYO15A* | 95.36 |
| chr17 | 18082083 | 18082184 | 102 | *MYO15A* | 218.18 |
| chr17 | 72914167 | 72914171 | 5 | *USH1G* | 242.4 |
| chr17 | 72915548 | 72916766 | 1219 | *USH1G* | 146.87 |
| chr17 | 72919004 | 72919168 | 165 | *USH1G* | 273.52 |
| chr17 | 79477716 | 79477859 | 144 | *ACTG1* | 109.59 |
| chr17 | 79477953 | 79478134 | 182 | *ACTG1* | 159.79 |
| chr17 | 79478214 | 79478652 | 439 | *ACTG1* | 176.84 |
| chr17 | 79478929 | 79479168 | 240 | *ACTG1* | 208.63 |
| chr17 | 79479258 | 79479380 | 123 | *ACTG1* | 190.5 |
| chr18 | 44152048 | 44152125 | 78 | *LOXHD1* | 497.86 |
| chr19 | 3586492 | 3586678 | 187 | *GIPC3* | 245.65 |
| chr19 | 3586811 | 3586992 | 182 | *GIPC3* | 279.99 |
| chr19 | 3589440 | 3589553 | 114 | *GIPC3* | 182.89 |
| chr19 | 3589828 | 3589910 | 83 | *GIPC3* | 173.3 |
| chr19 | 3590036 | 3590188 | 153 | *GIPC3* | 197.49 |
| chr19 | 8586400 | 8586500 | 101 | *MYO1F* | 176.26 |
| chr19 | 8587261 | 8587430 | 170 | *MYO1F* | 102.89 |
| chr19 | 8587518 | 8587713 | 196 | *MYO1F* | 90.13 |
| chr19 | 8590355 | 8590454 | 100 | *MYO1F* | 340.72 |
| chr19 | 8591337 | 8591485 | 149 | *MYO1F* | 331.31 |
| chr19 | 8591673 | 8591819 | 147 | *MYO1F* | 52.9 |
| chr19 | 8592222 | 8592367 | 146 | *MYO1F* | 381.39 |
| chr19 | 8595080 | 8595249 | 170 | *MYO1F* | 205.85 |
| chr19 | 8595343 | 8595457 | 115 | *MYO1F* | 135.64 |
| chr19 | 8601136 | 8601280 | 145 | *MYO1F* | 323.21 |
| chr19 | 8601383 | 8601481 | 99 | *MYO1F* | 215.51 |
| chr19 | 8601833 | 8601939 | 107 | *MYO1F* | 366.28 |
| chr19 | 8604822 | 8604921 | 100 | *MYO1F* | 358.17 |
| chr19 | 8606783 | 8606882 | 100 | *MYO1F* | 311.02 |
| chr19 | 8609181 | 8609348 | 168 | *MYO1F* | 223.18 |
| chr19 | 8610527 | 8610627 | 101 | *MYO1F* | 304.96 |
| chr19 | 8612913 | 8613013 | 101 | *MYO1F* | 181.29 |
| chr19 | 8613111 | 8613211 | 101 | *MYO1F* | 298.27 |
| chr19 | 8615044 | 8615240 | 197 | *MYO1F* | 235.49 |
| chr19 | 8615446 | 8615578 | 133 | *MYO1F* | 256.37 |
| chr19 | 8616624 | 8616758 | 135 | *MYO1F* | 271.38 |
| chr19 | 8616917 | 8617048 | 132 | *MYO1F* | 262.97 |
| chr19 | 8618018 | 8618117 | 100 | *MYO1F* | 337.33 |
| chr19 | 8618228 | 8618327 | 100 | *MYO1F* | 373 |
| chr19 | 8619358 | 8619458 | 101 | *MYO1F* | 334.13 |
| chr19 | 8619533 | 8619632 | 100 | *MYO1F* | 263.69 |
| chr19 | 8620543 | 8620680 | 138 | *MYO1F* | 332.4 |
| chr19 | 8642142 | 8642242 | 101 | *MYO1F* | 221.12 |
| chr19 | 45204726 | 45204763 | 38 | *CEACAM16* | 329.42 |
| chr19 | 45206618 | 45206963 | 346 | *CEACAM16* | 325.31 |
| chr19 | 45207287 | 45207566 | 280 | *CEACAM16* | 253.4 |
| chr19 | 45208859 | 45209138 | 280 | *CEACAM16* | 378.46 |
| chr19 | 45211132 | 45211459 | 328 | *CEACAM16* | 401.37 |
| chr19 | 45213767 | 45213778 | 12 | *CEACAM16* | 203.75 |
| chr19 | 45854883 | 45854983 | 101 | *ERCC2* | 99.7 |
| chr19 | 45855467 | 45855610 | 144 | *ERCC2* | 179.07 |
| chr19 | 45855764 | 45855907 | 144 | *ERCC2* | 172.66 |
| chr19 | 45855990 | 45856089 | 100 | *ERCC2* | 98.74 |
| chr19 | 45856328 | 45856427 | 100 | *ERCC2* | 69.37 |
| chr19 | 45856496 | 45856596 | 101 | *ERCC2* | 107.77 |
| chr19 | 45857988 | 45858109 | 122 | *ERCC2* | 140.57 |
| chr19 | 45858905 | 45859004 | 100 | *ERCC2* | 87.29 |
| chr19 | 45860528 | 45860629 | 102 | *ERCC2* | 217.83 |
| chr19 | 45860717 | 45860816 | 100 | *ERCC2* | 148.15 |
| chr19 | 45860873 | 45860972 | 100 | *ERCC2* | 140.1 |
| chr19 | 45862094 | 45862194 | 101 | *ERCC2* | 398.72 |
| chr19 | 45864782 | 45864900 | 119 | *ERCC2* | 139 |
| chr19 | 45867001 | 45867169 | 169 | *ERCC2* | 78.32 |
| chr19 | 45867244 | 45867377 | 134 | *ERCC2* | 56.4 |
| chr19 | 45867491 | 45867591 | 101 | *ERCC2* | 61.66 |
| chr19 | 45867682 | 45867805 | 124 | *ERCC2* | 71.56 |
| chr19 | 45868096 | 45868212 | 117 | *ERCC2* | 81.42 |
| chr19 | 45868300 | 45868416 | 117 | *ERCC2* | 138.41 |
| chr19 | 45871888 | 45872001 | 114 | *ERCC2* | 84.18 |
| chr19 | 45872170 | 45872269 | 100 | *ERCC2* | 117.95 |
| chr19 | 45872317 | 45872416 | 100 | *ERCC2* | 117.22 |
| chr19 | 45873391 | 45873490 | 100 | *ERCC2* | 143.74 |
| chr19 | 45873747 | 45873846 | 100 | *ERCC2* | 44.49 |
| chr19 | 46268758 | 46269369 | 612 | *SIX5* | 296.34 |
| chr19 | 46269607 | 46270413 | 807 | *SIX5* | 213.56 |
| chr19 | 46271299 | 46272102 | 804 | *SIX5* | 235.39 |
| chr19 | 50713623 | 50714027 | 405 | *MYH14* | 281.92 |
| chr19 | 50720872 | 50721028 | 157 | *MYH14* | 347.12 |
| chr19 | 50726304 | 50726403 | 100 | *MYH14* | 250.94 |
| chr19 | 50726504 | 50726606 | 103 | *MYH14* | 367.59 |
| chr19 | 50727373 | 50727472 | 100 | *MYH14* | 319.58 |
| chr19 | 50728838 | 50728938 | 101 | *MYH14* | 316.8 |
| chr19 | 50730142 | 50730241 | 100 | *MYH14* | 206.03 |
| chr19 | 50733782 | 50733880 | 99 | *MYH14* | 222.32 |
| chr19 | 50735187 | 50735327 | 141 | *MYH14* | 336.11 |
| chr19 | 50747497 | 50747596 | 100 | *MYH14* | 310.6 |
| chr19 | 50750261 | 50750379 | 119 | *MYH14* | 280.33 |
| chr19 | 50752244 | 50752396 | 153 | *MYH14* | 394.87 |
| chr19 | 50752907 | 50753080 | 174 | *MYH14* | 379.3 |
| chr19 | 50753772 | 50753945 | 174 | *MYH14* | 315.83 |
| chr19 | 50755896 | 50756010 | 115 | *MYH14* | 298.81 |
| chr19 | 50758477 | 50758576 | 100 | *MYH14* | 334.12 |
| chr19 | 50760556 | 50760743 | 188 | *MYH14* | 363.84 |
| chr19 | 50762401 | 50762522 | 122 | *MYH14* | 286.96 |
| chr19 | 50763888 | 50763987 | 100 | *MYH14* | 317.97 |
| chr19 | 50764732 | 50764892 | 161 | *MYH14* | 64.86 |
| chr19 | 50766569 | 50766677 | 109 | *MYH14* | 301.6 |
| chr19 | 50770134 | 50770265 | 132 | *MYH14* | 213.7 |
| chr19 | 50771418 | 50771624 | 207 | *MYH14* | 379.52 |
| chr19 | 50774666 | 50774803 | 138 | *MYH14* | 152.49 |
| chr19 | 50775106 | 50775229 | 124 | *MYH14* | 171.31 |
| chr19 | 50775817 | 50775988 | 172 | *MYH14* | 128.49 |
| chr19 | 50779248 | 50779460 | 213 | *MYH14* | 100 |
| chr19 | 50780014 | 50780158 | 145 | *MYH14* | 102.94 |
| chr19 | 50781340 | 50781546 | 207 | *MYH14* | 314.97 |
| chr19 | 50783294 | 50783398 | 105 | *MYH14* | 320.1 |
| chr19 | 50783488 | 50783640 | 153 | *MYH14* | 345.9 |
| chr19 | 50784851 | 50785099 | 249 | *MYH14* | 338.68 |
| chr19 | 50789739 | 50789951 | 213 | *MYH14* | 378.84 |
| chr19 | 50792693 | 50792905 | 213 | *MYH14* | 320.94 |
| chr19 | 50794144 | 50794305 | 162 | *MYH14* | 322.32 |
| chr19 | 50795518 | 50795646 | 129 | *MYH14* | 416.05 |
| chr19 | 50796484 | 50796584 | 101 | *MYH14* | 360.1 |
| chr19 | 50796821 | 50796944 | 124 | *MYH14* | 290.31 |
| chr19 | 50804918 | 50805126 | 209 | *MYH14* | 354.92 |
| chr19 | 50810306 | 50810414 | 109 | *MYH14* | 354.49 |
| chr19 | 50812262 | 50812434 | 173 | *MYH14* | 247.14 |
| chr19 | 50812897 | 50813047 | 151 | *MYH14* | 220.53 |
| chr20 | 3208418 | 3208517 | 100 | *SLC4A11* | 159.36 |
| chr20 | 3208905 | 3209074 | 170 | *SLC4A11* | 321.94 |
| chr20 | 3209158 | 3209353 | 196 | *SLC4A11* | 345.91 |
| chr20 | 3209484 | 3209657 | 174 | *SLC4A11* | 159.14 |
| chr20 | 3209741 | 3209909 | 169 | *SLC4A11* | 77.98 |
| chr20 | 3209992 | 3210098 | 107 | *SLC4A11* | 128.79 |
| chr20 | 3210170 | 3210422 | 253 | *SLC4A11* | 176.41 |
| chr20 | 3210820 | 3210919 | 100 | *SLC4A11* | 157.63 |
| chr20 | 3211161 | 3211293 | 133 | *SLC4A11* | 196.35 |
| chr20 | 3211378 | 3211491 | 114 | *SLC4A11* | 110.96 |
| chr20 | 3211579 | 3211704 | 126 | *SLC4A11* | 146.52 |
| chr20 | 3211792 | 3211891 | 100 | *SLC4A11* | 162.94 |
| chr20 | 3211976 | 3212194 | 219 | *SLC4A11* | 186.19 |
| chr20 | 3214160 | 3214283 | 124 | *SLC4A11* | 113.99 |
| chr20 | 3214558 | 3214657 | 100 | *SLC4A11* | 134.23 |
| chr20 | 3214729 | 3214960 | 232 | *SLC4A11* | 148.26 |
| chr20 | 3215189 | 3215288 | 100 | *SLC4A11* | 309.72 |
| chr20 | 3215388 | 3215540 | 153 | *SLC4A11* | 335.98 |
| chr20 | 3218190 | 3218325 | 136 | *SLC4A11* | 357.46 |
| chr20 | 10620146 | 10620603 | 458 | *JAG1* | 178.67 |
| chr20 | 10621431 | 10621581 | 151 | *JAG1* | 76.15 |
| chr20 | 10621761 | 10621892 | 132 | *JAG1* | 94.98 |
| chr20 | 10622108 | 10622341 | 234 | *JAG1* | 228.97 |
| chr20 | 10622431 | 10622540 | 110 | *JAG1* | 244.52 |
| chr20 | 10623136 | 10623249 | 114 | *JAG1* | 131.18 |
| chr20 | 10624419 | 10624518 | 100 | *JAG1* | 361.78 |
| chr20 | 10624969 | 10625068 | 100 | *JAG1* | 336.66 |
| chr20 | 10625511 | 10625627 | 117 | *JAG1* | 361.28 |
| chr20 | 10625791 | 10625904 | 114 | *JAG1* | 362.07 |
| chr20 | 10626004 | 10626117 | 114 | *JAG1* | 329.58 |
| chr20 | 10626619 | 10626732 | 114 | *JAG1* | 89.28 |
| chr20 | 10627587 | 10627751 | 165 | *JAG1* | 127.79 |
| chr20 | 10628608 | 10628758 | 151 | *JAG1* | 165.72 |
| chr20 | 10629197 | 10629370 | 174 | *JAG1* | 121.56 |
| chr20 | 10629683 | 10629782 | 100 | *JAG1* | 25.42 |
| chr20 | 10630170 | 10630283 | 114 | *JAG1* | 238.03 |
| chr20 | 10630895 | 10631008 | 114 | *JAG1* | 198.69 |
| chr20 | 10632229 | 10632342 | 114 | *JAG1* | 205.09 |
| chr20 | 10632779 | 10632898 | 120 | *JAG1* | 162.32 |
| chr20 | 10633116 | 10633246 | 131 | *JAG1* | 162.95 |
| chr20 | 10637027 | 10637126 | 100 | *JAG1* | 193.28 |
| chr20 | 10639116 | 10639370 | 255 | *JAG1* | 358.49 |
| chr20 | 10644587 | 10644686 | 100 | *JAG1* | 299.13 |
| chr20 | 10653349 | 10653654 | 306 | *JAG1* | 315.21 |
| chr20 | 10654088 | 10654188 | 101 | *JAG1* | 74.19 |
| chr20 | 16729047 | 16729161 | 115 | *OTOR* | 285.32 |
| chr20 | 16729512 | 16729651 | 140 | *OTOR* | 208.69 |
| chr20 | 16730548 | 16730655 | 108 | *OTOR* | 346.94 |
| chr20 | 16731702 | 16731801 | 100 | *OTOR* | 80.07 |
| chr20 | 61449838 | 61449937 | 100 | *COL9A3* | 234.7 |
| chr20 | 61450560 | 61450659 | 100 | *COL9A3* | 94.35 |
| chr20 | 61451258 | 61451357 | 100 | *COL9A3* | 175.9 |
| chr20 | 61452501 | 61452600 | 100 | *COL9A3* | 315.61 |
| chr20 | 61452821 | 61452920 | 100 | *COL9A3* | 360.25 |
| chr20 | 61453086 | 61453185 | 100 | *COL9A3* | 124.46 |
| chr20 | 61453440 | 61453539 | 100 | *COL9A3* | 193.63 |
| chr20 | 61453914 | 61454013 | 100 | *COL9A3* | 51.06 |
| chr20 | 61455776 | 61455875 | 100 | *COL9A3* | 248.82 |
| chr20 | 61456297 | 61456396 | 100 | *COL9A3* | 113.87 |
| chr20 | 61457146 | 61457245 | 100 | *COL9A3* | 303.6 |
| chr20 | 61457533 | 61457632 | 100 | *COL9A3* | 164.8 |
| chr20 | 61458096 | 61458195 | 100 | *COL9A3* | 287.01 |
| chr20 | 61458570 | 61458669 | 100 | *COL9A3* | 235.96 |
| chr20 | 61459252 | 61459351 | 100 | *COL9A3* | 261.51 |
| chr20 | 61460093 | 61460192 | 100 | *COL9A3* | 141.83 |
| chr20 | 61460252 | 61460351 | 100 | *COL9A3* | 169.81 |
| chr20 | 61460780 | 61460879 | 100 | *COL9A3* | 144.4 |
| chr20 | 61460957 | 61461056 | 100 | *COL9A3* | 112.83 |
| chr20 | 61461095 | 61461194 | 100 | *COL9A3* | 140.11 |
| chr20 | 61461689 | 61461788 | 100 | *COL9A3* | 128.08 |
| chr20 | 61461855 | 61461954 | 100 | *COL9A3* | 52.65 |
| chr20 | 61463474 | 61463573 | 100 | *COL9A3* | 149 |
| chr20 | 61464345 | 61464444 | 100 | *COL9A3* | 230.23 |
| chr20 | 61467240 | 61467339 | 100 | *COL9A3* | 218.73 |
| chr20 | 61467539 | 61467685 | 147 | *COL9A3* | 183 |
| chr20 | 61467808 | 61467907 | 100 | *COL9A3* | 165.2 |
| chr20 | 61468435 | 61468617 | 183 | *COL9A3* | 250.97 |
| chr20 | 61470025 | 61470124 | 100 | *COL9A3* | 403.53 |
| chr20 | 61471894 | 61472084 | 191 | *COL9A3* | 268.01 |
| chr21 | 35821543 | 35821932 | 390 | *KCNE1* | 175.65 |
| chr21 | 37833274 | 37833993 | 720 | *CLDN14* | 191.29 |
| chr21 | 43792830 | 43792929 | 100 | *TMPRSS3* | 141.57 |
| chr21 | 43795825 | 43795977 | 153 | *TMPRSS3* | 116.39 |
| chr21 | 43796650 | 43796795 | 146 | *TMPRSS3* | 131.16 |
| chr21 | 43800224 | 43800323 | 100 | *TMPRSS3* | 98.93 |
| chr21 | 43802091 | 43802343 | 253 | *TMPRSS3* | 103.06 |
| chr21 | 43803142 | 43803307 | 166 | *TMPRSS3* | 175.94 |
| chr21 | 43804051 | 43804150 | 100 | *TMPRSS3* | 299.65 |
| chr21 | 43805518 | 43805643 | 126 | *TMPRSS3* | 105.39 |
| chr21 | 43808512 | 43808635 | 124 | *TMPRSS3* | 121.58 |
| chr21 | 43809038 | 43809154 | 117 | *TMPRSS3* | 97.51 |
| chr21 | 43809548 | 43809927 | 380 | *TMPRSS3* | 332.15 |
| chr21 | 43810036 | 43810146 | 111 | *TMPRSS3* | 139.07 |
| chr21 | 43815430 | 43815529 | 100 | *TMPRSS3* | 114.05 |
| chr22 | 36678714 | 36678831 | 118 | *MYH9* | 169.19 |
| chr22 | 36680139 | 36680311 | 173 | *MYH9* | 229.03 |
| chr22 | 36680449 | 36680557 | 109 | *MYH9* | 417.05 |
| chr22 | 36681167 | 36681375 | 209 | *MYH9* | 154.75 |
| chr22 | 36681704 | 36681827 | 124 | *MYH9* | 140.75 |
| chr22 | 36681905 | 36682005 | 101 | *MYH9* | 100.01 |
| chr22 | 36682764 | 36682892 | 129 | *MYH9* | 126.91 |
| chr22 | 36684298 | 36684459 | 162 | *MYH9* | 296.94 |
| chr22 | 36684773 | 36684985 | 213 | *MYH9* | 339 |
| chr22 | 36685131 | 36685343 | 213 | *MYH9* | 248.03 |
| chr22 | 36688032 | 36688280 | 249 | *MYH9* | 299.96 |
| chr22 | 36689375 | 36689527 | 153 | *MYH9* | 192.41 |
| chr22 | 36689805 | 36689909 | 105 | *MYH9* | 150.55 |
| chr22 | 36690138 | 36690344 | 207 | *MYH9* | 189.62 |
| chr22 | 36690978 | 36691122 | 145 | *MYH9* | 257.01 |
| chr22 | 36691551 | 36691763 | 213 | *MYH9* | 187.19 |
| chr22 | 36692889 | 36693060 | 172 | *MYH9* | 245.65 |
| chr22 | 36694965 | 36695088 | 124 | *MYH9* | 345.1 |
| chr22 | 36696173 | 36696310 | 138 | *MYH9* | 120.96 |
| chr22 | 36696897 | 36697103 | 207 | *MYH9* | 83.12 |
| chr22 | 36697580 | 36697711 | 132 | *MYH9* | 418.62 |
| chr22 | 36698614 | 36698722 | 109 | *MYH9* | 398.39 |
| chr22 | 36700041 | 36700201 | 161 | *MYH9* | 144.34 |
| chr22 | 36701064 | 36701163 | 100 | *MYH9* | 117.33 |
| chr22 | 36701976 | 36702097 | 122 | *MYH9* | 185.6 |
| chr22 | 36702460 | 36702653 | 194 | *MYH9* | 174.32 |
| chr22 | 36705327 | 36705441 | 115 | *MYH9* | 454.6 |
| chr22 | 36708094 | 36708267 | 174 | *MYH9* | 280.62 |
| chr22 | 36710190 | 36710363 | 174 | *MYH9* | 405.29 |
| chr22 | 36712562 | 36712714 | 153 | *MYH9* | 403.51 |
| chr22 | 36714252 | 36714370 | 119 | *MYH9* | 376.66 |
| chr22 | 36715583 | 36715682 | 100 | *MYH9* | 302.57 |
| chr22 | 36716265 | 36716408 | 144 | *MYH9* | 237.69 |
| chr22 | 36716843 | 36716941 | 99 | *MYH9* | 216.04 |
| chr22 | 36717785 | 36717884 | 100 | *MYH9* | 251.22 |
| chr22 | 36718470 | 36718570 | 101 | *MYH9* | 289.84 |
| chr22 | 36722610 | 36722709 | 100 | *MYH9* | 334.9 |
| chr22 | 36723470 | 36723569 | 100 | *MYH9* | 221.75 |
| chr22 | 36737415 | 36737571 | 157 | *MYH9* | 364.1 |
| chr22 | 36744949 | 36745281 | 333 | *MYH9* | 305.79 |
| chr22 | 38097373 | 38097486 | 114 | *TRIOBP* | 356.18 |
| chr22 | 38106434 | 38106573 | 140 | *TRIOBP* | 350.19 |
| chr22 | 38109217 | 38109418 | 202 | *TRIOBP* | 175.13 |
| chr22 | 38111770 | 38111941 | 172 | *TRIOBP* | 402.34 |
| chr22 | 38119192 | 38122510 | 3319 | *TRIOBP* | 322.98 |
| chr22 | 38129305 | 38129419 | 115 | *TRIOBP* | 270.92 |
| chr22 | 38130406 | 38131449 | 1044 | *TRIOBP* | 82.98 |
| chr22 | 38134638 | 38134737 | 100 | *TRIOBP* | 337.72 |
| chr22 | 38136902 | 38137039 | 138 | *TRIOBP* | 314.37 |
| chr22 | 38147758 | 38147857 | 100 | *TRIOBP* | 291.69 |
| chr22 | 38150884 | 38150991 | 108 | *TRIOBP* | 237.13 |
| chr22 | 38151103 | 38151202 | 100 | *TRIOBP* | 274.4 |
| chr22 | 38151557 | 38151666 | 110 | *TRIOBP* | 207.42 |
| chr22 | 38153620 | 38154145 | 526 | *TRIOBP* | 197.31 |
| chr22 | 38155161 | 38155271 | 111 | *TRIOBP* | 274.22 |
| chr22 | 38155424 | 38155534 | 111 | *TRIOBP* | 129.81 |
| chr22 | 38161677 | 38161824 | 148 | *TRIOBP* | 177.49 |
| chr22 | 38164081 | 38164183 | 103 | *TRIOBP* | 82.02 |
| chr22 | 38165035 | 38165194 | 160 | *TRIOBP* | 68.88 |
| chr22 | 38165269 | 38165382 | 114 | *TRIOBP* | 73.28 |
| chr22 | 38167650 | 38167750 | 101 | *TRIOBP* | 97.17 |
| chr22 | 38168608 | 38168769 | 162 | *TRIOBP* | 93.94 |
| chrX | 9621627 | 9621729 | 103 | *TBL1X* | 252.9 |
| chrX | 9622255 | 9622362 | 108 | *TBL1X* | 156.57 |
| chrX | 9652083 | 9652228 | 146 | *TBL1X* | 372.09 |
| chrX | 9656057 | 9656315 | 259 | *TBL1X* | 413.25 |
| chrX | 9659619 | 9659751 | 133 | *TBL1X* | 279.49 |
| chrX | 9660153 | 9660294 | 142 | *TBL1X* | 350.57 |
| chrX | 9661171 | 9661270 | 100 | *TBL1X* | 314.96 |
| chrX | 9661361 | 9661460 | 100 | *TBL1X* | 403.4 |
| chrX | 9665390 | 9665489 | 100 | *TBL1X* | 320.98 |
| chrX | 9673033 | 9673154 | 122 | *TBL1X* | 393.19 |
| chrX | 9677276 | 9677375 | 100 | *TBL1X* | 266.79 |
| chrX | 9677673 | 9677800 | 128 | *TBL1X* | 310.05 |
| chrX | 9679651 | 9679816 | 166 | *TBL1X* | 377.42 |
| chrX | 9682942 | 9683043 | 102 | *TBL1X* | 363.99 |
| chrX | 9684224 | 9684323 | 100 | *TBL1X* | 332.55 |
| chrX | 21755680 | 21755815 | 136 | *SMPX* | 308.49 |
| chrX | 21761867 | 21761954 | 88 | *SMPX* | 384.01 |
| chrX | 21772363 | 21772408 | 46 | *SMPX* | 405.76 |
| chrX | 43809044 | 43809272 | 229 | *NDP* | 419.45 |
| chrX | 43817717 | 43817891 | 175 | *NDP* | 360.23 |
| chrX | 70443558 | 70444409 | 852 | *GJB1* | 131.71 |
| chrX | 82763333 | 82764418 | 1086 | *POU3F4* | 226.2 |
| chrX | 100601487 | 100601648 | 162 | *TIMM8A* | 226 |
| chrX | 100603333 | 100603432 | 100 | *TIMM8A* | 114.79 |
| chrX | 100603521 | 100603652 | 132 | *TIMM8A* | 273.14 |
| chrX | 106871858 | 106871980 | 123 | *PRPS1* | 384.06 |
| chrX | 106882524 | 106882708 | 185 | *PRPS1* | 324.37 |
| chrX | 106884131 | 106884230 | 100 | *PRPS1* | 370.55 |
| chrX | 106885595 | 106885720 | 126 | *PRPS1* | 360.43 |
| chrX | 106888406 | 106888580 | 175 | *PRPS1* | 428.89 |
| chrX | 106890835 | 106890995 | 161 | *PRPS1* | 406.7 |
| chrX | 106893169 | 106893262 | 94 | *PRPS1* | 416.48 |
