# Supplementary material for: Expansion of phenotypic spectrum of MYO15A pathogenic variants to include postlingual onset of progressive partial deafness
Source: BMC Med Genet. 2018 Feb 27;19:29. doi: 10.1186/s12881-018-0541-9 (PMC6389081; doi:10.1186/s12881-018-0541-9)
Supplement: Supplementary file 3 — Table S3. Exome sequencing statistics. The table lists information of human genome reference (v19), customized panel and whole exome sequencing aligned reads from each patient estimated by CalculateHsMetrics of picard tool. (DOCX 29 kb) [file 12881_2018_541_MOESM3_ESM.docx]

**Additional file 3: Table S3. Exome sequencing statistics.** The table lists information of human genome reference (v19), customized panel and whole exome sequencing aligned reads from each patient estimated by CalculateHsMetrics of picard tool.

| Field | SB127_235 | SB127_247 | SB127_277 | SB127_497 | Description |
| --- | --- | --- | --- | --- | --- |
| GENOME_SIZE | 3,095,693,983 | 3,095,693,983 | 3,095,693,983 | 3,095,693,983 | The number of bases in the reference genome used for alignment. |
| BAIT_TERRITORY | 49,984,714 | 49,984,714 | 49,984,714 | 49,984,714 | The number of bases which have one or more baits on top of them. |
| TARGET_TERRITORY | 50,621,019 | 50,621,019 | 50,621,019 | 50,621,019 | The unique number of target bases in the experiment where target is usually exons etc. |
| BAIT_DESIGN_EFFICIENCY | 1.013 | 1.013 | 1.013 | 1.013 | Target terrirtoy / bait territory. 1 == perfectly efficient, 0.5 = half of baited bases are not target. |
| TOTAL_READS | 81,878,408 | 81,966,456 | 79,973,149 | 79,379,920 | The total number of reads in the SAM or BAM file examine. |
| PF_READS | 81,878,408 | 81,966,456 | 79,973,149 | 79,379,920 | The number of reads that pass the vendor's filter. |
| PF_UNIQUE_READS | 79,560,379 | 79,981,010 | 78,112,010 | 77,526,513 | The number of PF reads that are not marked as duplicates. |
| PCT_PF_READS | 1 | 1 | 1 | 1 | PF reads / total reads. The percent of reads passing filter. |
| PCT_PF_UQ_READS | 0.972 | 0.976 | 0.977 | 0.977 | PF Unique Reads / Total Reads. |
| PF_UQ_READS_ALIGNED | 79,560,379 | 79,981,010 | 78,112,010 | 77,526,513 | The number of PF unique reads that are aligned with mapping score > 0 to the reference genome. |
| PCT_PF_UQ_READS_ALIGNED | 1 | 1 | 1 | 1 | PF Reads Aligned / PF Reads. |
| PF_UQ_BASES_ALIGNED | 7,925,874,011 | 7,967,607,467 | 7,782,370,428 | 7,719,572,517 | The number of bases in the PF aligned reads that are mapped to a reference base. Accounts for clipping and gaps. |
| ON_BAIT_BASES | 5,156,034,141 | 5,130,838,044 | 5,046,018,858 | 5,102,330,236 | The number of PF aligned bases that mapped to a baited region of the genome. |
| NEAR_BAIT_BASES | 1,365,043,466 | 1,339,005,568 | 1,305,062,633 | 1,252,566,768 | The number of PF aligned bases that mapped to within a fixed interval of a baited region, but not on a baited region. |
| OFF_BAIT_BASES | 1,404,796,404 | 1,497,763,855 | 1,431,288,937 | 1,364,675,513 | The number of PF aligned bases that mapped to neither on or near a bait. |
| ON_TARGET_BASES | 5,187,871,602 | 5,162,659,689 | 5,077,384,014 | 5,133,667,496 | The number of PF aligned bases that mapped to a targeted region of the genome. |
| PCT_SELECTED_BASES | 0.823 | 0.812 | 0.816 | 0.823 | On+Near Bait Bases / PF Bases Aligned. |
| PCT_OFF_BAIT | 0.177 | 0.188 | 0.184 | 0.177 | The percentage of aligned PF bases that mapped neither on or near a bait. |
| ON_BAIT_VS_SELECTED | 0.791 | 0.793 | 0.795 | 0.803 | The percentage of on+near bait bases that are on as opposed to near. |
| MEAN_BAIT_COVERAGE | 103.152 | 102.648 | 100.951 | 102.078 | The mean coverage of all baits in the experiment. |
| MEAN_TARGET_COVERAGE | 103.355 | 102.842 | 101.114 | 102.277 | The mean coverage of targets. |
| PCT_USABLE_BASES_ON_BAIT | 0.630 | 0.626 | 0.631 | 0.643 | The number of aligned, de-duped, on-bait bases out of the PF bases available. |
| PCT_USABLE_BASES_ON_TARGET | 0.634 | 0.630 | 0.635 | 0.647 | The number of aligned, de-duped, on-target bases out of the PF bases available. |
| FOLD_ENRICHMENT | 40.289 | 39.882 | 40.157 | 40.935 | The fold by which the baited region has been amplified above genomic background. |
| ZERO_CVG_TARGETS_PCT | 0.012 | 0.012 | 0.011 | 0.012 | The fraction of targets that did not reach coverage=1 over any base. |
| FOLD_80_BASE_PENALTY | 1.988 | 2.017 | 2.022 | 2.005 | The fold over-coverage necessary to raise 80% of bases in "non-zero-cvg" targets to the mean coverage level in those targets. |
| PCT_TARGET_BASES_2X | 0.990 | 0.990 | 0.990 | 0.990 | The percentage of all target bases achieving 2X or greater coverage. |
| PCT_TARGET_BASES_10X | 0.984 | 0.984 | 0.983 | 0.983 | The percentage of all target bases achieving 10X or greater coverage. |
| PCT_TARGET_BASES_20X | 0.967 | 0.966 | 0.965 | 0.965 | The percentage of all target bases achieving 20X or greater coverage. |
| PCT_TARGET_BASES_30X | 0.933 | 0.931 | 0.929 | 0.929 | The percentage of all target bases achieving 30X or greater coverage. |
| PCT_TARGET_BASES_40X | 0.880 | 0.876 | 0.872 | 0.873 | The percentage of all target bases achieving 40X or greater coverage. |
| PCT_TARGET_BASES_50X | 0.812 | 0.806 | 0.801 | 0.803 | The percentage of all target bases achieving 50X or greater coverage. |
| PCT_TARGET_BASES_100X | 0.444 | 0.441 | 0.430 | 0.437 | The percentage of all target bases achieving 100X or greater coverage. |
| HS_LIBRARY_SIZE | 557,057,412 | 654,430,304 | 670,374,563 | 662,798,973 | The estimated number of unique molecules in the selected part of the library. |
| HS_PENALTY_10X | 3.055 | 3.129 | 3.114 | 3.029 | The "hybrid selection penalty" incurred to get 80% of target bases to 10X. This metric should be interpreted as: if I have a design with 10 megabases of target, and want to get 10X coverage I need to sequence until PF_ALIGNED_BASES = 10^7 * 10 * HS_PENALTY_10X. |
| HS_PENALTY_20X | 3.074 | 3.145 | 3.130 | 3.045 | The "hybrid selection penalty" incurred to get 80% of target bases to 20X. This metric should be interpreted as: if I have a design with 10 megabases of target, and want to get 20X coverage I need to sequence until PF_ALIGNED_BASES = 10^7 * 20 * HS_PENALTY_20X. |
| HS_PENALTY_30X | 3.087 | 3.160 | 3.145 | 3.060 | The "hybrid selection penalty" incurred to get 80% of target bases to 30X. This metric should be interpreted as: if I have a design with 10 megabases of target, and want to get 30X coverage I need to sequence until PF_ALIGNED_BASES = 10^7 * 30 * HS_PENALTY_30X. |
| HS_PENALTY_40X | 3.106 | 3.176 | 3.160 | 3.075 | The "hybrid selection penalty" incurred to get 80% of target bases to 40X. This metric should be interpreted as: if I have a design with 10 megabases of target, and want to get 40X coverage I need to sequence until PF_ALIGNED_BASES = 10^7 * 40 * HS_PENALTY_40X. |
| HS_PENALTY_50X | 3.129 | 3.192 | 3.176 | 3.090 | The "hybrid selection penalty" incurred to get 80% of target bases to 50X. This metric should be interpreted as: if I have a design with 10 megabases of target, and want to get 50X coverage I need to sequence until PF_ALIGNED_BASES = 10^7 * 50 * HS_PENALTY_50X. |
| HS_PENALTY_100X | 3.224 | 3.274 | 3.262 | 3.170 | The "hybrid selection penalty" incurred to get 80% of target bases to 100X. This metric should be interpreted as: if I have a design with 10 megabases of target, and want to get 100X coverage I need to sequence until PF_ALIGNED_BASES = 10^7 * 100 * HS_PENALTY_100X. |
| AT_DROPOUT | 4.249 | 4.440 | 4.430 | 4.391 | A measure of how undercovered <= 50% GC regions are relative to the mean. For each GC bin [0..50] we calculate a = % of target territory, and b = % of aligned reads aligned to these targets. AT DROPOUT is then abs(sum(a-b when a-b < 0)). E.g. if the value is 5% this implies that 5% of total reads that should have mapped to GC<=50% regions mapped elsewhere. |
| GC_DROPOUT | 5.024 | 4.600 | 4.627 | 4.928 | A measure of how undercovered >= 50% GC regions are relative to the mean. For each GC bin [50..100] we calculate a = % of target territory, and b = % of aligned reads aligned to these targets. GC DROPOUT is then abs(sum(a-b when a-b < 0)). E.g. if the value is 5% this implies that 5% of total reads that should have mapped to GC>=50% regions mapped elsewhere. |
